# The Ku-binding motif is a conserved module for recruitment and stimulation of non-homologous end-joining proteins

**DOI:** 10.1038/ncomms11242

**Published:** 2016-04-11

**Authors:** Gabrielle J. Grundy, Stuart L. Rulten, Raquel Arribas-Bosacoma, Kathryn Davidson, Zuzanna Kozik, Antony W. Oliver, Laurence H. Pearl, Keith W. Caldecott

**Affiliations:** 1 Genome Damage and Stability Centre, School of Life Sciences, University of Sussex, Brighton BN1 9RQ, UK; 2 Cancer Research UK DNA Repair Enzymes Group, Genome Damage and Stability Centre, School of Life Sciences, University of Sussex, Brighton BN1 9RQ, UK

## Abstract

The Ku-binding motif (KBM) is a short peptide module first identified in APLF that we now show is also present in Werner syndrome protein (WRN) and in Modulator of retrovirus infection homologue (MRI). We also identify a related but functionally distinct motif in XLF, WRN, MRI and PAXX, which we denote the XLF-like motif. We show that WRN possesses two KBMs; one at the N terminus next to the exonuclease domain and one at the C terminus next to an XLF-like motif. We reveal that the WRN C-terminal KBM and XLF-like motif function cooperatively to bind Ku complexes and that the N-terminal KBM mediates Ku-dependent stimulation of WRN exonuclease activity. We also show that WRN accelerates DSB repair by a mechanism requiring both KBMs, demonstrating the importance of WRN interaction with Ku. These data define a conserved family of KBMs that function as molecular tethers to recruit and/or stimulate enzymes during NHEJ.

## Abstract (web summary)

Werner syndrome is a progeroid disease characterised by genetic instability due to mutations to the WRN helicase/exonuclease. Here the authors define a novel Ku binding motif (KBM) and show that two such motifs facilitate the involvement of WRN in DNA double-strand break repair.

## Graphical abstract

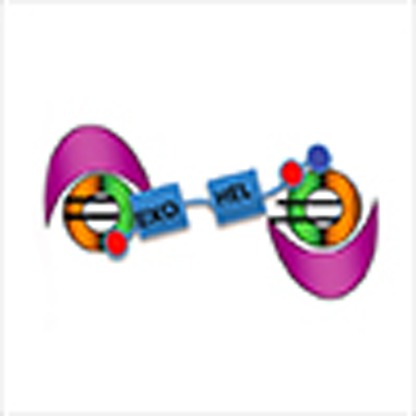

DNA double-strand breaks (DSBs) arise as a consequence of both endogenous and exogenous DNA damage and during normal cellular processes such as the generation of antibody diversity^1^,^2^. DSB repair pathways exist to ensure that chromosomal integrity is maintained but mis-regulation or inappropriate engagement of such pathways can lead to potentially oncogenic translocations, mutagenesis or cell death^3^. Moreover, loss or mutation of non-homologous end joining (NHEJ) factors in mice or humans can result in a range of phenotypes including immunodeficiency, cancer predisposition, neurological defects and embryonic lethality^4^,^5^. Mammalian cells possess two major types of DSB repair pathway; homologous recombination and NHEJ^6^. Homologous recombination employs sister chromatids as a template for accurate repair during S/G_2_ phase of the cell cycle, whereas NHEJ ligates DSB termini directly and can occur throughout the cell cycle.

The core protein factors involved in NHEJ are DNA protein kinase (DNA–PK), XRCC4-like factor (XLF) and XRCC4/DNA ligase IV^7^,^8^,^9^. While these core factors are sufficient to repair DSBs with ligatable termini the repair of most physiologically relevant DSBs require additional protein factors to process the DSB termini before ligation, including nucleases, DNA polymerases and polynucleotide kinase/phosphatase. An increasing number of accessory protein factors have been implicated in NHEJ, many of which appear to interact with DNA–PK^10^. DNA–PK is comprised of a protein kinase catalytic subunit (DNA–PKcs) and Ku heterodimer; the latter being composed of Ku70 and Ku80. Recently, we and others identified a novel Ku-binding peptide motif (now denoted the KBM) of 10–15 amino acids in the accessory protein Aprataxin-and-polynucleotide kinase/phosphatase-like Factor (APLF), which we showed interacts directly with a hydrophobic pocket in the vWA domain of Ku80 (refs 11, 12). Here we have identified and characterized analogous KBMs in two additional NHEJ proteins, revealing this motif to be an evolutionary conserved Ku-binding module. In particular, we identify two KBMs in the exonuclease/helicase mutated in Werner syndrome (WRN) and show that these motifs are employed by WRN to accelerate chromosomal DSB repair, defining the functional importance of these motifs *in vitro* and in cells.

## Results

### A conserved KBM

**Figure 1:**
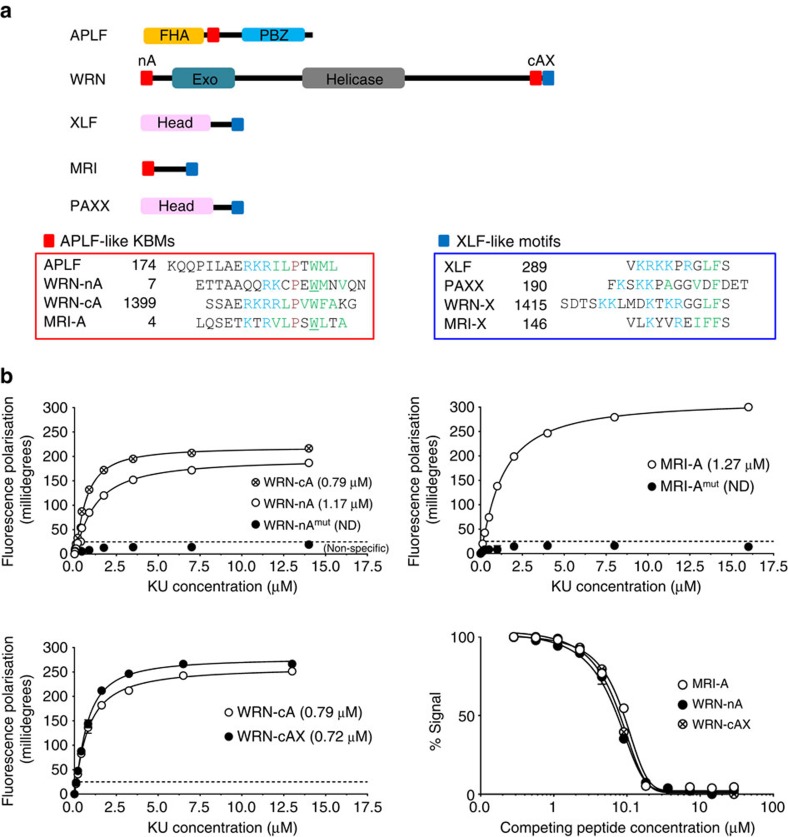
Conserved Ku-binding motifs (KBMs). (**a**) Cartoon of NHEJ proteins containing putative APLF-like KBMs (red squares) and/or the related XLF-like motif (blue squares). Peptide sequences (lower panels) highlight the conserved basic (blue), hydrophobic (green), proline (purple), and tryptophan/phenylalanine (green bold) residues characteristic of these motifs. The tryptophan residues mutated for the fluorescence polarization (FP) assays described below are underlined. (**b**) Top and bottom left panels, FP assays measuring direct interaction between synthetic fluorescein-labeled peptides (100 nM) encoding the indicated KBMs and the indicated concentration of Ku heterodimer (KuΔC). Peptide sequences are those shown in **a**, but additionally preceded at the N terminus by fluorescein-GGYG. Mutant peptides have alanine instead of tryptophan at the positions underlined in **a**. WRN-cAX peptide encodes both the APLF-like KBM (residues 1,399–1,414) and XLF-like motif (residues 1,415–1,432) from the WRN C terminus. Bottom right panel, MRI-A, WRN-nA or WRN-cAX peptides (2.1 μM) were employed in FP competition assays with KuΔC (1 μM) and the indicated concentration (X-axis) of unlabeled APLF KBM peptide. All data points are the mean of three independent experiments (±1 s.d.). *K*_d_ values are indicated in parentheses (±1 s.d.) unless too weak to be determined (‘ND').

The interaction between APLF and Ku80 was previously mapped to a conserved motif in APLF of 10–15 amino acids, denoted the KBM^11^,^12^. PSI-BLAST^13^ analysis using this sequence, and subsequent additional searching by eye, suggested that similar KBMs are present at the N terminus and C terminus of WRN protein; the DNA helicase and exonuclease mutated in Werner syndrome and an established partner of Ku (Fig. 1a)^14^,^15^. We also identified a putative KBM in Modulator of retroviral infection homologue (MRI, C7orf49); a poorly characterized protein that we recovered in a yeast two-hybrid screen using the Ku80 vWA-like domain as bait (Fig. 1a). MRI was reported previously to interact with Ku and to stimulate NHEJ, *in vitro*^16^. Similar to the KBM in APLF^11^, the putative KBMs in WRN and MRI are conserved among vertebrate species (Supplementary Fig. 1). Our analyses also revealed a distinct but related motif at the C terminus of XLF, Paralog of XRCC4 and XLF (PAXX), WRN and MRI, which we denoted the XLF-like motif (Fig. 1a, right). Interestingly, the XLF-like motif in WRN is present in tandem with the putative C-terminal KBM, raising the possibility that these motifs function cooperatively.

### APLF-like KBMs bind Ku by a common mechanism

To examine Ku binding by the putative KBMs and XLF-like motifs, we employed recombinant Ku heterodimer and fluorescent peptides spanning these domains in fluorescence polarization assays. We employed recombinant Ku70/Ku80ΔC heterodimer (denoted KuΔC) lacking the flexible Ku80 C-terminal helical domain for these experiments, since KuΔC exhibits greater structural homogeneity than does full-length Ku heterodimer^11^,^17^,^18^,^19^. Similar to the KBM in APLF, which binds Ku with an affinity of ∼0.6 μM (ref. 11), peptides spanning the APLF-like KBMs from WRN or MRI bound KuΔC with *K*_d_ values of 0.5–1.7 μM (Fig. 1b, top panels). Mutation of the conserved tryptophan in the APLF-like KBMs greatly reduced or ablated KuΔC interaction, suggesting that these motifs share a common mechanism of Ku80 binding (Fig. 1b, top panels). Indeed, a peptide encoding the APLF KBM competed efficiently in KuΔC binding assays with peptides encoding each of the three APLF-like KBMs from WRN and MRI, suggesting that these motifs compete for the same hydrophobic pocket in the Ku80 vWA-like domain that binds APLF^11^ (Fig. 1b, bottom right). In contrast to the APLF-like KBMs none of the peptides spanning the XLF-like motifs interacted with KuΔC heterodimer in fluorescence polarization assays (Supplementary Fig. 2a), and the XLF-like motif in WRN also failed to improve KuΔC binding by the adjacent C-terminal KBM (Fig. 1b, bottom left).

**Figure 2:**
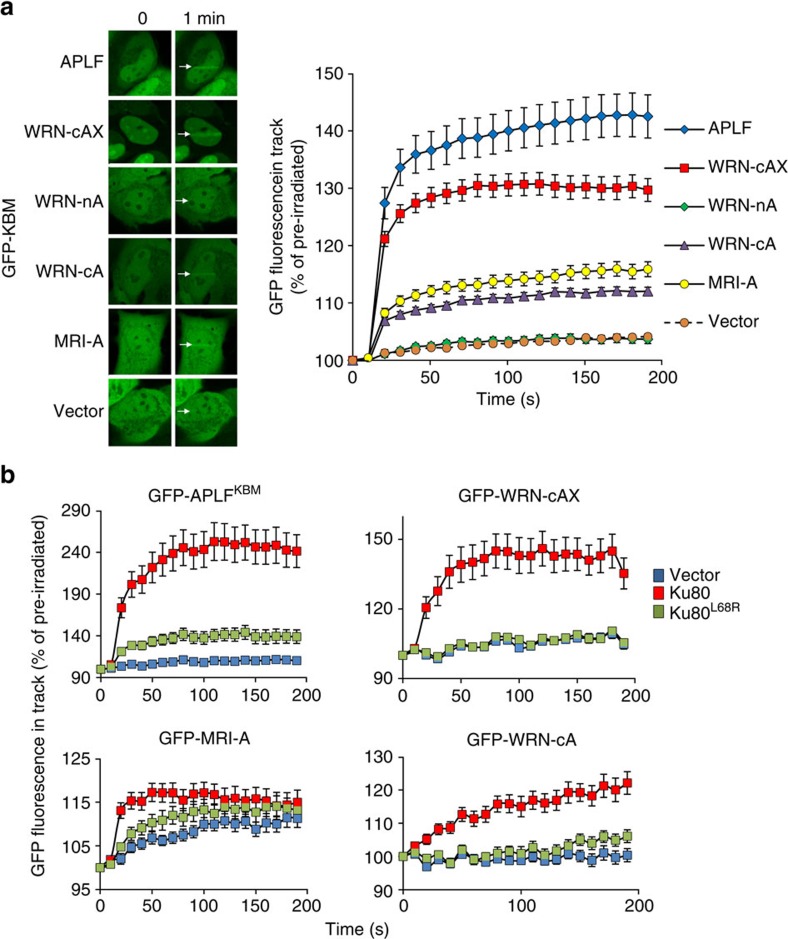
KBM accumulation at sites of UVA laser-induced chromosome damage. (**a**) U2-OS cells were transiently transfected with expression constructs encoding GFP alone (Vector) or the indicated GFP-tagged KBM and subjected to UVA laser-induced micro-irradiation. The expressed peptide sequences for each KBM were APLF (177–193), WRN-cAX (1,399–1,432), WRN-nA (10–23), WRN-cA (1,399–1,414), MRI-A (6–19). Images were captured immediately before and at 10 s intervals following treatment. Representative images are shown on the left and quantified data on the right. (**b**) *Ku80*^*−/−*^ mouse embryonic fibroblasts (MEFs) were co-transfected with expression constructs encoding the GFP-tagged KBM from APLF or the indicated GFP-tagged APLF-like KBMs from WRN or MRI, mRFP-Ku70, and either mRFP (‘vector'), mRFP-Ku80 or mRFP-Ku80^L68R^. Cells were micro-irradiated with UVA as above. All data are the mean GFP fluorescence (±s.e.m.) in the laser track relative to the mean GFP fluorescence before irradiation (set at 100%) from >20 cells per experiment.

To examine whether the APLF-like KBMs are sufficient to bind Ku in cells, we employed UVA laser microirradiation. With the exception of the N-terminal KBM from WRN (‘WRN-nA') each of the green fluorescent protein (GFP)-tagged KBMs accumulated at sites of UVA-induced chromosome damage in U2-OS cells, albeit with different efficiencies, and did so with similar kinetics to red fluorescent protein (RFP)-Ku80 (Fig. 2a and Supplementary Fig. 3). The XLF-like motifs from XLF and PAXX also accumulated at sites of UVA-induced chromosome damage, albeit relatively weakly, despite their inability to bind Ku in fluorescence polarization assays (Supplementary Fig. 2b). In contrast, the XLF-like motifs from MRI and WRN were unable to accumulate at sites of UVA laser damage (Supplementary Fig. 2b), although the latter did increase accumulation of the adjacent C-terminal KBM (Fig. 2a; compare ‘WRN-cA' and ‘WRN-cAX'). Importantly, recruitment of the GFP-tagged APLF-like KBMs to sites of UVA laser damage was reduced by mutation of the Ku80 vWA-like domain (L68R) that we showed previously binds the APLF KBM^11^ (Fig. 2b), further suggesting that each of the KBMs interact with the same site in Ku80.

### The WRN C-terminal tandem domains bind Ku cooperatively

**Figure 3:**
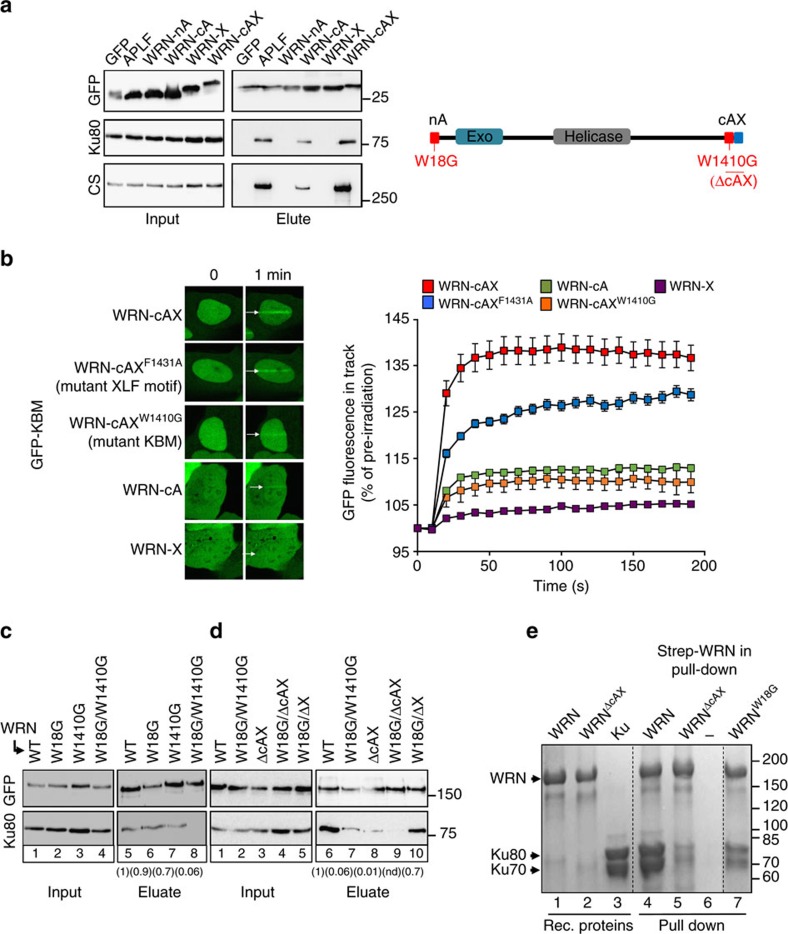
The WRN C-terminal KBM and XLF-like motif bind Ku protein complexes cooperatively. (**a**) HEK293T cells were co-transfected with expression constructs encoding GFP or the indicated GFP-tagged KBMs and GFP-tagged proteins recovered using GFP-TRAP beads. Aliquots of the input and eluate samples were fractionated by SDS–PAGE and immunoblotted for GFP, Ku80 and DNA–PKcs (CS). Right, cartoon depicting WRN and the position of the KBMs and XLF-like motif and the mutations employed in these experiments. (**b**) HEK293T cells were transfected with expression constructs encoding the indicated wild-type or mutated GFP-tagged WRN C-terminal KBM, XLF-like motif (‘X'), or KBM plus XLF-like motif in tandem. Cells were micro-irradiated with UVA as in Fig. 2. Representative images (left) and quantification (right) are shown. All quantified data are the mean GFP fluorescence (±s.e.m.) in the laser track relative to the mean GFP fluorescence before irradiation (set at 100%) from >20 cells per experiment. (**c**,**d**) Expression constructs encoding full-length wild-type (‘WT') GFP–WRN or derivatives harbouring the indicated point mutations in the N-terminal KBM (W18G), C-terminal KBM (W1410G) or deleted C-terminal tandem domain (ΔcAX) or XLF-like motif (ΔX ) were transfected into HEK293T cells and recovered using GFP-TRAP beads. Input and eluates were immunoblotted for GFP and Ku80. Numbers in parentheses are the fraction of Ku co-precipitated by the indicated GFP-tagged WRN protein, relative to wild-type WRN, quantified by ImageJ. Data are from two to six independent experiments, except for W18G/ΔcAX in which Ku recovery was too low to be determined (‘nd'). (**e**) Direct interaction of purified full-length Strep-tagged WRN with recombinant human Ku. Recombinant Strep-tagged WRN, WRN^ΔcAX^ or WRN^W18G^ was immobilized on Streptavidin Mag sepharose beads and incubated with recombinant Ku heterodimer. Aliquots of the recombinant proteins employed in the experiment are shown on the left (lanes 1–3) and proteins pulled down by the indicated Strep-tagged WRN protein are shown on the right (lanes 4–7). Lane 6 contains the proteins recovered in a control pull-down that lacked Strep-tagged WRN. Proteins were fractioned by SDS–PAGE and stained with Coomassie Blue.

Mutations in WRN protein result in Werner syndrome, a rare genetic disease characterized by genome instability, premature ageing and cancer^20^,^21^. WRN is a member of the RecQ family of helicases and is involved in multiple DNA repair processes^20^. Since WRN possesses multiple KBMs and also an XLF-like motif, we addressed the role and relative importance of these for Ku binding. Once again, as described above, the C-terminal KBM targeted GFP to sites of UVA laser-induced damage when expressed in cells as a fusion peptide and did so more efficiently if present together with the adjacent XLF-like motif (Fig. 3b, compare ‘WRN-cA' and ‘WRN-cAX'). Moreover, this accumulation was reduced if either the C-terminal KBM or XLF-like motif were mutated, further suggesting that these two motifs function cooperatively. Similar results were observed in pull-down experiments, in which GFP-tagged KBM co-precipitated Ku protein complexes from cell extract more efficiently if present in tandem with the XLF-like motif, despite the latter being unable to co-precipitate Ku complexes by itself (Fig. 3a). In addition, whereas co-precipitation of Ku by full-length GFP–WRN was reduced by only ∼30% by mutation of either the C-terminal KBM or the XLF-like motif separately (Fig. 3c, lane 7 and Fig. 3d, lane 10), it was reduced by >95% by deletion of the entire C-terminal tandem domain (Fig. 3d, lane 8). Notably, recombinant Ku was also co-precipitated by purified full-length recombinant Strep-tagged WRN *in vitro*, and this co-precipitation was again greatly reduced by deletion of the C-terminal tandem domain (Fig. 3e). This experiment confirms that WRN and Ku interact directly and do so in a manner that is mediated primarily by the C-terminal tandem domain.

### The N-terminal KBM cooperates with WRN exonuclease

In contrast to the C-terminal KBM, the N-terminal KBM was unable to accumulate at sites of UVA laser-induced chromosome damage or co-precipitate Ku protein complexes if over-expressed by itself as a GFP-fusion protein (Figs 2a and 3a; ‘WRN-nA'). Mutation of the N-terminal KBM reduced Ku co-precipitation by full-length GFP–WRN by only ∼10% (Fig. 3c, lane 6), and reduced Ku co-precipitation by Strep-tagged WRN *in vitro* to a lesser extent than deletion of the C-terminal tandem domain (Fig. 3e). Nevertheless, mutation of the N-terminal KBM further reduced Ku co-precipitation to ∼7% of normal if combined with mutation of the C-terminal KBM (Fig. 3d, lane 7), and to almost undetectable levels if combined with deletion of the C-terminal tandem domain (Fig. 3d, lane 9). We thus conclude that both the N-terminal KBM and the C-terminal tandem domain contribute to the stable interaction of WRN with Ku, with the C-terminal tandem domain contributing the most.

**Figure 4 f4:**
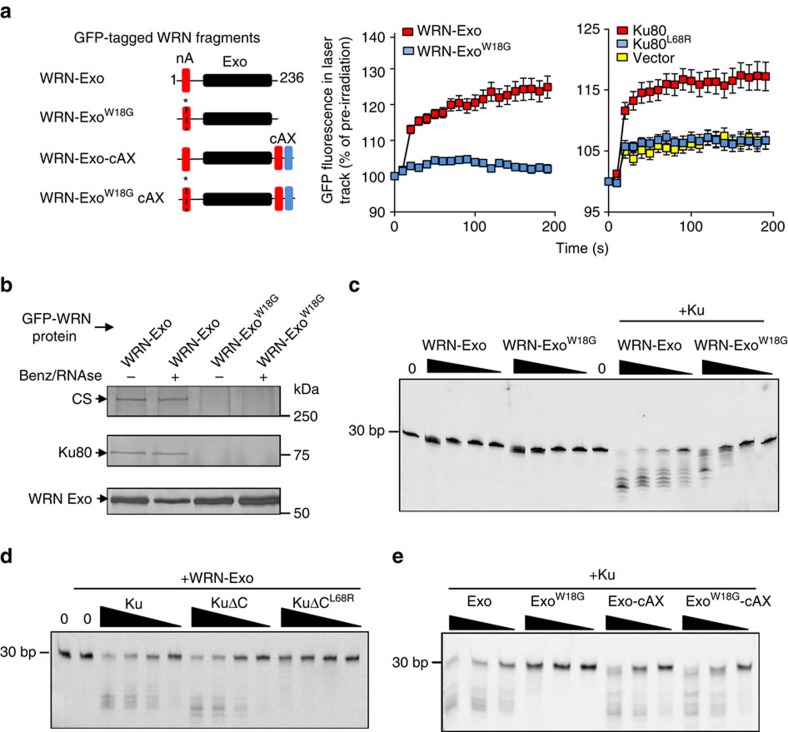
The WRN N-terminal KBM promotes WRN exonuclease activity. (**a**) Left, cartoon illustrating the GFP-tagged truncated recombinant WRN proteins employed in these experiments. The WRN N-terminal (‘nA') and C-terminal (‘cA') KBMs are indicated by red boxes and XLF-like motif (‘X') by a blue box. The exonuclease domain is indicated by a black box, and the position of the KBM mutation (W18G) by an asterisk and dotted line. Middle, U2-OS cells transiently expressing the indicated recombinant GFP-tagged WRN protein were imaged for GFP before and after UVA microirradiation, as in Fig. 2. Right, *Ku80*^*−/−*^ MEFs transiently co-expressing GFP-tagged WRN-Exo, RFP-Ku70, and either RFP (vector), RFP-Ku80 or RFP-Ku80^L68R^ as indicated were micro-irradiated as in Fig. 1. Data are the mean GFP fluorescence (±s.e.m.) in the laser track relative to the mean GFP fluorescence before irradiation (set at 100%) from >20 cells per experiment. (**b**) The indicated GFP-tagged WRN proteins were recovered from transiently transfected HEK293T cell lysates pre-treated or not as indicated with Benzonase and RNAse in pull-down assays using GFP-TRAP beads. Aliquots of the bead eluate were fractionated by SDS-PAGE and silver stained to detect GFP-WRN, GFP-WRNW18G, Ku80, and DNA-PKcs (‘CS'). (**c**) Cy3-labeled 30 bp duplex oligonucleotide (20 nM) with a 5′ overhang was incubated with 500, 100, 20 or 5 nM HIs-tagged WRN-Exo or WRN-Exo^W18G^ in the absence or presence of 100 nM Ku heterodimer (Ku70/Ku80, ‘Ku') and 5 mM MgCl_2_. Exonuclease products were resolved on a 16% TBE-Urea gel. (**d**) Exonuclease assays were conducted as above in the presence of 5 mM MgCl_2_ using 10 nM His-tagged WRN-Exo and 100, 20, 4 or 0.8 nM of either Ku heterodimer (Ku70/Ku80; ‘Ku'), KuΔC heterodimer (Ku70/Ku80ΔC; ‘KuΔC'), or mutant KuΔC heterodimer harbouring the Ku80 mutation, L68R (KuΔC^L68R^). (**e**) Exonuclease assays were conducted as above using 100, 20 and 4 nM of the indicated His-tagged WRN protein and 10 nM wild-type Ku heterodimer (Ku70/Ku80; ‘Ku') in 5 mM Mg^2+^.

Given the close proximity of the N-terminal KBM and exonuclease domain (Fig. 1a), we considered the possibility that these domains might function cooperatively. In support of this, in contrast to GFP-tagged N-terminal KBM alone (see above), a GFP-tagged fragment encoding both the KBM and the exonuclease domain (WRN^1–236^; denoted ‘WRN-Exo') accumulated at sites of UVA laser-induced chromosome damage in human U2-OS cells (Fig. 4a, left and middle). Importantly, however, WRN-Exo accumulation was diminished by the mutation of either the N-terminal KBM (Fig. 4a, middle) or the KBM-binding site in Ku80 (Fig. 4a, right), indicating that KBM-mediated interaction with Ku was required for WRN-Exo accumulation at chromosome damage. Similarly, GFP-tagged WRN-Exo co-precipitated Ku protein complexes in pull-down experiments, and this required the KBM because the W18G mutation greatly reduced or ablated Ku co-precipitation (Fig. 4b). Notably, WRN-Exo co-precipitated Ku even in the presence of DNAse and RNAse in these experiments, suggesting that the interaction between these proteins is not mediated by nucleic acid. Similar results were observed in yeast two-hybrid assays, in which WRN-Exo transactivated a β-galactosidase reporter gene if co-expressed with Ku80 (but not Ku70) in a manner dependent on both the KBM in WRN-Exo and the KBM-binding site in Ku80 (Supplementary Fig. 4).

Next, we examined the role of the N-terminal KBM in the stimulation of WRN exonuclease activity by Ku^14^,^15^. WRN-Exo was stimulated by either full-length Ku heterodimer or the truncated KuΔC heterodimer employed in our fluorescence polarization assays, and this stimulation was greatly reduced by mutation of either the KBM (Fig. 4c) or the KBM-binding site in Ku80 (Fig. 4d). This did not reflect a non-specific effect of the KBM mutation on WRN exonuclease activity; however, because wild-type and mutant WRN-Exo were equally active if stimulated independently of Ku by replacing magnesium with manganese in the assay (Supplementary Fig. 5)^22^. Finally, fusion of the tandem peptide from the C terminus of WRN to the C terminus of WRN-Exo^W18G^ rescued stimulation by Ku, suggesting that the KBM stimulates WRN exonuclease by acting as a position-independent molecular tether (Fig. 4e).

### The WRN KBMs accelerate chromosomal DSB repair

**Figure 5:**
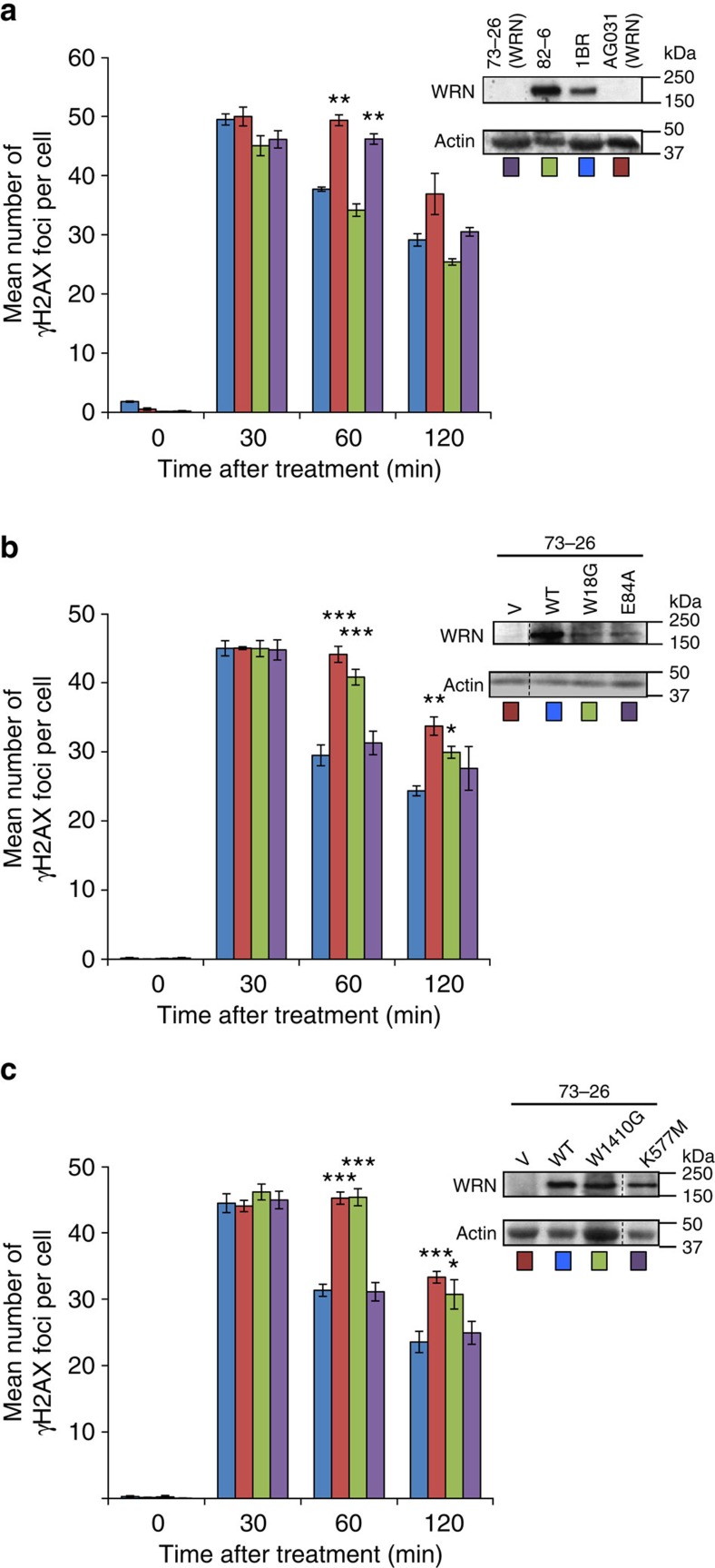
WRN KBMs accelerate DSB repair. (**a**) Confluence-arrested (G0/G1) hTERT-immortalised fibroblasts from two WRN patients (73–26 and AG03141) and normal controls (82–6 and 1BR) were treated with γ-rays (2 Gy) and γH2Ax foci counted at the time-points indicated. Inset, Actin and WRN protein levels in the indicated cell lines. (**b**) WRN cells (73–26) stably transduced with empty vector (V) or vector encoding wild-type (WT) WRN, WRN^W18G^ harbouring a mutated N-terminal KBM (W18G), or WRN harbouring a mutated exonuclease domain WRN^E84A^ (E84A), were examined for DSB repair rates as described above. (**c**) Werner syndrome cells (73–26) stably transduced with empty vector (V) or vector encoding WT WRN, WRN^W1410G^ harbouring a mutated C-terminal KBM (W1410G), or WRN harbouring a mutated helicase domain (WRN^K577M^) were examined as above. Data points are the mean (±s.e.m.) number of foci per cell from four independent experiments. *P<0.05, **P<0.01, ***P<0.001 by paired *t*-test when compared with WT cells.

WRN has previously been implicated in NHEJ by various biochemical and cellular assays^14^,^15^,^23^,^24^,^25^,^26^, but a role in promoting chromosomal DSB repair has not been demonstrated. We showed recently that the interaction of the APLF KBM with Ku accelerates NHEJ, as measured using γH2AX as a surrogate marker of DSBs^11^. Given the similarity of the APLF-like KBMs, we examined whether this was also the case for WRN. Indeed, we observed a small but significant reduction in NHEJ rate in Werner Syndrome cells arrested in G_0_, as suggested by the slower loss of γH2AX foci in these cells following ionizing radiation (Fig. 5a). We employed cells arrested in G_0_ in these experiments to avoid measuring DSBs induced in S/G2 phase, which are substrates for homologous recombination-mediated repair. To our knowledge this is the first report of a reduced rate of chromosomal NHEJ in Werner Syndrome cells. The slower loss of γH2AX foci reflected the loss of WRN because it was complemented by expression of wild-type recombinant human WRN (Fig. 5b,c). In contrast, this defect was not complemented by recombinant WRN protein harbouring mutations in either of the two KBMs, confirming the importance of these motifs for WRN functionality during NHEJ (Fig. 5b,c). However, WRN protein harbouring a mutated exonuclease or helicase domain was still able to complement the defect, suggesting that the acceleration of NHEJ detected here reflects the scaffolding function of WRN^23^ rather than its catalytic activity.

## Discussion

The NHEJ accessory factor APLF possesses a short conserved peptide motif denoted the KBM that interacts with the vWA-like domain in Ku80 (refs 11, 12). Here we show that the KBM is present and conserved in several other NHEJ proteins, including two in WRN protein and one in MRI; a poorly characterized protein that interacts with Ku and promotes NHEJ by an unclear mechanism^16^,^27^. Each of these KBMs interact with Ku heterodimer with sub/low micromolar affinity *in vitro*, and do so by interacting with the same hydrophobic pocket in the Ku80 vWA domain that binds APLF. Each of the KBMs also accumulate at cellular sites of laser-induced chromosome damage in a Ku80- and vWA-dependent manner when expressed as a GFP-tagged peptide, with the exception of the KBM present at the N terminus of WRN, which accumulates at these sites cooperatively with the adjacent exonuclease domain. Intriguingly, database searches using Pattinprot and the core minimal KBM sequence (R-X-X-P-X-W) identified more than 600 proteins with this motif (Supplementary Data 1). More sophisticated bioinformatic analyses and experimental validation are needed to identify which of these are true KBMs.

We also identified a motif in XLF, WRN, PAXX and MRI that is similar in sequence to the KBM but which is structurally and functionally distinct, and which we denoted the XLF-like motif. KBMs and XLF-like motifs are similar in sequence in that both are comprised of a basic patch followed by a highly conserved aromatic residue, but they differ in several key respects. Whereas KBMs possess a highly conserved proline and tryptophan the XLF-like motifs possess a conserved phenylalanine. In addition, whereas KBMs are found at different locations the XLF-like motifs are typically present at protein C termini. Finally, in contrast to KBMs, none of the XLF-like motifs interacted measurably with Ku heterodimer in fluorescence polarization assays. This is surprising, because the XLF and PAXX motifs promote accumulation of the full-length proteins at sites of laser-induced chromosome damage in a Ku-dependent manner and/or associate with Ku complexes *in vitro*^28^,^29^,^30^. Consequently, we suggest that XLF-like motifs associate with Ku complexes only in the presence of DNA and/or other cellular protein/s. This idea is consistent with a previous report that mutation of this motif in XLF influences binding to DNA^31^.

Werner syndrome is a progeroid disease characterized by premature ageing, genetic instability and predisposition to cancer^20^,^21^, and WRN protein is implicated in multiple aspects of DNA metabolism including telomere maintenance, base-excision repair, homologous recombination, replication fork processing and NHEJ (reviewed in ref. 20). Intriguingly, WRN protein possesses two KBMs and an XLF-like motif, with the N-terminal KBM located next to the exonuclease domain and the C-terminal KBM located in tandem with the XLF-like motif. WRN interacts with multiple protein components of these pathways including MRN nuclease, RAD51, XPG and Ku heterodimer^14^,^15^,^32^,^33^,^34^. The interaction with Ku was reported to occur towards both the N and C termini of WRN^14^,^15^,^35^, and our identification of N- and C-terminal KBMs has fine-mapped these interactions and allowed us to disrupt them individually or together. All of the KBM interactions with Ku detected to date are with the vWA-like domain of Ku80. This is in agreement with two of the above reports, which also concluded that the N and C termini of WRN interact with Ku80 (ref. 15), but disagrees with the study of Karmakar *et al*.^35^ in which the WRN N terminus was reported to interact with Ku70. The source of this discrepancy is not clear but our conclusion that Ku80 is the partner of both the N-terminal and C-terminal KBMs is based on a variety of biochemical, cellular and yeast two-hybrid experiments.

Whereas mutation of the individual KBMs in full-length WRN did not greatly reduce the interaction with Ku, as measured by co-immunoprecipitation experiments, pair-wise mutation or deletion greatly reduced or ablated it. However, only the C-terminal KBM was able by itself to accumulate at sites of chromosomal damage or efficiently co-precipitate Ku from cell extract, suggesting that this KBM is the major contributor to stable Ku binding by WRN. It is not clear why this was not reflected in our fluorescence polarization assays *in vitro*, in which the two KBMs interacted with Ku with similar affinities. Importantly, the adjacent XLF-like motif functioned cooperatively with the C-terminal KBM, greatly enhancing Ku interaction and accumulation at chromosome damage. It is possible that the XLF-like motif simply promotes Ku binding by the adjacent KBM, although we note that it did not increase the affinity of the KBM for Ku in fluorescence polarization assays, *in vitro*. Rather, we suggest that the XLF-like motif interacts with another component of DNA–PK complexes that is positioned near the KBM-binding site in Ku80, such as DNA–PKcs. An interaction between WRN and DNA–PKcs has been reported previously^24^, and in our experiments the XLF-like motif promoted co-precipitation of DNA–PKcs to a greater extent than Ku when present either in tandem with the C-terminal KBM (Figs 3a, [Fig f4]). While more experiments are required to confirm this idea, we suggest that the XLF-like motif in WRN interacts directly with the catalytic subunit of DNA–PK, thereby promoting the assembly of more stable DNA–PK protein complexes.

The N-terminal KBM in WRN was unable by itself to accumulate at sites of chromosome damage or to precipitate Ku from cell extract, despite the affinity of this KBM for Ku *in vitro* being similar to that of the C-terminal KBM. However, the N-terminal KBM both accumulated at sites of UVA laser damage and promoted co-precipitation of Ku if present together with the adjacent exonuclease domain. These data suggest that while the N-terminal KBM possesses intrinsic Ku-binding activity it requires the adjacent exonuclease domain for Ku binding in cells and for accumulation at sites of chromosome damage. The reason for this difference between *in vitro* and cellular functionality is currently unclear, but nevertheless the cooperativity between the KBM and exonuclease domain extended to the activity of the latter, which was stimulated by Ku in a largely KBM-dependent manner. Intriguingly, fusion of the C-terminal KBM to the C terminus of the WRN exonuclease domain also supported Ku-dependent stimulation of WRN exonuclease activity, even in the absence of a functional N-terminal KBM. This suggests that the KBMs act in an orientation-independent but proximity-dependent manner to tether the WRN exonuclease domain to Ku–DNA complexes.

WRN has been implicated in NHEJ previously. For example, the interaction with Ku stimulates WRN exonuclease activity on a variety of DSB termini, including those harbouring different types of recessed termini and termini harbouring oxidized nucleotides^14^,^15^,^36^. This is consistent with a role for WRN in processing DSB termini during NHEJ in advance of gap filling and DNA ligation. A number of phenotypes are also consistent with aberrant NHEJ in WRN syndrome cells, such as mild hypersensitivity to ionizing radiation^24^, reduced accuracy and joining efficiency at plasmid-borne DSBs^23^,^25^, and elevated deletion sizes at the chromosomal *HPRT* locus^37^. However, to our knowledge, the current work is the first in which an impact of WRN on the rate of chromosomal NHEJ has been observed. While this phenotype is similar to that reported for APLF, another KBM-mediated partner of Ku80, it is unique in that it was detected only in cells arrested in G_0_. We do not yet understand the reason for this observation, but one possibility is that the role detected here for WRN is redundant with other proteins in other cell cycle phases. Surprisingly, although both WRN KBMs were required for acceleration of NHEJ, the catalytic activity of WRN was not required. This does not rule out an involvement of WRN catalytic activity during NHEJ, because it is possible that the fraction of DSBs requiring this activity is too small to detect or that other enzymes can also provide this activity. Nevertheless, our data suggest that the acceleration of NHEJ that we have detected in this work reflects an impact of WRN on the structure and stability of NHEJ protein complexes. Such a structural role for WRN has been suggested previously, in which WRN stabilizes DNA–PK complexes and protects DSB termini from excessive degradation by other nucleases^23^.

**Figure 6:**
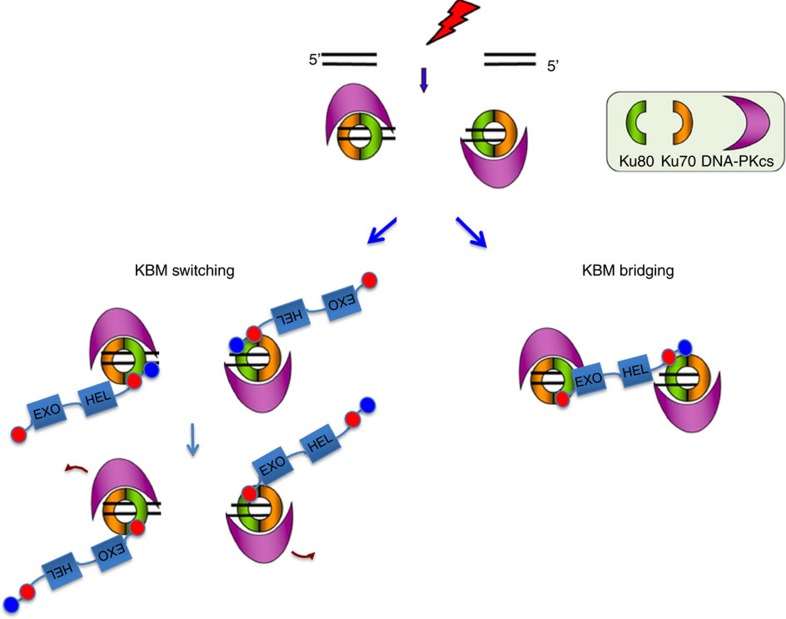
A Model for WRN KBM function during NHEJ. Top, DNA–PK holoenzyme binds to a DSB. Bottom left, WRN is recruited into DNA–PK complexes by high affinity interaction between the C-terminal KBM (red circle) and the hydrophobic pocket in the vWA domain of Ku80. The XLF-like motif (blue circle) functions cooperatively, perhaps stabilizing the association of Ku with DNA–PKcs. Following autophosphorylation, DNA–PKcs dissociates and the C-terminal KBM is replaced by the N-terminal KBM to stimulate WRN 3′-exonucease activity. Bottom right, The N-terminal and C-terminal KBMs bind two Ku molecules simultaneously, bridging the DSB. Note that WRN may fulfil both enzymatic and structural roles during NHEJ.

On the basis of these data, we propose the following model (Fig. 6). We suggest that the N- and C-terminal KBMs enable WRN to interact with Ku80 and thereby promote the stability of DNA–PK protein complexes. The two KBMs may interact with Ku80 simultaneously, sequentially or both. For example, a high-affinity interaction of the C-terminal KBM tandem domain could tether WRN to DNA–PK, with the N-terminal KBM either displacing the C-terminal KBM from Ku80 when exonuclease activity is required for end processing (Fig. 6, left) or, alternatively, interacting with a second molecule of Ku on the opposite DSB terminus to bridge the break (Fig. 6, right). Either of these possibilities can explain the need to mutate or delete both KBMs to greatly reduce or ablate Ku interaction, and the requirement for both KBMs for normal rates of NHEJ.

## Methods

### WRN cells

The hTERT-immortalised fibroblast cell lines ‘73–26' (Werner syndrome)^24^ and wild-type sibling control (‘82–6')^24^ were kindly provided by Judy Campisi (Buck Institute, CA), and the Werner syndrome cell line AG03141 (ref. 38) was kindly provided by David Kipling (Cardiff University). Retroviruses encoding wild-type or mutant human WRN protein were packaged in GP2–293 cells using the Retro-X Universal Packaging System (Clontech) according to the manufacturer's instructions. GP2–293 supernatants were used to transduce 73–26 hTERT cells in the presence of 4 μg ml^−1^ hexadimethrine bromide (Polybrene, Sigma) for 24 h. Following three successive rounds of transduction, cells were selected in 0.5 mg ml^−1^ G418 for 4–6 weeks. Resulting cultures were screened for WRN expression by western blotting using mouse anti-WRN (Abcam 66601, 1:500). All cell lines were tested and found to be mycoplasma-free before use.

### Plasmids

Primers employed for cloning and mutagenesis are detailed in Supplementary Table 1. pET16b-WRN-Exo, encoding WRN residues 1–236 (UniProt accession number Q14191) and including a C-terminal octahistidine tag, was generated by PCR using pEGFP-C3-WRN (a gift from Will Bohr) as a template and subsequent subcloning of the PCR product into the *Nco*I and *Xho*I sites of pET16b. GST-fusion or enhanced GFP (eGFP)-fusion peptides encoding the KBMs or XLF-like motifs WRN-nA (KBM sequence; LETTAAQQRKCPEWMNVQ), WRN-cA (KBM sequence: TSSAERKRRLPVWFAK), WRN-X (KBM sequence: SKKLMDKTKRGGLFS), MRI-A (KBM sequence: SETKTRVLPSWLTA), MRI-X (KBM sequence; VLKYVREIFFS), XLF (KBM sequence; VKRKKPRGLFS) and PAXX (KBM sequence; FKSKKPAGGVDFDET) were generated by annealing appropriate complementary oligonucleotides and ligation into the *Bam*HI/*Xho*I sites of pGEX6p1 or the *Bgl*II/*Sal*I sites of pEGFP-C1, respectively. For GST-fused and eGFP-fused WRN-cAX, residues 1,399–1,432 (SSAERKRRLPVWFAKSKKLMDKTKRGGLFS) were amplified by PCR and subcloned as above. GFP-tagged WRN-Exo-cAX was generated by PCR amplification of WRN-Exo (see above) and insertion of the resulting *Bgl*II fragment into the *Bam*HI site of pGEX6-cAX. To generate pEGFP-WRN encoding full-length WRN, a stop codon was introduced by site-directed mutagenesis of pEGFP-C3-WRN after S1432, to remove exogenous plasmid-derived C-terminal residues present in pEGFP-C3-WRN (see above)^39^. Alternatively, to generate pGFP-WRN^ΔcAX^, a stop codon was introduced at position S1399. Derivatives harbouring point mutations were generated by site-directed mutagenesis using the primers in Supplementary Table 1. pmRFP-Ku70, pmRFP-Ku80 and pmRFP-Ku80^L68R^ were generated by subcloning from pGFP-Ku70, pGFP-Ku80 and pGFP-Ku80^L68R^ (ref. 11). pLXSN, pLXSN-WRN, pLXSN-WRN^E84A^ and pLXSN-WRN^K577M^ were kind gifts from Junko Oshima^23^. For yeast two-hybrid plasmids, the *Nco*I/*Xho*I fragments from pET16b-WRN-Exo and pET16b-WRN-Exo^W18G^ were ligated into the *Nco*I/*Sal*I sites of pGBKT7. pACT2-Ku80 vWA (encoding residues 1–258) was constructed using the *Xho*I fragment from pACT Clone 5, which was recovered from a previous pACT human complementary DNA library screen using APLF as bait^11^. pACT2-Ku80 vWA^L68R^ mutant was also subcloned in this way. pACT2-Ku70 vWA was cloned by PCR amplification of a fragment encoding amino acids 1–272 of Ku70 and insertion into the *Bam*HI and *Xho*I restriction sites of pACT2.

### Yeast two-hybrid experiments

For interaction analysis, yeast Y190 cells were co-transformed with the indicated pACT2 and pGBKT7 plasmids and selected on minimal media plates lacking leucine and tryptophan. Transformed cells were screened for activation of the LacZ reporter gene by β-galactosidase filter lift assays^11^.

### Recombinant proteins

Strep-tagged WRN, Ku, KuΔC and KuΔC^L68R^ were expressed and purified from insect cells using a baculovirus expression system and purified using immobilized metal-chelate chromatography and gel filtration^11^. Strep-tagged WRN was purified using an affinity Strep-Tactin Superflow Plus cartridge (Qiagen) followed by Superose 6 gel filtration. KuΔC is comprised of full-length Ku70 and Ku80ΔC lacking the C-terminal residues 591–732. His-tagged WRN-Exo was expressed from pET16b-WRN-Exo in BL21(DE3) (pLysS) by induction with 1 mM IPTG in 0.5 l cultures in LB containing 50 μg ml^−1^ ampicillin, and 30 μg ml^−1^ chloramphenicol for 16 h at 16 °C. Harvested cells were frozen and subsequently lysed in 20 mM Tris-HCl pH 7.5, 0.5 M NaCl, 5% glycerol, 1.4 mM β-mercaptoethanol, 1% Triton X-100, 1 mM phenylmethylsulphonyl fluoride, 10 mM imidazole, pH 8.0. Cells were sonicated (3 × 20 s) and the cell extracts clarified by centrifugation 12,000*g* for 30 min. The supernatant was incubated with 0.5 ml pre-washed Ni-agarose beads (Qiagen) for 20 min at 4 °C and the beads washed twice with 10 ml wash buffer (lysis buffer lacking detergent) before being transferred to a gravity-flow column. The resin was washed with a further 10 ml wash buffer containing 50 mM imidazole and proteins then eluted with wash buffer containing 250 mM imidazole. Fractions containing WRN-Exo were pooled and purified further by gel filtration using Superdex 200 equilibrated with 20 mM Tris-HCl pH 7.5, 0.3 M NaCl, 10% glycerol, 1 mM DTT. GST-tagged KBMs were expressed as above and purified using glutathione sepharose affinity chromatography.

### Fluorescence polarization assays

100 nM fluorescein-labeled peptides (Peptide Protein Research) were incubated at room temperature for 10 min with the indicated concentrations of Ku70/Ku80ΔC (KuΔC) in 20 mM HEPES pH 7.5, 200 mM NaCl, and 0.5 mM TCEP. Fluorescence polarization was measured in a POLARstar Omega microplate reader (BMG Labtech GmbH, Ortenberg, Germany). Fifty flashes were recorded for each well with an excitation wavelength of 485nm, and simultaneous detection of emission at 520 nm with parallel and perpendicular polarizers in-line. Background fluorescence in wells containing only buffer was subtracted from all values obtained for the samples. Polarization data were analysed using GraphPad Prism 5.0 by non-linear fitting with a one-site total binding model. All data represent the mean of at least three separate experiments and error bars represent 1 s.d. Peptide sequences are those depicted in Fig. 1a, in each case additionally preceded with fluorophore and four amino acid linker (Flu-GGYG). For competition assays, 1 μM KuΔC was incubated with 2.1 μM fluorescently labeled WRN-nA, WRN-cAX or MRI-A peptides for 10 min at room temperature followed by the indicated concentration of unlabeled APLF peptide (highest concentration; 29 μM).

### Laser microirradiation

2 × 10^5^ U2-OS cells or *Ku80*^*−/−*^ mouse embryonic fibroblasts^40^ were seeded in glass-bottomed 35-mm dishes (Mattek) in DMEM (+10% FCS) and 2 days later transfected with either (U2-OS cells) 1 μg plasmid DNA and 3 μl Genejuice (Merck Millipore) or (*Ku80*^*−/−*^ mouse embryonic fibroblasts) 7.5 μl GeneJuice and 0.5 μg of the indicated GFP-KBM plasmid, 1 μg of pmRFP-Ku70, and 1 μg of either pmRFP-C1 vector, pmRFP-Ku80 or pmRFP-Ku80^L68R^. 24 h after transfection, cells were pre-treated with 10 μg ml^−1^ Hoechst 34580 (Sigma) and micro-irradiated (210 nJ μm^−2^) with a 405 nm laser focused through a × 60 oil objective (Intelligent Imaging Innovations). Images were captured at 10 s intervals after treatment and image analysis was carried out using Slidebook software.

### GFP pull-down assays

HEK293T cells were cultured in DMEM supplemented with 10% FCS, glutamine and antibiotics in 15 cm culture dishes and at ∼70% confluence the media was replaced with 18 ml Hybridoma-SFM media (supplemented with 1% FCS and antibiotics) and supplemented with 2 ml transfection mix containing PEI (80 μg) and the appropriate plasmid (20 μg). Cells were harvested 48 h later, washed with cold PBS and flash frozen. Thawed pellets (5 × 10^6^ cells) were lysed in 400 μl lysis buffer (25 mM Tris-HCl pH 7.5, 150 mM NaCl, 10% glycerol, 0.5% Triton X-100, 1 mM DTT) containing protease and phosphatase inhibitors (Sigma) for 20 min at 4 °C. Samples were sonicated in water-bath sonicator for 10 min at 30 s intervals (30 s on/30 s off). Cell extracts were clarified by centrifugation (13,000*g*, 10 min, 4 °C) and 40 μl removed for the ‘input' sample. GFP-TRAP beads (20 μl; Chromotek) were washed three times with lysis buffer then incubated with the supernatant for 1 h at 4 °C. Unbound proteins were recovered by gentle centrifugation (2,700*g*, 2 min, 4 °C) and the beads were washed with 3 × 500 μl lysis buffer. Proteins were then eluted from the beads using SDS–PAGE loading buffer, heated at 95 °C for 5 min, and aliquots fractionated by 10% SDS–PAGE and transferred to Hybond-C membrane (GE Healthcare). Proteins were detected by immunoblotting using anti-GFP (Cell Signalling #2555S, 1/1,000 dilution), anti Ku80 (Abcam Ab80592, 1/10,000 dilution), anti DNA–PKcs (Abcam Ab80514, 1/1,000 dilution) or anti-RFP (Abcam, Ab62341, 1:1,000) antibodies. Pictures of the full membranes containing the blotted eluates from these experiments are shown in Supplementary Figs 6–8.

### Strep-tag pull-down assays

Streptavidin Mag Sepharose beads (100 μl; GE Healthcare) were washed three times with sample buffer (20 mM Hepes pH 7.5, 150 mM NaCl, 5% glycerol, 0.5 mM TCEP, 0.05% IGEPAL-CA640) then incubated with Strep-tagged 50 nM WRN, WRN^ΔcAX^ or WRN^W18G^ for 1 h at 4 °C. Unbound protein was recovered by applying magnetic force to the slurry, and the beads washed with 3 × 500 μl sample buffer. Recombinant untagged Ku protein (50 nM) was then incubated with the beads for 1 h at 4 °C, the unbound fraction removed and washes performed as above. Bound proteins were eluted from the beads using SDS–PAGE loading buffer, heated at 95 °C for 5 min, and aliquots fractionated by 10% SDS–PAGE and stained with Instant Blue (Expedeon).

### Exonuclease assays

A 5′ Cy3-labeled 30-bp oligonucleotide (5′ cy3- CCGTTTCGCTCAAGTTAGTATGTCAAAGCA -3′) was annealed to a complementary unlabeled 30-bp oligonucleotide (5′- CGTTGAAGCCTGCTTTGACATACTAACTTG -3′) to produce a 20-bp duplex with 10 nucleotide 5′ overhangs. 20 nM of DNA duplex was incubated at 37 °C for 30 min with 10 nM or the indicated titration of WRN-Exo or WRN-Exo^W18G^ in reaction buffer (20 mM Tris-HCl, pH 7.5, 50 mM KCl, 10 mM MgCl_2_, 1 mM DTT and 0.1 mg ml^−1^ BSA). Where indicated, reactions also contained 10 nM or the indicated titration of Ku, Ku70/Ku80ΔC or Ku70/Ku80ΔC^L68R^ and where indicated 5 mM of either MgCl_2_ or MnCl_2_. Reactions were stopped using 90% (v/v) Formamide/TBE and heated at 95 °C for 5 min. Products were fractionated on 16% TBE-Urea gels in 1 × TBE (89 mM Tris, 89 mM Boric Acid, 2 mM EDTA) and imaged on a Fuji imager using a Cy3 filter.

### γH2Ax assays

3 × 10^5^ of the indicated cells were seeded and grown on coverslips in 35-mm dishes and grown to confluence for 1 week. Cells were then treated with 2 Gy γIR, fixed with paraformaldehyde at the time-points indicated, and immunolabeled as previously described^41^. Cells were co-labeled with CENPF to confirm that cell populations were confluence-arrested.

## Additional information

**How to cite this article:** Grundy, G. J. *et al*. The Ku-binding motif is a conserved module for recruitment and stimulation of non-homologous end-joining proteins. *Nat. Commun.* 7:11242 doi: 10.1038/ncomms11242 (2016).

## Supplementary Material

Supplementary Information Supplementary Figures 1-8 and Supplementary Table 1

Supplementary Data 1 Data show putative KBM-containing human proteins identified by a Pattinprot database search employing the minimal KBM core consensus R-X-X-P-X-W. KBM's identified in APLF, WRN and MRI are highlighted in red
