# Supplementary material for: The Ku-binding motif is a conserved module for recruitment and stimulation of non-homologous end-joining proteins
Source: Nat Commun. 2016 Apr 11;7:11242. doi: 10.1038/ncomms11242 (PMC4831024; doi:10.1038/ncomms11242)
Supplement: Supplementary Information — Supplementary Figures 1-8 and Supplementary Table 1 [file ncomms11242-s1.pdf]

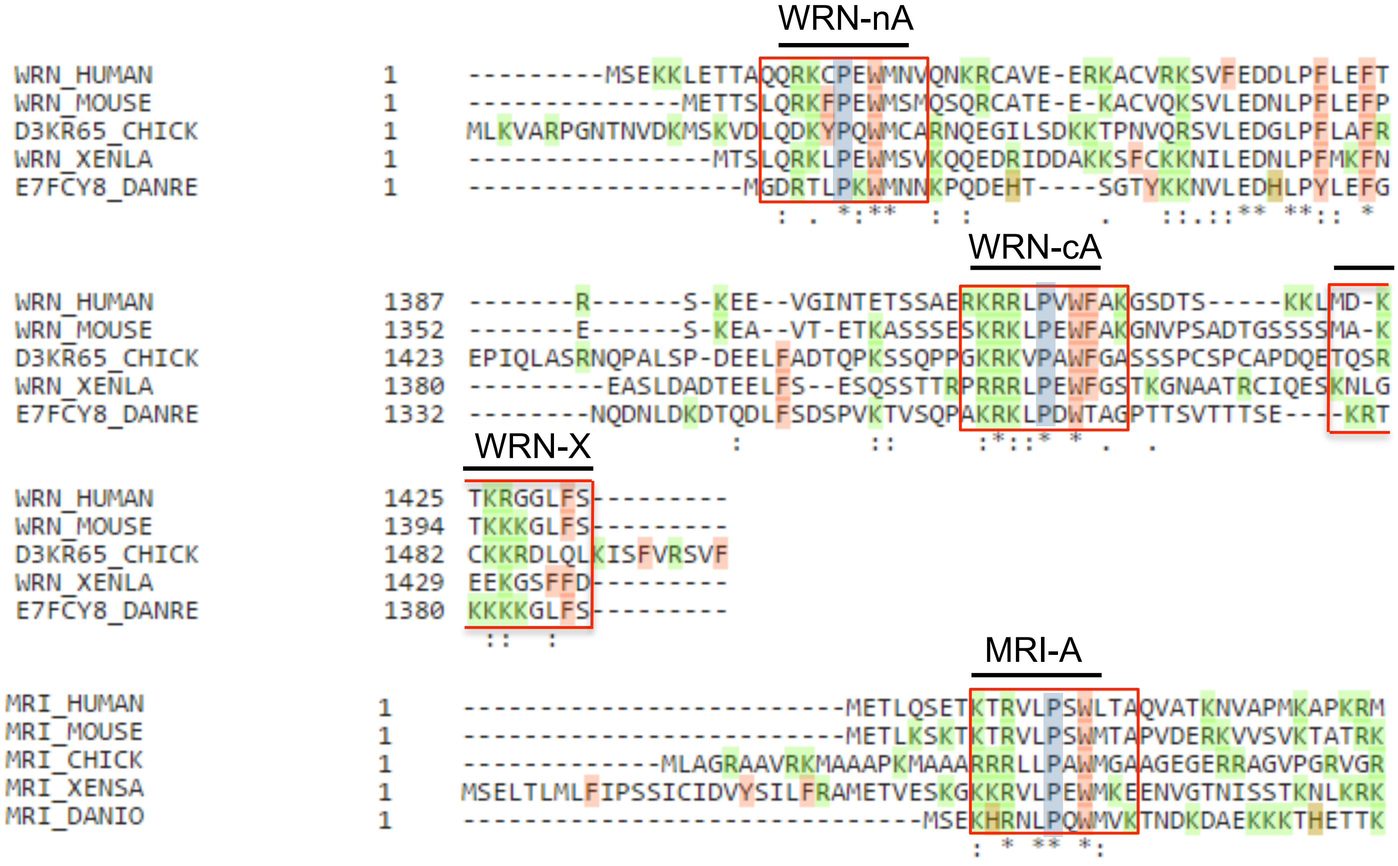

Supplementary Figure 1. Conservation of WRN & MRI KBMs in vertebrates. Alignment produced in Uniprot (uniprot.org/align) highlighting basic residues (green), aromatic residues (pink), invariant residues (*), and highly conserved residues with strongly (:) and weakly (.) similar properties. The conserved proline is highlighted in blue. Species: Human, *Homo sapiens*; Mouse, *Mus musculus*; Chicken, *Gallus gallus*; Frog, *Xenopus laevis*; Zebrafish, *Danio rerio*.

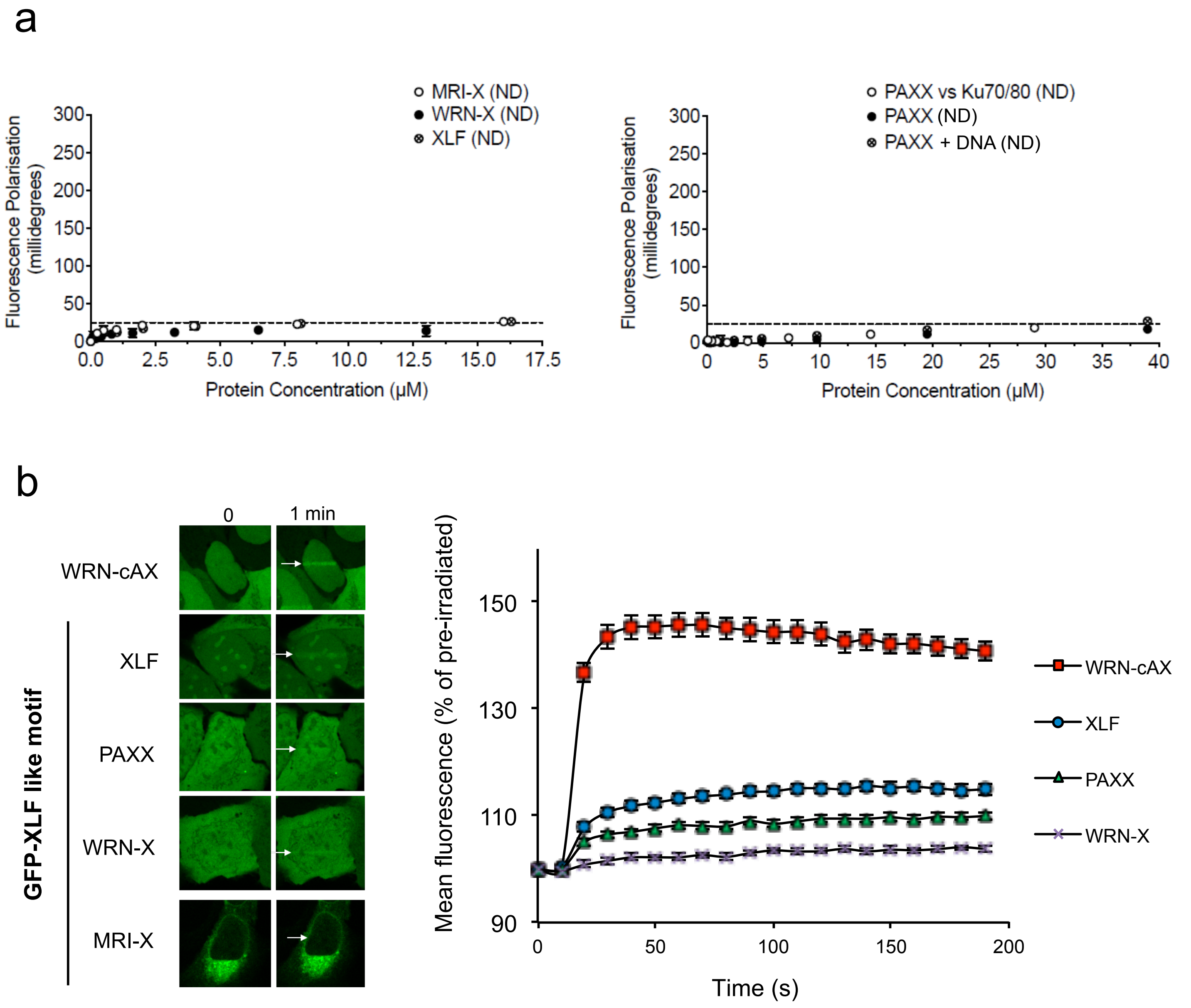

Supplementary Figure 2. XLF-like motifs; Interaction of with Ku and accumulation at sites of UVA laser-damage. (a), Fluorescence polarisation measuring direct interaction between synthetic fluorescein-labelled peptides encoding the indicated XLF-like motifs and Ku heterodimer (KuΔC). Note that for PAXX the XLF-like motif was also examined for interaction with full length heterodimer (Ku70/Ku80) and with KuΔC in the additional presence of DNA. Peptide sequences are those indicated in Fig.1a. All data points are the mean of three independent experiments (+/-SD). Interaction dissociation constants were all too weak to be determined ('ND'). **(b)**, U2-OS cells were transiently transfected with expression constructs encoding the indicated GFP-tagged XLF-like motifs or the WRN-cAX tandem peptide as a positive control and subjected to UVA laser microirradiation. The expressed KBM peptide sequences were; WRN-cAX (1399-1432), XLF (287-299), PAXX (190-204), WRN-X (1418-1432) and MRI-X (147-157). Images were captured immediately before and at 10s intervals following treatment. Representative images are shown on the left and quantified data on the right. GFP-tagged MRI-X peptide was not quantified because it was not present in the nucleus. Data are the mean GFP fluorescence (+/- s.e.m) in the laser track, relative to the fluorescence before irradiation (set at 100%), from 20 or more cells per experiment.

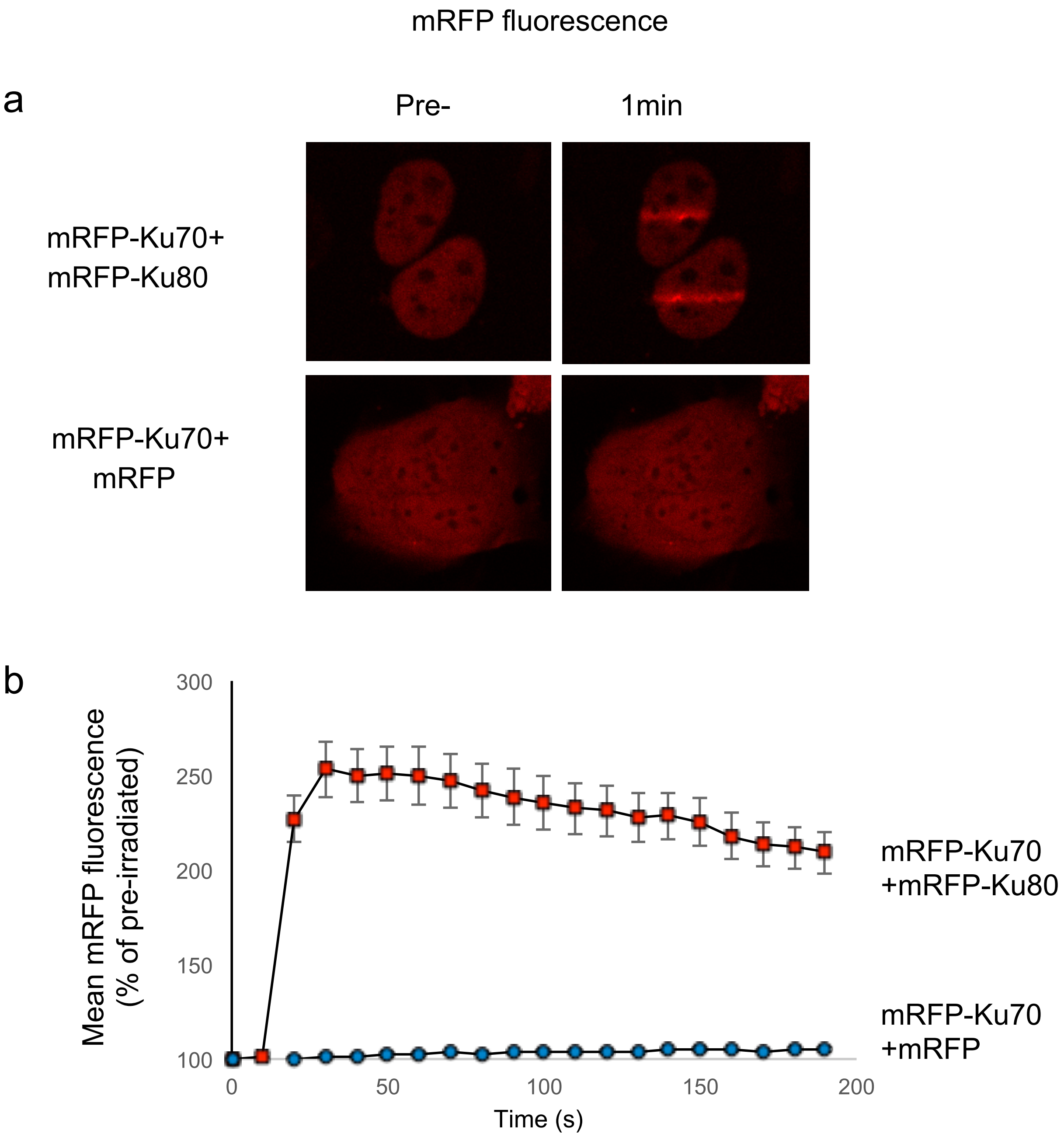

Supplementary Figure 3. Kinetics of Ku recruitment in MEFs. (a) $Ku80^{-/-}$ MEFs were co-transfected with mRFP-hKu70 and either mRFP-hKu80 (top) or mRFP-C1 vector (bottom). Cells were then subjected to UVA-laser micro-irradiation. (b) The mRFP signal was quantified at the site of DNA damage in cells transfected with the indicated expression constructs.

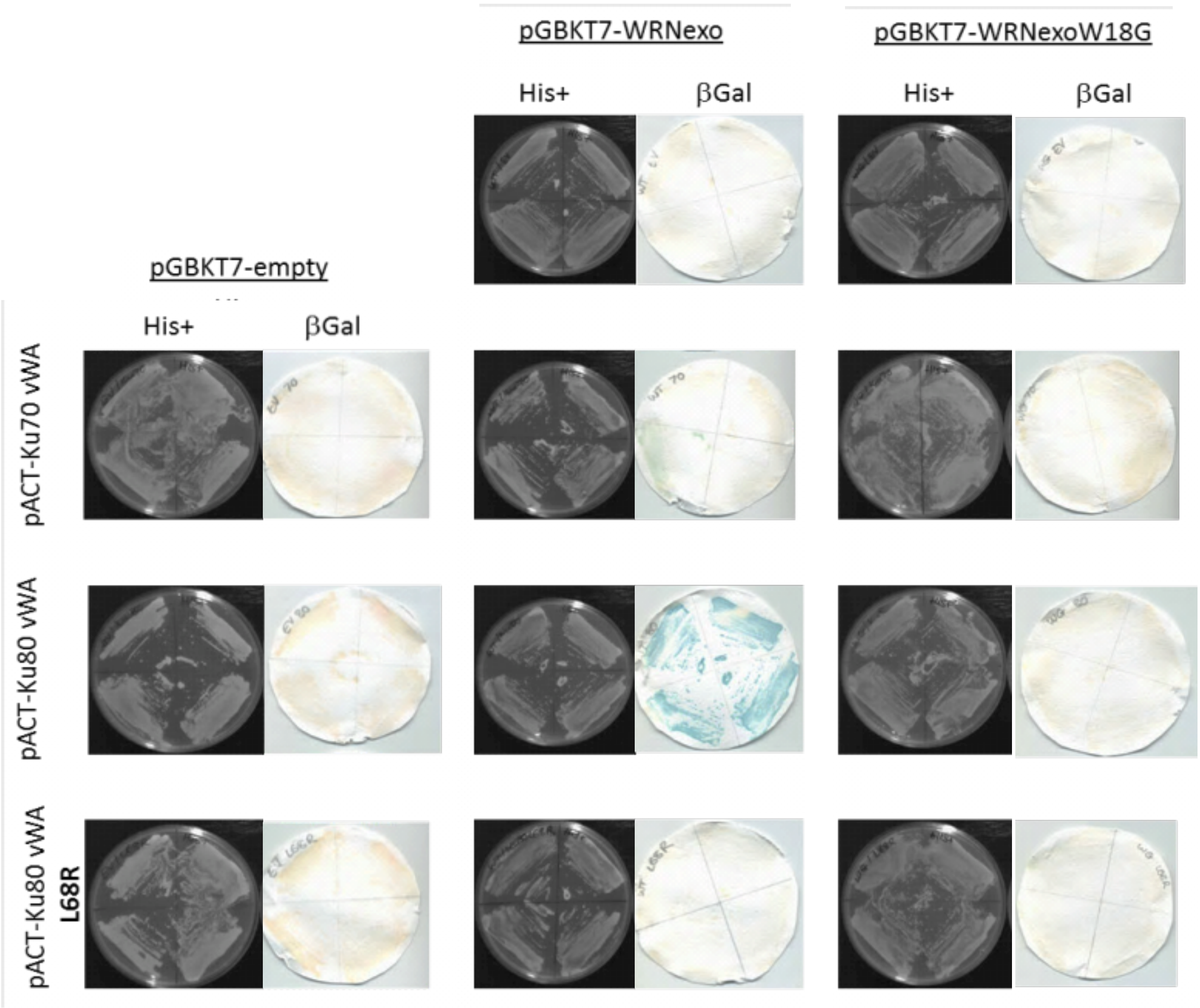

Supplementary Figure 4. Interaction of the WRN N-terminal KBM/exonuclease domain (WRN-Exo; residues 1-236) with the Ku80 vWA domain (residues 1-258) in yeast 2-hybrid assays. Yeast Y190 cells were transformed with empty pGBKT7, pGBKT7-WRNexo or pGBKT7-WRNexo^{W18G} and either empty pACT2, pACT2-Ku70vWA, pACT2-Ku80vWA, or pACT2-Ku80vWA^{L68R}. Transformed Y190 cells were selected on minimal media plates lacking leucine and tryptophan to select for bait and prey plasmids and interaction between the Ku80 vWA domain and WRNexo detected by β-galactosidase reporter gene expression (blue colourmetric assays) on filter-lifts.

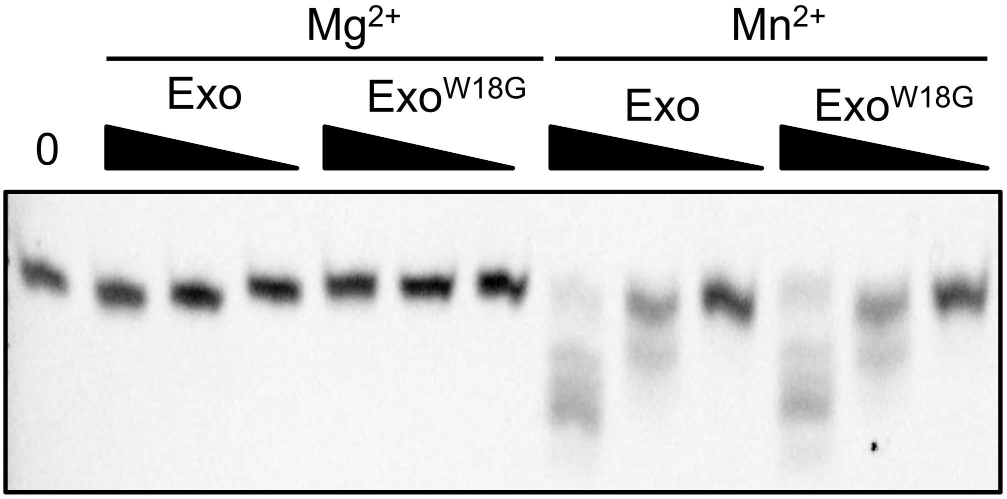

Supplementary Figure 5. Exonuclease assays were conducted as described in the main text using 100 nM, 20 nM or 5 nM of the indicated WRN nuclease in the absence of Ku and presence of either 5 mM Mn^{2+} or Mg^{2+} as indicated.

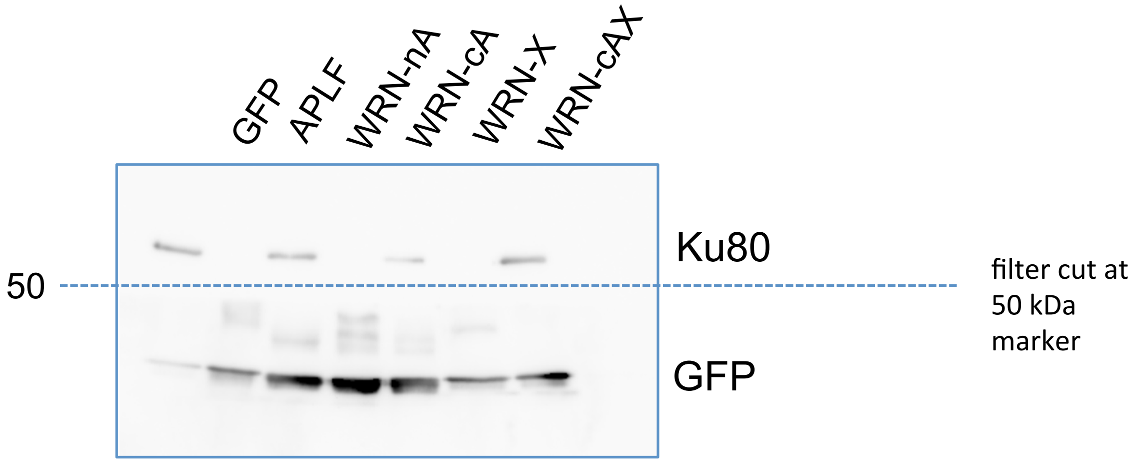

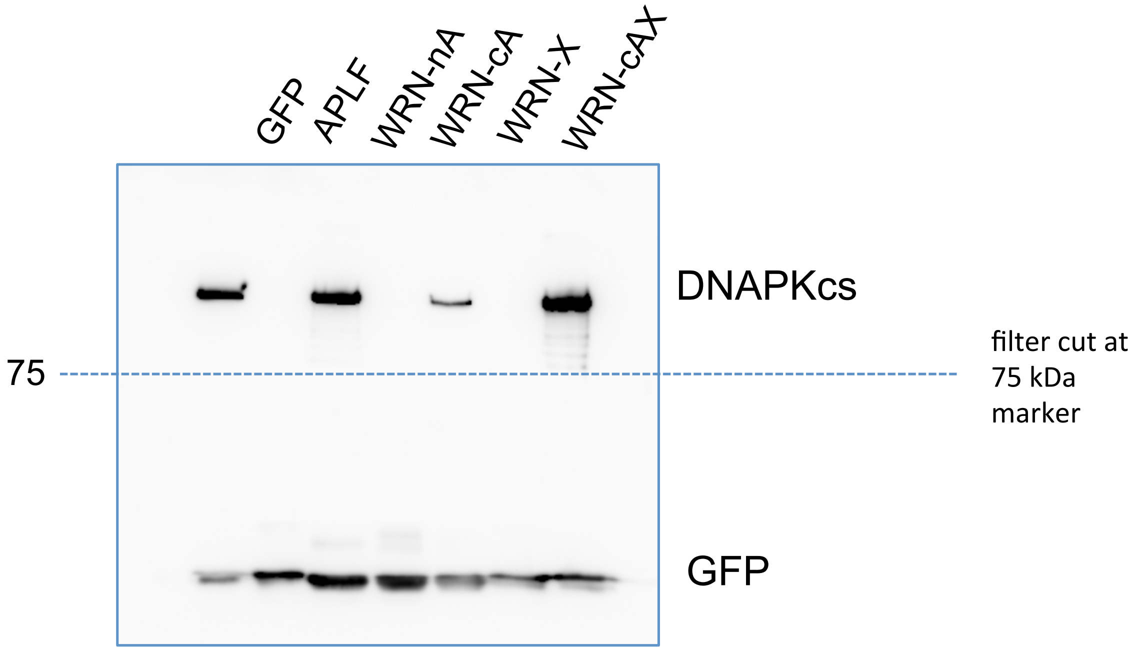

Supplementary Figure 6. Full membranes of the eluate blots employed in Fig.3a.

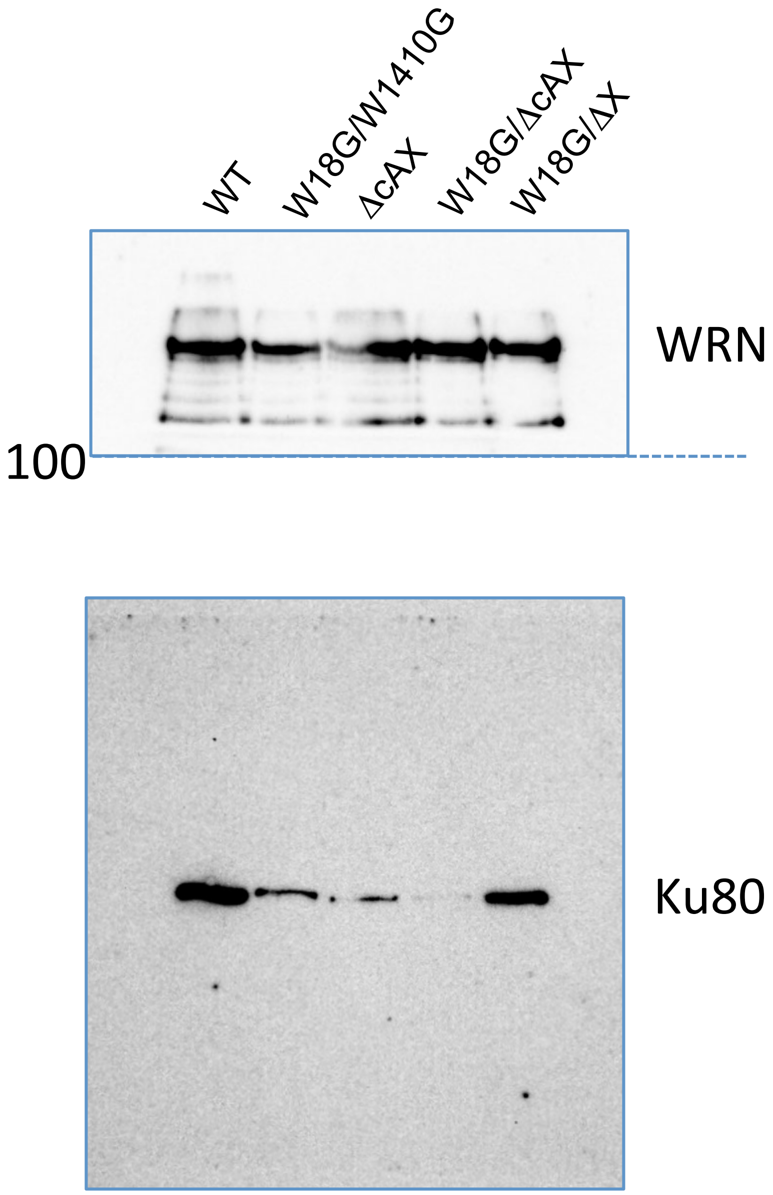

Supplementary Figure 7. Full membranes of the eluate blots employed in Fig.3d.

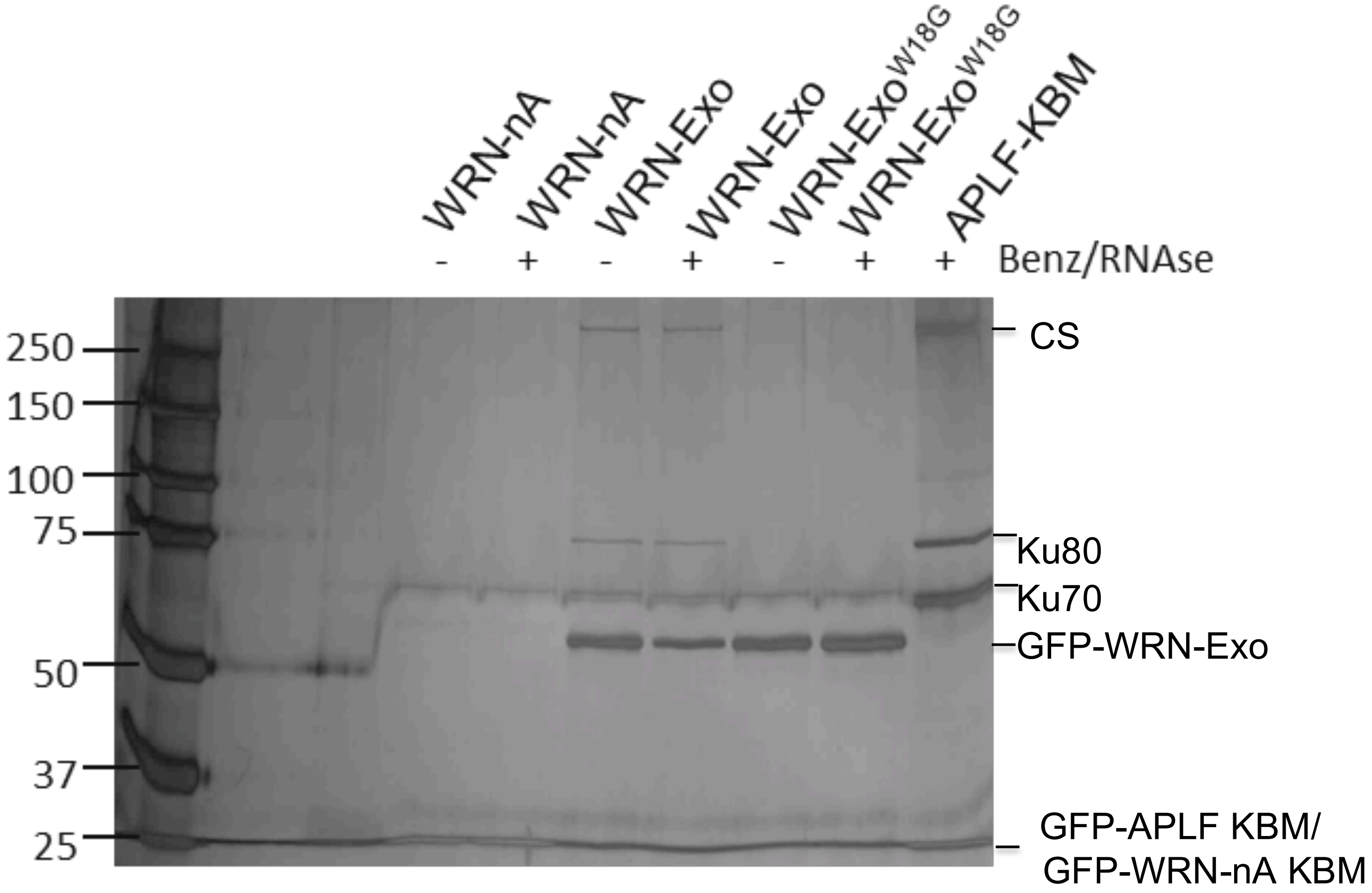

Supplementary Figure 8. Uncropped silver-stained gel employed in Fig.4b.

Supplementary Table

Plasmid	Primer 1 (5'-3')	Primer 2 (5'-3)	Method
pGEX6-XLF^{KBM}	XLF-T GATCCAAGGTCAAG AGGAAGAAGCCAAG GGGTCTCTTCAGTT AATCC	XLF-B TCGAGGATTAACTG AAGAGACCCCTTGG CTTCTTCCTCTTGAC CTTG	Annealed, ligated into pGEX6p1 BamHI/XhoI
pGEX6-PAXX^{KBM}	PAXX-T GATCTGGGTTCAAG AGTAAGAAACCAGC TGGTGGCGTGGACT TCGATGAGACCTAA C	PAXX-B TCGAGTTAGGTCTC ATCGAAGTCCACGC CACCAGCTGGTTTC TTACTCTTGAACCCA	Annealed, ligated into pGEX6p1 BamHI/XhoI
pGEX6-WRN-X	WRN-X-T GATCCAGCAAGAAA TTAATGGACAAAAC GAAAAGGGGAGGTC TTTTTAGTTAAC	WRN-X-B TCGAGTTAACTAAAA AGACCTCCCCTTTTC GTTTTGTCCATTAAT TTCTTGCTG	Annealed, ligated into pGEX6p1 BamHI/XhoI
pGEX-MRI-X	MRI-X-T GATCCGTGCTGAAA TACGTCCGGGAGAT CTTTTTCAGCTAGC	MRI-X-B TCGAGCTAGCTGAA AAAGATCTCCCGGA CGTATTTCAGCACG	Annealed, ligated into pGEX6p1 BamHI/XhoI
pGFP-APLF^{KBM}	APLF-T AATTCTGATGCCAATCC TTGCCGAGAGGAAAAG AATCCTTCCAACTTGGA TGTTAGCAGAACAG	APLF-B TCGACTGTTCTGCTAAC ATCCAAGTTGGAAGGA TTCTTTTCCTCTCGGCA AGGATTGGCATCAG	Annealed and ligated into peGFP-N1-EcoRI/SalI
pGFP-WRN-cAX	WRN-cAX-F GATCGAATTCTACTTCA TCTGCAGAGAGA AAG	WRN-cAX-R GATCGTCGACTTAACT AAAAAGACCTCCCCT	PCR digested EcoRI/SalI ligated into pEGFP-C1 EcoRI/SalI
pGFP-WRN-nA	WRN-N-T GATCGAATTCTGATGTT GGAAACAACTGCACAG CAGCGGAAATGTCCT GAATGGATGAATGTG CAGTCGACGATC	WRN-N-B GATCGTCGACTGCACA TTCATCCATTCAGGACA TTTCCGCTGCTGTGCA GTTGTTTCCAACATCAG AATTCGATC	Annealed, digested with EcoRI/SalI and ligated into peGFP-N1-EcoRI/SalI
pGFP-WRN-cA	WRN-C-T GATCGAATTCTGATGA CTTCATCTGCAGAGAG AAAGAGACGATTACCT GTGTGGTTTGCCAAAC AGTCGACGATC	WRN-C-B GATCGTCGACTGTTTG GCAAACCACACAGGTA ATCGTCTCTTTCTCTCT GCAGATGAAGTCATCA GAATTCGATC	Annealed, digested with EcoRI/SalI and ligated into peGFP-N1-EcoRI/SalI
pGFP-MRI-A	MRI-A-T GATCCGAGACTAAA ACGAGGGTCCTTCC CTCATGGCTGACAG CCTAGC	MRI-A-B TCGAGCTAGGCTGT CAGCCATGAGGGAA GGACCCTCGTTTTA GTCTCG	Annealed, ligated into pEGFP-C1 BglII/SalI
pGFP-XLF^{KBM}	XLF-T GATCCAAGGTCAAG AGGAAGAAGCCAAG	XLF-B TCGAGGATTAACTG AAGAGACCCCTTGG	Annealed, ligated into pEGFP-C1 BglII/SalI

	GGGTCTCTTCAGTTAATCC	CTTCTTCCTCTTGACCTTG	
pGFP-PAXX^{KBM}	PAXX-T<br>GATCTGGGTTCAAGAGTAAGAAACCAGCTGGTGGCGTGGACTTCGATGAGACCTAAC	PAXX-B<br>TCGAGTTAGGTCTCATCGAAGTCCACGCCACCAGCTGGTTTCTTACTCTTGAACCCA	Annealed, ligated into pEGFP-C1 BglII/SalI
pGFP-MRI-X	MRI-X-T<br>GATCCGTGCTGAAATACGTCCGGGAGATCTTTTTCAGCTAGC	MRI-X-B<br>TCGAGCTAGCTGAAAAAGATCTCCCGGACGTATTTCAGCACG	Annealed, ligated into pEGFP-C1 BglII/SalI
pGFP-WRN-X	WRN-X-T<br>GATCCAGCAAGAAATTAATGGACAAAACGAAAAGGGGAGGTCTTTTTAGTTAAC	WRN-X-B<br>TCGAGTTAACTAAAAAGACCTCCCCTTTTCGTTTTGTCCATTAATTTCTTGCTG	Annealed, ligated into pEGFP-C1 BglII/SalI
pGFP-XLF^{KBMF298A}	XLF-FA-T<br>GATCCAAGGTCAAGAGGAAGAAGCCAAGGGGTCTCGCCAGTTAAC	XLF-FA-B<br>TCGAGTTAACTGGCGAGACCCCTTGGCTTCTTCCTCTTGACCTTG	Annealed, ligated into pEGFP-C1 BglII/SalI
pGFP-WRN	WRNstop3f<br>CGAAAAGGGGAGGTCTTTTTAGTTAACCGGGATCCACCGGATC	WRNstop3r<br>GATCCGGTGGATCCCGGTTAACTAAAAAGACCTCCCCTTTTCG	SDM using template pEGFP-C3-WRN (ref Bohr)
pGFP-WRNΔX	WRNstop2f<br>GCCAAAGGAAGTGATACCTGAAAGAAATTAATGGACAAAACG	WRNstop2r<br>CGTTTTGTCCATTAATTTCTTTCAGGTATCACTTCCTTTGGC	SDM using template pEGFP-C3-WRN (ref Bohr)
pGFP-WRNΔcAX	WRNstop1f<br>GCATCAATACTGAGACTTAATCTGCAGAGAGAAAGAG	WRNstop1r<br>CTCTTTCTCTCTGCAGATTAAGTCTCAGTATTGATGC	SDM using template pEGFP-C3-WRN (ref Bohr)
pGFP-WRN ^{W18G}	W18GF<br>CGGAAATGTCCTGAAGGGATGAATGTGCAG	W18GR<br>CTGCACATTCATCCCTTCAGGACATTTCCG	SDM using template pGFP-WRN
pGFP-WRN ^{W1410G}	W1410G-F<br>GAGACGATTACCTGTGGGGTTTGCCAAAG	W1410G-R<br>CTTTGGCAAACCCCACAGGTAATCGTCTC	SDM using template pGFP-WRN
pGFP-WRN $^{W18G/W1410G}$	W18GF<br>CGGAAATGTCCTGAAGGGATGAATGTGCAG	W18GR<br>CTGCACATTCATCCCTTCAGGACATTTCCG	SDM using template pGFP-WRN ^{W1410G}
pGFPΔX^{W18G}	WRNstop2f<br>GCCAAAGGAAGTGATACCTGAAAGAAATTAATGGACAAAACG	WRNstop2r<br>CGTTTTGTCCATTAATTTCTTTCAGGTATCACTTCCTTTGGC	SDM using template pGFP-WRN ^{W18G}
pGFP-WRN-cAX^{F1431A}	WRN-F<br>TACTACGGATCCTCTGCAGAGAGAAAGAGACGATTACC	CX-FA rev<br>TACTACCTCGAGTTAACTAGCAAGACCTCCCCTTTTCG	PCR product digested BamHI/XhoI ligated into pEGFP-C1 BglII/SalI
pGFP-WRN-cAX^{W1410G}	WRN-F<br>TACTACGGATCCTCTGCAGAGAGAAAGAG	WRN-R<br>TACTACCCTCGAGTTAACTAAAAAGACCTC	PCR product using pGFP-WRN^{W1410G} template digested

	ACGATTACC	CCCTTTTCG	BamHI/XhoI ligated into pEGFP-C1 BglII/SalI
pET16b-WRN-Exo	ExoF<br>GATATACCATGGGC<br>ATGAGTGAAAAAAAA<br>TTGGAAACAACTG	ExoR<br>GATCTACTCGAGTTA<br>ATGATGATGATGAT<br>GATGATGATGCCTTT<br>GCACAGTATCATCC<br>AAAAT	PCR product from template pEGFP-C3-WRN ligated into pET16b (NcoI/XhoI)
pET16b-WRN-Exo^{W18G}	W18G-F<br>CGGAAATGTCCTGA<br>AGGGATGAATGTGC<br>AG	W18G-R<br>CTGCACATTCATCC<br>CTTCAGGACATTTCC<br>G	SDM using template pET16b-WRN-Exo
pGEX6-WRN-Exo-cAX	BglII-ExoF<br>TAGTATAGATCTATG<br>GGCATGAGTGAAAA<br>AAAATTGGAAACAAC<br>TG	BglII-ExoR<br>CATGCAAGATCTATGA<br>TGATGATGATGATGAT<br>GATGCCTTTG	BglII digested PCR product using pET16b-WRN-Exo template, ligated into BamHI site of pGEX6-WRN-cAX
pGEX6-WRN-Exo^{W18G}-cAX	BglII-ExoF<br>TAGTATAGATCTATG<br>GGCATGAGTGAAAA<br>AAAATTGGAAACAAC<br>TG	BglII-ExoR<br>CATGCAAGATCTATGA<br>TGATGATGATGATGAT<br>GATGCCTTTG	BglII digested PCR product using pET16b-WRN-Exo^{W18G} template, ligated into BamHI site of pGEX6-WRN-cAX
pGFP-WRN-Exo	BglII-ExoF<br>TAGTATAGATCTATG<br>GGCATGAGTGAAAA<br>AAAATTGGAAACAAC<br>TG	ExoR<br>GATCTACTCGAGTTA<br>ATGATGATGATGAT<br>GATGATGATGCCTTT<br>GCACAGTATCATCC<br>AAAAT	PCR product using pGEX6-WRN-Exo template, ligated into pEGFP-C1 BglII/SalI sites
pGFP-WRN-Exo^{W18G}	BglII-ExoF<br>TAGTATAGATCTATG<br>GGCATGAGTGAAAA<br>AAAATTGGAAACAAC<br>TG	ExoR<br>GATCTACTCGAGTTA<br>ATGATGATGATGAT<br>GATGATGATGCCTTT<br>GCACAGTATCATCC<br>AAAAT	PCR product using pGEX6-WRN-Exo^{W18G} template, ligated into pEGFP-C1 BglII/SalI sites
pGFP-WRN-Exo-cAX	BglII-ExoF<br>TAGTATAGATCTATG<br>GGCATGAGTGAAAA<br>AAAATTGGAAACAAC<br>TG	WRN-R<br>TACTACCCTCGAGTT<br>AACTAAAAAGACCTC<br>CCCTTTTCG	PCR product using pGEX6-WRN-Exo-CX template, ligated into pEGFP-C1 BglII/SalI sites
pLXSN-WRN^{W18G}	W18GF<br>CGGAAATGTCCTGA<br>AGGGATGAATGTGC<br>AG	W18GR<br>CTGCACATTCATCC<br>CTTCAGGACATTTCC<br>G	SDM using template pLXSN-WRN
pLXSN-WRN^{W1410G}	W1410G-F<br>GAGACGATTACCTG<br>TGGGGTTTGCCAAA<br>G	W1410G-R<br>CTTTGGCAAACCCC<br>ACAGGTAATCGTCT<br>C	SDM using template pLXSN-WRN
