# Supplementary material for: The Ku-binding motif is a conserved module for recruitment and stimulation of non-homologous end-joining proteins
Source: Nat Commun. 2016 Apr 11;7:11242. doi: 10.1038/ncomms11242 (PMC4831024; doi:10.1038/ncomms11242)
Supplement: Supplementary Data 1 — Data show putative KBM-containing human proteins identified by a Pattinprot database search employing the minimal KBM core consensus R-X-X-P-X-W. KBM's identified in APLF, WRN and MRI are highlighted in red [file ncomms11242-s2.docx]

Supplementary Data 1

[NPSA](https://npsa-prabi.ibcp.fr/cgi-bin/seq_methonseq.pl?seqid=A0A087X1C5&db=unipsp&process=5be3cc137c874192&seqtype=1) [gnl|unipsp|A0A087X1C5](http://www.uniprot.org/uniprot/A0A087X1C5) [Eukaryota] Putative cytochrome P450 2D7 [Metazoa][Homo sapiens] (length=515 residues).

Site : 123- 128, Identity

gprsqgvils_RYGPAW_reqrrfsvst

--------------------------------------------------------------------------------

[NPSA](https://npsa-prabi.ibcp.fr/cgi-bin/seq_methonseq.pl?seqid=A4GXA9&db=unipsp&process=5be3cc137c874193&seqtype=1) [gnl|unipsp|A4GXA9](http://www.uniprot.org/uniprot/A4GXA9) [Eukaryota] Probable crossover junction endonuclease EME2 [Metazoa][Homo sapiens] (length=379 residues).

Site : 26- 31, Identity

rgrgrggsgq_RRPPTW_eisdsdaeds

--------------------------------------------------------------------------------

[NPSA](https://npsa-prabi.ibcp.fr/cgi-bin/seq_methonseq.pl?seqid=A5PL33&db=unipsp&process=5be3cc137c874194&seqtype=1) [gnl|unipsp|A5PL33](http://www.uniprot.org/uniprot/A5PL33) [Eukaryota] Protein KRBA1 [Metazoa][Homo sapiens] (length=1030 residues).

Site : 870- 875, Identity

gprwahgpgh_RHLPYW_rqkgptrpkp

--------------------------------------------------------------------------------

[NPSA](https://npsa-prabi.ibcp.fr/cgi-bin/seq_methonseq.pl?seqid=A6NC42&db=unipsp&process=5be3cc137c874195&seqtype=1) [gnl|unipsp|A6NC42](http://www.uniprot.org/uniprot/A6NC42) [Eukaryota] Developmental pluripotency-associated 5 protein [Metazoa][Homo sapiens] (length=116 residues).

Site : 8- 13, Identity

‹mgtlpar_RHIPPW_vkvpedlkdp

--------------------------------------------------------------------------------

[NPSA](https://npsa-prabi.ibcp.fr/cgi-bin/seq_methonseq.pl?seqid=A6NEC2&db=unipsp&process=5be3cc137c874196&seqtype=1) [gnl|unipsp|A6NEC2](http://www.uniprot.org/uniprot/A6NEC2) [Eukaryota] Puromycin-sensitive aminopeptidase-like protein [Metazoa][Homo sapiens] (length=478 residues).

Site : 186- 191, Identity

vtqfeatdar_RAFPCW_deraikatfd

--------------------------------------------------------------------------------

[NPSA](https://npsa-prabi.ibcp.fr/cgi-bin/seq_methonseq.pl?seqid=A6NED2&db=unipsp&process=5be3cc137c874197&seqtype=1) [gnl|unipsp|A6NED2](http://www.uniprot.org/uniprot/A6NED2) [Eukaryota] RCC1 domain-containing protein 1 [Metazoa][Homo sapiens] (length=376 residues).

Site : 106- 111, Identity

lqvwaaesal_RGEPLW_aqnvvpeaeg

--------------------------------------------------------------------------------

[NPSA](https://npsa-prabi.ibcp.fr/cgi-bin/seq_methonseq.pl?seqid=A6NFA1&db=unipsp&process=5be3cc137c874198&seqtype=1) [gnl|unipsp|A6NFA1](http://www.uniprot.org/uniprot/A6NFA1) [Eukaryota] Metalloprotease TIKI2 [Metazoa][Homo sapiens] (length=517 residues).

Site : 160- 165, Identity

lfnaiagnwe_RKRPVW_vmlmvnslte

--------------------------------------------------------------------------------

[NPSA](https://npsa-prabi.ibcp.fr/cgi-bin/seq_methonseq.pl?seqid=A6NFR6&db=unipsp&process=5be3cc137c874199&seqtype=1) [gnl|unipsp|A6NFR6](http://www.uniprot.org/uniprot/A6NFR6) [Eukaryota] Putative uncharacterized protein C5orf60 [Metazoa][Homo sapiens] (length=353 residues).

Site : 260- 265, Identity

hkprgrslpr_RRNPGW_vswsdsmqad

--------------------------------------------------------------------------------

[NPSA](https://npsa-prabi.ibcp.fr/cgi-bin/seq_methonseq.pl?seqid=A6NGQ2&db=unipsp&process=5be3cc137c874200&seqtype=1) [gnl|unipsp|A6NGQ2](http://www.uniprot.org/uniprot/A6NGQ2) [Eukaryota] Oocyte-expressed protein homolog [Metazoa][Homo sapiens] (length=149 residues).

Site : 35- 40, Identity

rrlplpppqi_RIRPWW_fpvqelrdpl

--------------------------------------------------------------------------------

[NPSA](https://npsa-prabi.ibcp.fr/cgi-bin/seq_methonseq.pl?seqid=A6NGW2&db=unipsp&process=5be3cc137c874201&seqtype=1) [gnl|unipsp|A6NGW2](http://www.uniprot.org/uniprot/A6NGW2) [Eukaryota] Putative stereocilin-like protein [Metazoa][Homo sapiens] (length=1772 residues).

Site : 101- 106, Identity

lrlhdflvtl_RGSPDW_epmlgllgdm

--------------------------------------------------------------------------------

[NPSA](https://npsa-prabi.ibcp.fr/cgi-bin/seq_methonseq.pl?seqid=A6NKP2&db=unipsp&process=5be3cc137c874202&seqtype=1) [gnl|unipsp|A6NKP2](http://www.uniprot.org/uniprot/A6NKP2) [Eukaryota] Putative short-chain dehydrogenase/reductase family 42E member 2 [Metazoa][Homo sapiens] (length=422 residues).

Site : 65- 70, Identity

ksgtsvilld_RRRPQW_elspetkfiq

--------------------------------------------------------------------------------

[NPSA](https://npsa-prabi.ibcp.fr/cgi-bin/seq_methonseq.pl?seqid=A6NMN3&db=unipsp&process=5be3cc137c874203&seqtype=1) [gnl|unipsp|A6NMN3](http://www.uniprot.org/uniprot/A6NMN3) [Eukaryota] Putative protein FAM170B [Metazoa][Homo sapiens] (length=283 residues).

Site : 191- 196, Identity

dllecclqel_REPPDW_lvttnygvrc

--------------------------------------------------------------------------------

[NPSA](https://npsa-prabi.ibcp.fr/cgi-bin/seq_methonseq.pl?seqid=A6NNL5&db=unipsp&process=5be3cc137c874204&seqtype=1) [gnl|unipsp|A6NNL5](http://www.uniprot.org/uniprot/A6NNL5) [Eukaryota] Uncharacterized protein C15orf61 [Metazoa][Homo sapiens] (length=157 residues).

Site : 43- 48, Identity

sevltrhllq_RRLPHW_tsfcvpysav

--------------------------------------------------------------------------------

[NPSA](https://npsa-prabi.ibcp.fr/cgi-bin/seq_methonseq.pl?seqid=A7KAX9&db=unipsp&process=5be3cc137c874205&seqtype=1) [gnl|unipsp|A7KAX9](http://www.uniprot.org/uniprot/A7KAX9) [Eukaryota] Rho GTPase-activating protein 32 [Metazoa][Homo sapiens] (length=2087 residues).

Site : 63- 68, Identity

vpelhrnvhp_RERPDW_eetlsamarg

--------------------------------------------------------------------------------

[NPSA](https://npsa-prabi.ibcp.fr/cgi-bin/seq_methonseq.pl?seqid=A8K5M9&db=unipsp&process=5be3cc137c874206&seqtype=1) [gnl|unipsp|A8K5M9](http://www.uniprot.org/uniprot/A8K5M9) [Eukaryota] Uncharacterized protein C15orf62, mitochondrial [Metazoa][Homo sapiens] (length=175 residues).

Site : 62- 67, Identity

irelqgrpdg_RRLPLW_gdeqpratll

--------------------------------------------------------------------------------

[NPSA](https://npsa-prabi.ibcp.fr/cgi-bin/seq_methonseq.pl?seqid=A8MVM7&db=unipsp&process=5be3cc137c874207&seqtype=1) [gnl|unipsp|A8MVM7](http://www.uniprot.org/uniprot/A8MVM7) [Eukaryota] Putative uncharacterized protein ENSP00000382790 [Metazoa][Homo sapiens] (length=634 residues).

Site : 532- 537, Identity

sfevlsnlnk_RKRPPW_kitemstkrh

--------------------------------------------------------------------------------

[NPSA](https://npsa-prabi.ibcp.fr/cgi-bin/seq_methonseq.pl?seqid=A8MWP6&db=unipsp&process=5be3cc137c874208&seqtype=1) [gnl|unipsp|A8MWP6](http://www.uniprot.org/uniprot/A8MWP6) [Eukaryota] Uncharacterized protein ENSP00000382042 [Metazoa][Homo sapiens] (length=167 residues).

Site : 132- 137, Identity

ypnllcdfgp_RQGPLW_alllekrqma

--------------------------------------------------------------------------------

[NPSA](https://npsa-prabi.ibcp.fr/cgi-bin/seq_methonseq.pl?seqid=A8MXK9&db=unipsp&process=5be3cc137c874209&seqtype=1) [gnl|unipsp|A8MXK9](http://www.uniprot.org/uniprot/A8MXK9) [Eukaryota] Uncharacterized protein ENSP00000382033 [Metazoa][Homo sapiens] (length=166 residues).

Site : 131- 136, Identity

ypnllcdfgp_RQGPLW_alllekrqma

--------------------------------------------------------------------------------

[NPSA](https://npsa-prabi.ibcp.fr/cgi-bin/seq_methonseq.pl?seqid=B1AK53&db=unipsp&process=5be3cc137c874210&seqtype=1) [gnl|unipsp|B1AK53](http://www.uniprot.org/uniprot/B1AK53) [Eukaryota] Espin [Metazoa][Homo sapiens] (length=854 residues).

Site : 748- 753, Identity

alipthdeqg_RPIPEW_krqvmvrkmq

--------------------------------------------------------------------------------

[NPSA](https://npsa-prabi.ibcp.fr/cgi-bin/seq_methonseq.pl?seqid=C9J069&db=unipsp&process=5be3cc137c874211&seqtype=1) [gnl|unipsp|C9J069](http://www.uniprot.org/uniprot/C9J069) [Eukaryota] Uncharacterized protein C9orf172 [Metazoa][Homo sapiens] (length=976 residues).

Site : 396- 401, Identity

dvlartyphp_RSSPAW_adwgprpyrt

--------------------------------------------------------------------------------

[NPSA](https://npsa-prabi.ibcp.fr/cgi-bin/seq_methonseq.pl?seqid=C9J3V5&db=unipsp&process=5be3cc137c874212&seqtype=1) [gnl|unipsp|C9J3V5](http://www.uniprot.org/uniprot/C9J3V5) [Eukaryota] Testis-expressed sequence 22 protein [Metazoa][Homo sapiens] (length=150 residues).

Site : 132- 137, Identity

stnafqafla_RSAPFW_hnatfeasrs

--------------------------------------------------------------------------------

[NPSA](https://npsa-prabi.ibcp.fr/cgi-bin/seq_methonseq.pl?seqid=C9JH25&db=unipsp&process=5be3cc137c874213&seqtype=1) [gnl|unipsp|C9JH25](http://www.uniprot.org/uniprot/C9JH25) [Eukaryota] Proline-rich transmembrane protein 4 [Metazoa][Homo sapiens] (length=899 residues).

Site : 94- 99, Identity

epgeevasgl_RTDPLW_ellvgssgns

--------------------------------------------------------------------------------

[NPSA](https://npsa-prabi.ibcp.fr/cgi-bin/seq_methonseq.pl?seqid=D6REC4&db=unipsp&process=5be3cc137c874214&seqtype=1) [gnl|unipsp|D6REC4](http://www.uniprot.org/uniprot/D6REC4) [Eukaryota] Cilia- and flagella-associated protein 99 [Metazoa][Homo sapiens] (length=459 residues).

Site : 353- 358, Identity

saprtarpkp_RVSPDW_weepgrlkag

--------------------------------------------------------------------------------

[NPSA](https://npsa-prabi.ibcp.fr/cgi-bin/seq_methonseq.pl?seqid=E7ERA6&db=unipsp&process=5be3cc137c874215&seqtype=1) [gnl|unipsp|E7ERA6](http://www.uniprot.org/uniprot/E7ERA6) [Eukaryota] RING finger protein 223 [Metazoa][Homo sapiens] (length=249 residues).

Site : 131- 136, Identity

lqarmpahlr_REEPVW_legtklccqp

--------------------------------------------------------------------------------

[NPSA](https://npsa-prabi.ibcp.fr/cgi-bin/seq_methonseq.pl?seqid=H3BR10&db=unipsp&process=5be3cc137c874216&seqtype=1) [gnl|unipsp|H3BR10](http://www.uniprot.org/uniprot/H3BR10) [Eukaryota] Small leucine-rich protein 1 [Metazoa][Homo sapiens] (length=107 residues).

Site : 47- 52, Identity

amssvlsafm_RELPGW_flffgvflpv

--------------------------------------------------------------------------------

[NPSA](https://npsa-prabi.ibcp.fr/cgi-bin/seq_methonseq.pl?seqid=H3BV60&db=unipsp&process=5be3cc137c874217&seqtype=1) [gnl|unipsp|H3BV60](http://www.uniprot.org/uniprot/H3BV60) [Eukaryota] Transforming growth factor-beta receptor type 3-like protein [Metazoa][Homo sapiens] (length=316 residues).

Site : 97- 102, Identity

srvfvqaala_RPSPRW_glalhrcsvt

--------------------------------------------------------------------------------

[NPSA](https://npsa-prabi.ibcp.fr/cgi-bin/seq_methonseq.pl?seqid=I3L1E1&db=unipsp&process=5be3cc137c874218&seqtype=1) [gnl|unipsp|I3L1E1](http://www.uniprot.org/uniprot/I3L1E1) [Eukaryota] Uncharacterized protein C19orf84 [Metazoa][Homo sapiens] (length=186 residues).

Site : 112- 117, Identity

pprgrggwev_RHRPGW_grglhrrglg

--------------------------------------------------------------------------------

[NPSA](https://npsa-prabi.ibcp.fr/cgi-bin/seq_methonseq.pl?seqid=O00192&db=unipsp&process=5be3cc137c874219&seqtype=1) [gnl|unipsp|O00192](http://www.uniprot.org/uniprot/O00192) [Eukaryota] Armadillo repeat protein deleted in velo-cardio-facial syndrome [Metazoa][Homo sapiens] (length=962 residues).

Site : 350- 355, Identity

vrrspsvdsa_RKEPRW_rdpelpevla

--------------------------------------------------------------------------------

[NPSA](https://npsa-prabi.ibcp.fr/cgi-bin/seq_methonseq.pl?seqid=O00204&db=unipsp&process=5be3cc137c874220&seqtype=1) [gnl|unipsp|O00204](http://www.uniprot.org/uniprot/O00204) [Eukaryota] Sulfotransferase family cytosolic 2B member 1 [Metazoa][Homo sapiens] (length=365 residues).

Site : 93- 98, Identity

ilkegdpswi_RSVPIW_erapwcetiv

--------------------------------------------------------------------------------

[NPSA](https://npsa-prabi.ibcp.fr/cgi-bin/seq_methonseq.pl?seqid=O00476&db=unipsp&process=5be3cc137c874221&seqtype=1) [gnl|unipsp|O00476](http://www.uniprot.org/uniprot/O00476) [Eukaryota] Sodium-dependent phosphate transport protein 4 [Metazoa][Homo sapiens] (length=420 residues).

Site : 214- 219, Identity

kqplpikaml_RSLPIW_siclgcfshq

--------------------------------------------------------------------------------

[NPSA](https://npsa-prabi.ibcp.fr/cgi-bin/seq_methonseq.pl?seqid=O14521&db=unipsp&process=5be3cc137c874222&seqtype=1) [gnl|unipsp|O14521](http://www.uniprot.org/uniprot/O14521) [Eukaryota] Succinate dehydrogenase [ubiquinone] cytochrome b small subunit, mitochondrial [Metazoa][Homo sapiens] (length=159 residues).

Site : 38- 43, Identity

pahisaflqd_RPIPEW_cgvqhihlsp

--------------------------------------------------------------------------------

[NPSA](https://npsa-prabi.ibcp.fr/cgi-bin/seq_methonseq.pl?seqid=O14662&db=unipsp&process=5be3cc137c874223&seqtype=1) [gnl|unipsp|O14662](http://www.uniprot.org/uniprot/O14662) [Eukaryota] Syntaxin-16 [Metazoa][Homo sapiens] (length=325 residues).

Site : 73- 78, Identity

dpeaaigvtk_RPPPKW_vdgvdeiqyd

--------------------------------------------------------------------------------

[NPSA](https://npsa-prabi.ibcp.fr/cgi-bin/seq_methonseq.pl?seqid=O14976&db=unipsp&process=5be3cc137c874224&seqtype=1) [gnl|unipsp|O14976](http://www.uniprot.org/uniprot/O14976) [Eukaryota] Cyclin-G-associated kinase [Metazoa][Homo sapiens] (length=1311 residues).

Site : 717- 722, Identity

veveprdrps_REAPPW_enssmrglnp

--------------------------------------------------------------------------------

[NPSA](https://npsa-prabi.ibcp.fr/cgi-bin/seq_methonseq.pl?seqid=O15015&db=unipsp&process=5be3cc137c874225&seqtype=1) [gnl|unipsp|O15015](http://www.uniprot.org/uniprot/O15015) [Eukaryota] Zinc finger protein 646 [Metazoa][Homo sapiens] (length=1829 residues).

Site : 320- 325, Identity

lehqqshege_RQEPRW_eekgmpttng

--------------------------------------------------------------------------------

[NPSA](https://npsa-prabi.ibcp.fr/cgi-bin/seq_methonseq.pl?seqid=O15259&db=unipsp&process=5be3cc137c874226&seqtype=1) [gnl|unipsp|O15259](http://www.uniprot.org/uniprot/O15259) [Eukaryota] Nephrocystin-1 [Metazoa][Homo sapiens] (length=732 residues).

Site : 243- 248, Identity

etadgaevkq_RTDPHW_savqkaisea

--------------------------------------------------------------------------------

[NPSA](https://npsa-prabi.ibcp.fr/cgi-bin/seq_methonseq.pl?seqid=O15439&db=unipsp&process=5be3cc137c874227&seqtype=1) [gnl|unipsp|O15439](http://www.uniprot.org/uniprot/O15439) [Eukaryota] Multidrug resistance-associated protein 4 [Metazoa][Homo sapiens] (length=1325 residues).

Site : 1030- 1035, Identity

ekeapweyqk_RPPPAW_phegviifdn

--------------------------------------------------------------------------------

[NPSA](https://npsa-prabi.ibcp.fr/cgi-bin/seq_methonseq.pl?seqid=O15552&db=unipsp&process=5be3cc137c874228&seqtype=1) [gnl|unipsp|O15552](http://www.uniprot.org/uniprot/O15552) [Eukaryota] Free fatty acid receptor 2 [Metazoa][Homo sapiens] (length=330 residues).

Site : 249- 254, Identity

nvshlvgyhq_RKSPWW_rsiavvfssl

--------------------------------------------------------------------------------

[NPSA](https://npsa-prabi.ibcp.fr/cgi-bin/seq_methonseq.pl?seqid=O43491&db=unipsp&process=5be3cc137c874229&seqtype=1) [gnl|unipsp|O43491](http://www.uniprot.org/uniprot/O43491) [Eukaryota] Band 4.1-like protein 2 [Metazoa][Homo sapiens] (length=1005 residues).

Site : 76- 81, Identity

ketsesrgis_RFIPPW_lkkqksytlv

--------------------------------------------------------------------------------

[NPSA](https://npsa-prabi.ibcp.fr/cgi-bin/seq_methonseq.pl?seqid=O60303&db=unipsp&process=5be3cc137c874230&seqtype=1) [gnl|unipsp|O60303](http://www.uniprot.org/uniprot/O60303) [Eukaryota] Uncharacterized protein KIAA0556 [Metazoa][Homo sapiens] (length=1618 residues).

Site : 809- 814, Identity

geteardkgl_RHEPGW_gtsrsvntke

--------------------------------------------------------------------------------

[NPSA](https://npsa-prabi.ibcp.fr/cgi-bin/seq_methonseq.pl?seqid=O60391&db=unipsp&process=5be3cc137c874231&seqtype=1) [gnl|unipsp|O60391](http://www.uniprot.org/uniprot/O60391) [Eukaryota] Glutamate receptor ionotropic, NMDA 3B [Metazoa][Homo sapiens] (length=1043 residues).

Site : 350- 355, Identity

rflantsfqg_RTGPVW_vtgssqvhms

Site : 378- 383, Identity

fkvwslrrdp_RGAPAW_atvgswrdgq

Site : 957- 962, Identity

eadaeaeaap_REGPVW_lcsygrppaa

--------------------------------------------------------------------------------

[NPSA](https://npsa-prabi.ibcp.fr/cgi-bin/seq_methonseq.pl?seqid=O60733&db=unipsp&process=5be3cc137c874232&seqtype=1) [gnl|unipsp|O60733](http://www.uniprot.org/uniprot/O60733) [Eukaryota] 85/88 kDa calcium-independent phospholipase A2 [Metazoa][Homo sapiens] (length=806 residues).

Site : 115- 120, Identity

evlqhltdli_RNHPSW_svahlavelg

--------------------------------------------------------------------------------

[NPSA](https://npsa-prabi.ibcp.fr/cgi-bin/seq_methonseq.pl?seqid=O75051&db=unipsp&process=5be3cc137c874233&seqtype=1) [gnl|unipsp|O75051](http://www.uniprot.org/uniprot/O75051) [Eukaryota] Plexin-A2 [Metazoa][Homo sapiens] (length=1894 residues).

Site : 1045- 1050, Identity

feyiddprvq_RIEPEW_siasghtplt

--------------------------------------------------------------------------------

[NPSA](https://npsa-prabi.ibcp.fr/cgi-bin/seq_methonseq.pl?seqid=O75127&db=unipsp&process=5be3cc137c874234&seqtype=1) [gnl|unipsp|O75127](http://www.uniprot.org/uniprot/O75127) [Eukaryota] Pentatricopeptide repeat-containing protein 1, mitochondrial [Metazoa][Homo sapiens] (length=700 residues).

Site : 133- 138, Identity

epepklwrgr_RNTPYW_yflqckhlik

--------------------------------------------------------------------------------

[NPSA](https://npsa-prabi.ibcp.fr/cgi-bin/seq_methonseq.pl?seqid=O75335&db=unipsp&process=5be3cc137c874235&seqtype=1) [gnl|unipsp|O75335](http://www.uniprot.org/uniprot/O75335) [Eukaryota] Liprin-alpha-4 [Metazoa][Homo sapiens] (length=1185 residues).

Site : 1116- 1121, Identity

klddgddkvf_RRAPSW_rkrfrprehh

--------------------------------------------------------------------------------

[NPSA](https://npsa-prabi.ibcp.fr/cgi-bin/seq_methonseq.pl?seqid=O75376&db=unipsp&process=5be3cc137c874236&seqtype=1) [gnl|unipsp|O75376](http://www.uniprot.org/uniprot/O75376) [Eukaryota] Nuclear receptor corepressor 1 [Metazoa][Homo sapiens] (length=2440 residues).

Site : 2364- 2369, Identity

ssvhsegdyh_RQTPGW_awedrpsstg

--------------------------------------------------------------------------------

[NPSA](https://npsa-prabi.ibcp.fr/cgi-bin/seq_methonseq.pl?seqid=O75880&db=unipsp&process=5be3cc137c874237&seqtype=1) [gnl|unipsp|O75880](http://www.uniprot.org/uniprot/O75880) [Eukaryota] Protein SCO1 homolog, mitochondrial [Metazoa][Homo sapiens] (length=301 residues).

Site : 71- 76, Identity

clgtrplsta_RPPPPW_sqkgpgdstr

--------------------------------------------------------------------------------

[NPSA](https://npsa-prabi.ibcp.fr/cgi-bin/seq_methonseq.pl?seqid=O75912&db=unipsp&process=5be3cc137c874238&seqtype=1) [gnl|unipsp|O75912](http://www.uniprot.org/uniprot/O75912) [Eukaryota] Diacylglycerol kinase iota [Metazoa][Homo sapiens] (length=1065 residues).

Site : 823- 828, Identity

tsfpralsaq_RLSPRW_cflddrsqeh

--------------------------------------------------------------------------------

[NPSA](https://npsa-prabi.ibcp.fr/cgi-bin/seq_methonseq.pl?seqid=O94844&db=unipsp&process=5be3cc137c874239&seqtype=1) [gnl|unipsp|O94844](http://www.uniprot.org/uniprot/O94844) [Eukaryota] Rho-related BTB domain-containing protein 1 [Metazoa][Homo sapiens] (length=696 residues).

Site : 650- 655, Identity

adnqeyferh_RWPPVW_ylkeedhyqr

--------------------------------------------------------------------------------

[NPSA](https://npsa-prabi.ibcp.fr/cgi-bin/seq_methonseq.pl?seqid=O94925&db=unipsp&process=5be3cc137c874240&seqtype=1) [gnl|unipsp|O94925](http://www.uniprot.org/uniprot/O94925) [Eukaryota] Glutaminase kidney isoform, mitochondrial [Metazoa][Homo sapiens] (length=669 residues).

Site : 53- 58, Identity

rpaagpaaaa_RLHPWW_ggggwpaepl

--------------------------------------------------------------------------------

[NPSA](https://npsa-prabi.ibcp.fr/cgi-bin/seq_methonseq.pl?seqid=O94964&db=unipsp&process=5be3cc137c874241&seqtype=1) [gnl|unipsp|O94964](http://www.uniprot.org/uniprot/O94964) [Eukaryota] Protein SOGA1 [Metazoa][Homo sapiens] (length=1423 residues).

Site : 539- 544, Identity

lgsdfqppdf_RDLPEW_eprireafrt

Site : 803- 808, Identity

elktgkgage_RAGPDW_kaalqreree

--------------------------------------------------------------------------------

[NPSA](https://npsa-prabi.ibcp.fr/cgi-bin/seq_methonseq.pl?seqid=O95158&db=unipsp&process=5be3cc137c874242&seqtype=1) [gnl|unipsp|O95158](http://www.uniprot.org/uniprot/O95158) [Eukaryota] Neurexophilin-4 [Metazoa][Homo sapiens] (length=308 residues).

Site : 2- 7, Identity

‹m_RLLPEW_flllfgpwll

--------------------------------------------------------------------------------

[NPSA](https://npsa-prabi.ibcp.fr/cgi-bin/seq_methonseq.pl?seqid=O95398&db=unipsp&process=5be3cc137c874243&seqtype=1) [gnl|unipsp|O95398](http://www.uniprot.org/uniprot/O95398) [Eukaryota] Rap guanine nucleotide exchange factor 3 [Metazoa][Homo sapiens] (length=923 residues).

Site : 534- 539, Identity

cgnaspqmka_RNLPVW_lpnqdeplpg

--------------------------------------------------------------------------------

[NPSA](https://npsa-prabi.ibcp.fr/cgi-bin/seq_methonseq.pl?seqid=O95613&db=unipsp&process=5be3cc137c874244&seqtype=1) [gnl|unipsp|O95613](http://www.uniprot.org/uniprot/O95613) [Eukaryota] Pericentrin [Metazoa][Homo sapiens] (length=3336 residues).

Site : 2865- 2870, Identity

relrcslere_REKPAW_lqaeleqshp

--------------------------------------------------------------------------------

[NPSA](https://npsa-prabi.ibcp.fr/cgi-bin/seq_methonseq.pl?seqid=P00797&db=unipsp&process=5be3cc137c874245&seqtype=1) [gnl|unipsp|P00797](http://www.uniprot.org/uniprot/P00797) [Eukaryota] Renin [Metazoa][Homo sapiens] (length=406 residues).

Site : 5- 10, Identity

‹mdgw_RRMPRW_glllllwgsc

Site : 55- 60, Identity

slkergvdma_RLGPEW_sqpmkrltlg

--------------------------------------------------------------------------------

[NPSA](https://npsa-prabi.ibcp.fr/cgi-bin/seq_methonseq.pl?seqid=P00813&db=unipsp&process=5be3cc137c874246&seqtype=1) [gnl|unipsp|P00813](http://www.uniprot.org/uniprot/P00813) [Eukaryota] Adenosine deaminase [Metazoa][Homo sapiens] (length=363 residues).

Site : 156- 161, Identity

vkarsilccm_RHQPNW_spkvvelckk

--------------------------------------------------------------------------------

[NPSA](https://npsa-prabi.ibcp.fr/cgi-bin/seq_methonseq.pl?seqid=P01266&db=unipsp&process=5be3cc137c874247&seqtype=1) [gnl|unipsp|P01266](http://www.uniprot.org/uniprot/P01266) [Eukaryota] Thyroglobulin [Metazoa][Homo sapiens] (length=2768 residues).

Site : 1549- 1554, Identity

gkafcvdgeg_RRLPWW_eteapledsq

--------------------------------------------------------------------------------

[NPSA](https://npsa-prabi.ibcp.fr/cgi-bin/seq_methonseq.pl?seqid=P01589&db=unipsp&process=5be3cc137c874248&seqtype=1) [gnl|unipsp|P01589](http://www.uniprot.org/uniprot/P01589) [Eukaryota] Interleukin-2 receptor subunit alpha [Metazoa][Homo sapiens] (length=272 residues).

Site : 126- 131, Identity

vdqaslpghc_REPPPW_eneateriyh

--------------------------------------------------------------------------------

[NPSA](https://npsa-prabi.ibcp.fr/cgi-bin/seq_methonseq.pl?seqid=P04066&db=unipsp&process=5be3cc137c874249&seqtype=1) [gnl|unipsp|P04066](http://www.uniprot.org/uniprot/P04066) [Eukaryota] Tissue alpha-L-fucosidase [Metazoa][Homo sapiens] (length=466 residues).

Site : 36- 41, Identity

esvrraqppr_RYTPDW_psldsrplpa

Site : 47- 52, Identity

ytpdwpslds_RPLPAW_fdeakfgvfi

--------------------------------------------------------------------------------

[NPSA](https://npsa-prabi.ibcp.fr/cgi-bin/seq_methonseq.pl?seqid=P05106&db=unipsp&process=5be3cc137c874250&seqtype=1) [gnl|unipsp|P05106](http://www.uniprot.org/uniprot/P05106) [Eukaryota] Integrin beta-3 [Metazoa][Homo sapiens] (length=788 residues).

Site : 6- 11, Identity

‹mrarp_RPRPLW_atvlalgala

Site : 487- 492, Identity

gngtfecgvc_RCGPGW_lgsqcecsee

--------------------------------------------------------------------------------

[NPSA](https://npsa-prabi.ibcp.fr/cgi-bin/seq_methonseq.pl?seqid=P06213&db=unipsp&process=5be3cc137c874251&seqtype=1) [gnl|unipsp|P06213](http://www.uniprot.org/uniprot/P06213) [Eukaryota] Insulin receptor [Metazoa][Homo sapiens] (length=1382 residues).

Site : 515- 520, Identity

irtsfdkill_RWEPYW_ppdfrdllgf

--------------------------------------------------------------------------------

[NPSA](https://npsa-prabi.ibcp.fr/cgi-bin/seq_methonseq.pl?seqid=P07093&db=unipsp&process=5be3cc137c874252&seqtype=1) [gnl|unipsp|P07093](http://www.uniprot.org/uniprot/P07093) [Eukaryota] Glia-derived nexin [Metazoa][Homo sapiens] (length=398 residues).

Site : 365- 370, Identity

saattailia_RSSPPW_fivdrpflff

--------------------------------------------------------------------------------

[NPSA](https://npsa-prabi.ibcp.fr/cgi-bin/seq_methonseq.pl?seqid=P07202&db=unipsp&process=5be3cc137c874253&seqtype=1) [gnl|unipsp|P07202](http://www.uniprot.org/uniprot/P07202) [Eukaryota] Thyroid peroxidase [Metazoa][Homo sapiens] (length=933 residues).

Site : 161- 166, Identity

yrpitgacnn_RDHPRW_gasntalarw

--------------------------------------------------------------------------------

[NPSA](https://npsa-prabi.ibcp.fr/cgi-bin/seq_methonseq.pl?seqid=P07738&db=unipsp&process=5be3cc137c874254&seqtype=1) [gnl|unipsp|P07738](http://www.uniprot.org/uniprot/P07738) [Eukaryota] Bisphosphoglycerate mutase [Metazoa][Homo sapiens] (length=259 residues).

Site : 164- 169, Identity

rseslkdvle_RLLPYW_neriapevlr

--------------------------------------------------------------------------------

[NPSA](https://npsa-prabi.ibcp.fr/cgi-bin/seq_methonseq.pl?seqid=P08514&db=unipsp&process=5be3cc137c874255&seqtype=1) [gnl|unipsp|P08514](http://www.uniprot.org/uniprot/P08514) [Eukaryota] Integrin alpha-IIb [Metazoa][Homo sapiens] (length=1039 residues).

Site : 993- 998, Identity

wtqllralee_RAIPIW_wvlvgvlggl

--------------------------------------------------------------------------------

[NPSA](https://npsa-prabi.ibcp.fr/cgi-bin/seq_methonseq.pl?seqid=P10635&db=unipsp&process=5be3cc137c874256&seqtype=1) [gnl|unipsp|P10635](http://www.uniprot.org/uniprot/P10635) [Eukaryota] Cytochrome P450 2D6 [Metazoa][Homo sapiens] (length=497 residues).

Site : 123- 128, Identity

gprsqgvfla_RYGPAW_reqrrfsvst

--------------------------------------------------------------------------------

[NPSA](https://npsa-prabi.ibcp.fr/cgi-bin/seq_methonseq.pl?seqid=P11388&db=unipsp&process=5be3cc137c874257&seqtype=1) [gnl|unipsp|P11388](http://www.uniprot.org/uniprot/P11388) [Eukaryota] DNA topoisomerase 2-alpha [Metazoa][Homo sapiens] (length=1531 residues).

Site : 835- 840, Identity

tlkflyddnq_RVEPEW_yipiipmvli

--------------------------------------------------------------------------------

[NPSA](https://npsa-prabi.ibcp.fr/cgi-bin/seq_methonseq.pl?seqid=P11678&db=unipsp&process=5be3cc137c874258&seqtype=1) [gnl|unipsp|P11678](http://www.uniprot.org/uniprot/P11678) [Eukaryota] Eosinophil peroxidase [Metazoa][Homo sapiens] (length=715 residues).

Site : 404- 409, Identity

ehnrlatelr_RLNPRW_ngdklynear

--------------------------------------------------------------------------------

[NPSA](https://npsa-prabi.ibcp.fr/cgi-bin/seq_methonseq.pl?seqid=P11717&db=unipsp&process=5be3cc137c874259&seqtype=1) [gnl|unipsp|P11717](http://www.uniprot.org/uniprot/P11717) [Eukaryota] Cation-independent mannose-6-phosphate receptor [Metazoa][Homo sapiens] (length=2491 residues).

Site : 1441- 1446, Identity

gskpvnlgrv_RDGPQW_rdgiivlkyv

--------------------------------------------------------------------------------

[NPSA](https://npsa-prabi.ibcp.fr/cgi-bin/seq_methonseq.pl?seqid=P11940&db=unipsp&process=5be3cc137c874260&seqtype=1) [gnl|unipsp|P11940](http://www.uniprot.org/uniprot/P11940) [Eukaryota] Polyadenylate-binding protein 1 [Metazoa][Homo sapiens] (length=636 residues).

Site : 432- 437, Identity

yyppsqiaql_RPSPRW_taqgarphpf

--------------------------------------------------------------------------------

[NPSA](https://npsa-prabi.ibcp.fr/cgi-bin/seq_methonseq.pl?seqid=P15259&db=unipsp&process=5be3cc137c874261&seqtype=1) [gnl|unipsp|P15259](http://www.uniprot.org/uniprot/P15259) [Eukaryota] Phosphoglycerate mutase 2 [Metazoa][Homo sapiens] (length=253 residues).

Site : 162- 167, Identity

tceslkdtia_RALPFW_neeivpqika

--------------------------------------------------------------------------------

[NPSA](https://npsa-prabi.ibcp.fr/cgi-bin/seq_methonseq.pl?seqid=P17861&db=unipsp&process=5be3cc137c874262&seqtype=1) [gnl|unipsp|P17861](http://www.uniprot.org/uniprot/P17861) [Eukaryota] X-box-binding protein 1 [Metazoa][Homo sapiens] (length=261 residues).

Site : 251- 256, Identity

yqppflcqwg_RHQPSW_kplmn›

--------------------------------------------------------------------------------

[NPSA](https://npsa-prabi.ibcp.fr/cgi-bin/seq_methonseq.pl?seqid=P18085&db=unipsp&process=5be3cc137c874263&seqtype=1) [gnl|unipsp|P18085](http://www.uniprot.org/uniprot/P18085) [Eukaryota] ADP-ribosylation factor 4 [Metazoa][Homo sapiens] (length=180 residues).

Site : 73- 78, Identity

ftvwdvggqd_RIRPLW_khyfqntqgl

--------------------------------------------------------------------------------

[NPSA](https://npsa-prabi.ibcp.fr/cgi-bin/seq_methonseq.pl?seqid=P18577&db=unipsp&process=5be3cc137c874264&seqtype=1) [gnl|unipsp|P18577](http://www.uniprot.org/uniprot/P18577) [Eukaryota] Blood group Rh(CE) polypeptide [Metazoa][Homo sapiens] (length=417 residues).

Site : 11- 16, Identity

msskyprsvr_RCLPLW_altleaalil

--------------------------------------------------------------------------------

[NPSA](https://npsa-prabi.ibcp.fr/cgi-bin/seq_methonseq.pl?seqid=P18669&db=unipsp&process=5be3cc137c874265&seqtype=1) [gnl|unipsp|P18669](http://www.uniprot.org/uniprot/P18669) [Eukaryota] Phosphoglycerate mutase 1 [Metazoa][Homo sapiens] (length=254 residues).

Site : 162- 167, Identity

sceslkdtia_RALPFW_neeivpqike

--------------------------------------------------------------------------------

[NPSA](https://npsa-prabi.ibcp.fr/cgi-bin/seq_methonseq.pl?seqid=P21980&db=unipsp&process=5be3cc137c874266&seqtype=1) [gnl|unipsp|P21980](http://www.uniprot.org/uniprot/P21980) [Eukaryota] Protein-glutamine gamma-glutamyltransferase 2 [Metazoa][Homo sapiens] (length=687 residues).

Site : 35- 40, Identity

dlcreklvvr_RGQPFW_ltlhfegrny

--------------------------------------------------------------------------------

[NPSA](https://npsa-prabi.ibcp.fr/cgi-bin/seq_methonseq.pl?seqid=P22079&db=unipsp&process=5be3cc137c874267&seqtype=1) [gnl|unipsp|P22079](http://www.uniprot.org/uniprot/P22079) [Eukaryota] Lactoperoxidase [Metazoa][Homo sapiens] (length=712 residues).

Site : 399- 404, Identity

ehnrlarelk_RLNPQW_dgeklyqear

--------------------------------------------------------------------------------

[NPSA](https://npsa-prabi.ibcp.fr/cgi-bin/seq_methonseq.pl?seqid=P22083&db=unipsp&process=5be3cc137c874268&seqtype=1) [gnl|unipsp|P22083](http://www.uniprot.org/uniprot/P22083) [Eukaryota] Alpha-(1,3)-fucosyltransferase 4 [Metazoa][Homo sapiens] (length=530 residues).

Site : 45- 50, Identity

lgpgrsgrkg_RAVPGW_aswpahlala

--------------------------------------------------------------------------------

[NPSA](https://npsa-prabi.ibcp.fr/cgi-bin/seq_methonseq.pl?seqid=P24592&db=unipsp&process=5be3cc137c874269&seqtype=1) [gnl|unipsp|P24592](http://www.uniprot.org/uniprot/P24592) [Eukaryota] Insulin-like growth factor-binding protein 6 [Metazoa][Homo sapiens] (length=240 residues).

Site : 208- 213, Identity

krqcrssqgq_RRGPCW_cvdrmgkslp

--------------------------------------------------------------------------------

[NPSA](https://npsa-prabi.ibcp.fr/cgi-bin/seq_methonseq.pl?seqid=P25100&db=unipsp&process=5be3cc137c874270&seqtype=1) [gnl|unipsp|P25100](http://www.uniprot.org/uniprot/P25100) [Eukaryota] Alpha-1D adrenergic receptor [Metazoa][Homo sapiens] (length=572 residues).

Site : 426- 431, Identity

llrcqcrrrr_RRRPLW_rvyghhwras

--------------------------------------------------------------------------------

[NPSA](https://npsa-prabi.ibcp.fr/cgi-bin/seq_methonseq.pl?seqid=P26842&db=unipsp&process=5be3cc137c874271&seqtype=1) [gnl|unipsp|P26842](http://www.uniprot.org/uniprot/P26842) [Eukaryota] CD27 antigen [Metazoa][Homo sapiens] (length=260 residues).

Site : 3- 8, Identity

‹ma_RPHPWW_lcvlgtlvgl

--------------------------------------------------------------------------------

[NPSA](https://npsa-prabi.ibcp.fr/cgi-bin/seq_methonseq.pl?seqid=P28799&db=unipsp&process=5be3cc137c874272&seqtype=1) [gnl|unipsp|P28799](http://www.uniprot.org/uniprot/P28799) [Eukaryota] Granulins [Metazoa][Homo sapiens] (length=593 residues).

Site : 575- 580, Identity

caargtkclr_REAPRW_daplrdpalr

--------------------------------------------------------------------------------

[NPSA](https://npsa-prabi.ibcp.fr/cgi-bin/seq_methonseq.pl?seqid=P35610&db=unipsp&process=5be3cc137c874273&seqtype=1) [gnl|unipsp|P35610](http://www.uniprot.org/uniprot/P35610) [Eukaryota] Sterol O-acyltransferase 1 [Metazoa][Homo sapiens] (length=550 residues).

Site : 494- 499, Identity

mafnfivnds_RKKPIW_nvlmwtslfl

--------------------------------------------------------------------------------

[NPSA](https://npsa-prabi.ibcp.fr/cgi-bin/seq_methonseq.pl?seqid=P36956&db=unipsp&process=5be3cc137c874274&seqtype=1) [gnl|unipsp|P36956](http://www.uniprot.org/uniprot/P36956) [Eukaryota] Sterol regulatory element-binding protein 1 [Metazoa][Homo sapiens] (length=1147 residues).

Site : 535- 540, Identity

pgrnvlgtes_RDGPGW_aqwllppvvw

--------------------------------------------------------------------------------

[NPSA](https://npsa-prabi.ibcp.fr/cgi-bin/seq_methonseq.pl?seqid=P42167&db=unipsp&process=5be3cc137c874275&seqtype=1) [gnl|unipsp|P42167](http://www.uniprot.org/uniprot/P42167) [Eukaryota] Lamina-associated polypeptide 2, isoforms beta/gamma [Metazoa][Homo sapiens] (length=454 residues).

Site : 409- 414, Identity

dvksektkkg_RSIPVW_ikillfvvva

--------------------------------------------------------------------------------

[NPSA](https://npsa-prabi.ibcp.fr/cgi-bin/seq_methonseq.pl?seqid=P42226&db=unipsp&process=5be3cc137c874276&seqtype=1) [gnl|unipsp|P42226](http://www.uniprot.org/uniprot/P42226) [Eukaryota] Signal transducer and activator of transcription 6 [Metazoa][Homo sapiens] (length=847 residues).

Site : 842- 847, Identity

sgismshmdl_RANPSW_›

--------------------------------------------------------------------------------

[NPSA](https://npsa-prabi.ibcp.fr/cgi-bin/seq_methonseq.pl?seqid=P42785&db=unipsp&process=5be3cc137c874277&seqtype=1) [gnl|unipsp|P42785](http://www.uniprot.org/uniprot/P42785) [Eukaryota] Lysosomal Pro-X carboxypeptidase [Metazoa][Homo sapiens] (length=496 residues).

Site : 401- 406, Identity

sddcfqqwgv_RPRPSW_ittmyggkni

--------------------------------------------------------------------------------

[NPSA](https://npsa-prabi.ibcp.fr/cgi-bin/seq_methonseq.pl?seqid=P43363&db=unipsp&process=5be3cc137c874278&seqtype=1) [gnl|unipsp|P43363](http://www.uniprot.org/uniprot/P43363) [Eukaryota] Melanoma-associated antigen 10 [Metazoa][Homo sapiens] (length=369 residues).

Site : 324- 329, Identity

flakvngsdp_RSFPLW_yeealkdeee

--------------------------------------------------------------------------------

[NPSA](https://npsa-prabi.ibcp.fr/cgi-bin/seq_methonseq.pl?seqid=P46531&db=unipsp&process=5be3cc137c874279&seqtype=1) [gnl|unipsp|P46531](http://www.uniprot.org/uniprot/P46531) [Eukaryota] Neurogenic locus notch homolog protein 1 [Metazoa][Homo sapiens] (length=2555 residues).

Site : 128- 133, Identity

dlltlteykc_RCPPGW_sgkscqqadp

Site : 282- 287, Identity

cvdgvntync_RCPPEW_tgqyctedvd

--------------------------------------------------------------------------------

[NPSA](https://npsa-prabi.ibcp.fr/cgi-bin/seq_methonseq.pl?seqid=P48553&db=unipsp&process=5be3cc137c874280&seqtype=1) [gnl|unipsp|P48553](http://www.uniprot.org/uniprot/P48553) [Eukaryota] Trafficking protein particle complex subunit 10 [Metazoa][Homo sapiens] (length=1259 residues).

Site : 212- 217, Identity

eddmrtlrek_RTEPGW_sfceyfmvqe

--------------------------------------------------------------------------------

[NPSA](https://npsa-prabi.ibcp.fr/cgi-bin/seq_methonseq.pl?seqid=P48735&db=unipsp&process=5be3cc137c874281&seqtype=1) [gnl|unipsp|P48735](http://www.uniprot.org/uniprot/P48735) [Eukaryota] Isocitrate dehydrogenase [NADP], mitochondrial [Metazoa][Homo sapiens] (length=452 residues).

Site : 159- 164, Identity

repiicknip_RLVPGW_tkpitigrha

--------------------------------------------------------------------------------

[NPSA](https://npsa-prabi.ibcp.fr/cgi-bin/seq_methonseq.pl?seqid=P48751&db=unipsp&process=5be3cc137c874282&seqtype=1) [gnl|unipsp|P48751](http://www.uniprot.org/uniprot/P48751) [Eukaryota] Anion exchange protein 3 [Metazoa][Homo sapiens] (length=1232 residues).

Site : 979- 984, Identity

swfipplgsa_RPFPPW_mmvaaavpal

--------------------------------------------------------------------------------

[NPSA](https://npsa-prabi.ibcp.fr/cgi-bin/seq_methonseq.pl?seqid=P48960&db=unipsp&process=5be3cc137c874283&seqtype=1) [gnl|unipsp|P48960](http://www.uniprot.org/uniprot/P48960) [Eukaryota] CD97 antigen [Metazoa][Homo sapiens] (length=835 residues).

Site : 187- 192, Identity

clnnvgsyqc_RCRPGW_qpipgspngp

Site : 236- 241, Identity

cfntvgsysc_RCRPGW_kprhgipnnq

--------------------------------------------------------------------------------

[NPSA](https://npsa-prabi.ibcp.fr/cgi-bin/seq_methonseq.pl?seqid=P49589&db=unipsp&process=5be3cc137c874284&seqtype=1) [gnl|unipsp|P49589](http://www.uniprot.org/uniprot/P49589) [Eukaryota] Cysteine--tRNA ligase, cytoplasmic [Metazoa][Homo sapiens] (length=748 residues).

Site : 13- 18, Identity

dssgqqgkgr_RVQPQW_sppagtqpcr

--------------------------------------------------------------------------------

[NPSA](https://npsa-prabi.ibcp.fr/cgi-bin/seq_methonseq.pl?seqid=P49750&db=unipsp&process=5be3cc137c874285&seqtype=1) [gnl|unipsp|P49750](http://www.uniprot.org/uniprot/P49750) [Eukaryota] YLP motif-containing protein 1 [Metazoa][Homo sapiens] (length=1951 residues).

Site : 1065- 1070, Identity

appsrshdgd_RRGPWW_ddwerdqdmd

--------------------------------------------------------------------------------

[NPSA](https://npsa-prabi.ibcp.fr/cgi-bin/seq_methonseq.pl?seqid=P49765&db=unipsp&process=5be3cc137c874286&seqtype=1) [gnl|unipsp|P49765](http://www.uniprot.org/uniprot/P49765) [Eukaryota] Vascular endothelial growth factor B [Metazoa][Homo sapiens] (length=207 residues).

Site : 148- 153, Identity

aatphhrpqp_RSVPGW_dsapgapspa

--------------------------------------------------------------------------------

[NPSA](https://npsa-prabi.ibcp.fr/cgi-bin/seq_methonseq.pl?seqid=P50402&db=unipsp&process=5be3cc137c874287&seqtype=1) [gnl|unipsp|P50402](http://www.uniprot.org/uniprot/P50402) [Eukaryota] Emerin [Metazoa][Homo sapiens] (length=254 residues).

Site : 221- 226, Identity

rapgaglgqd_RQVPLW_gqlllflvfv

--------------------------------------------------------------------------------

[NPSA](https://npsa-prabi.ibcp.fr/cgi-bin/seq_methonseq.pl?seqid=P50876&db=unipsp&process=5be3cc137c874288&seqtype=1) [gnl|unipsp|P50876](http://www.uniprot.org/uniprot/P50876) [Eukaryota] E3 ubiquitin-protein ligase RNF144A [Metazoa][Homo sapiens] (length=292 residues).

Site : 5- 10, Identity

‹mttt_RYRPTW_dlaldplvsc

--------------------------------------------------------------------------------

[NPSA](https://npsa-prabi.ibcp.fr/cgi-bin/seq_methonseq.pl?seqid=P51168&db=unipsp&process=5be3cc137c874289&seqtype=1) [gnl|unipsp|P51168](http://www.uniprot.org/uniprot/P51168) [Eukaryota] Amiloride-sensitive sodium channel subunit beta [Metazoa][Homo sapiens] (length=640 residues).

Site : 418- 423, Identity

lprgekycnn_RDFPDW_ahcysdlqms

--------------------------------------------------------------------------------

[NPSA](https://npsa-prabi.ibcp.fr/cgi-bin/seq_methonseq.pl?seqid=P51805&db=unipsp&process=5be3cc137c874290&seqtype=1) [gnl|unipsp|P51805](http://www.uniprot.org/uniprot/P51805) [Eukaryota] Plexin-A3 [Metazoa][Homo sapiens] (length=1871 residues).

Site : 1027- 1032, Identity

ytytqdptvt_RLEPTW_siingstait

--------------------------------------------------------------------------------

[NPSA](https://npsa-prabi.ibcp.fr/cgi-bin/seq_methonseq.pl?seqid=P54619&db=unipsp&process=5be3cc137c874291&seqtype=1) [gnl|unipsp|P54619](http://www.uniprot.org/uniprot/P54619) [Eukaryota] 5'-AMP-activated protein kinase subunit gamma-1 [Metazoa][Homo sapiens] (length=331 residues).

Site : 70- 75, Identity

affalvtngv_RAAPLW_dskkqsfvgm

--------------------------------------------------------------------------------

[NPSA](https://npsa-prabi.ibcp.fr/cgi-bin/seq_methonseq.pl?seqid=P54756&db=unipsp&process=5be3cc137c874292&seqtype=1) [gnl|unipsp|P54756](http://www.uniprot.org/uniprot/P54756) [Eukaryota] Ephrin type-A receptor 5 [Metazoa][Homo sapiens] (length=1037 residues).

Site : 36- 41, Identity

aslagcysap_RRAPLW_tclllcaalr

--------------------------------------------------------------------------------

[NPSA](https://npsa-prabi.ibcp.fr/cgi-bin/seq_methonseq.pl?seqid=P55786&db=unipsp&process=5be3cc137c874293&seqtype=1) [gnl|unipsp|P55786](http://www.uniprot.org/uniprot/P55786) [Eukaryota] Puromycin-sensitive aminopeptidase [Metazoa][Homo sapiens] (length=919 residues).

Site : 186- 191, Identity

vtqfeatdar_RAFPCW_depaikatfd

--------------------------------------------------------------------------------

[NPSA](https://npsa-prabi.ibcp.fr/cgi-bin/seq_methonseq.pl?seqid=P56180&db=unipsp&process=5be3cc137c874294&seqtype=1) [gnl|unipsp|P56180](http://www.uniprot.org/uniprot/P56180) [Eukaryota] Putative tyrosine-protein phosphatase TPTE [Metazoa][Homo sapiens] (length=551 residues).

Site : 185- 190, Identity

vyiffdikll_RNIPRW_thllrllrli

--------------------------------------------------------------------------------

[NPSA](https://npsa-prabi.ibcp.fr/cgi-bin/seq_methonseq.pl?seqid=P58107&db=unipsp&process=5be3cc137c874295&seqtype=1) [gnl|unipsp|P58107](http://www.uniprot.org/uniprot/P58107) [Eukaryota] Epiplakin [Metazoa][Homo sapiens] (length=5090 residues).

Site : 2532- 2537, Identity

mevkvgrlrg_RAVPVW_dvlasgyvsr

Site : 3066- 3071, Identity

mevkvgrlrg_RAVPVW_dvlasgyvsg

Site : 3600- 3605, Identity

mevkvgrlrg_RAVPVW_dvlasgyvsg

Site : 4134- 4139, Identity

mevkvgrlrg_RAVPVW_dvlasgyvsr

Site : 4668- 4673, Identity

mevkvgrlrg_RAVPVW_dvlasgyvsg

--------------------------------------------------------------------------------

[NPSA](https://npsa-prabi.ibcp.fr/cgi-bin/seq_methonseq.pl?seqid=P59020&db=unipsp&process=5be3cc137c874296&seqtype=1) [gnl|unipsp|P59020](http://www.uniprot.org/uniprot/P59020) [Eukaryota] Down syndrome critical region protein 9 [Metazoa][Homo sapiens] (length=149 residues).

Site : 81- 86, Identity

gavlqrmlgr_RAPPSW_srdhaysrrg

--------------------------------------------------------------------------------

[NPSA](https://npsa-prabi.ibcp.fr/cgi-bin/seq_methonseq.pl?seqid=P62277&db=unipsp&process=5be3cc137c874297&seqtype=1) [gnl|unipsp|P62277](http://www.uniprot.org/uniprot/P62277) [Eukaryota] 40S ribosomal protein S13 [Metazoa][Homo sapiens] (length=151 residues).

Site : 20- 25, Identity

glsqsalpyr_RSVPTW_lkltsddvke

--------------------------------------------------------------------------------

[NPSA](https://npsa-prabi.ibcp.fr/cgi-bin/seq_methonseq.pl?seqid=P62891&db=unipsp&process=5be3cc137c874298&seqtype=1) [gnl|unipsp|P62891](http://www.uniprot.org/uniprot/P62891) [Eukaryota] 60S ribosomal protein L39 [Metazoa][Homo sapiens] (length=51 residues).

Site : 21- 26, Identity

rflakkqkqn_RPIPQW_irmktgnkir

--------------------------------------------------------------------------------

[NPSA](https://npsa-prabi.ibcp.fr/cgi-bin/seq_methonseq.pl?seqid=P78332&db=unipsp&process=5be3cc137c874299&seqtype=1) [gnl|unipsp|P78332](http://www.uniprot.org/uniprot/P78332) [Eukaryota] RNA-binding protein 6 [Metazoa][Homo sapiens] (length=1123 residues).

Site : 21- 26, Identity

tgpfrgsqee_RFAPGW_nrdypppplk

--------------------------------------------------------------------------------

[NPSA](https://npsa-prabi.ibcp.fr/cgi-bin/seq_methonseq.pl?seqid=P78363&db=unipsp&process=5be3cc137c874300&seqtype=1) [gnl|unipsp|P78363](http://www.uniprot.org/uniprot/P78363) [Eukaryota] Retinal-specific ATP-binding cassette transporter [Metazoa][Homo sapiens] (length=2273 residues).

Site : 920- 925, Identity

pegihdsffe_REHPGW_vpgvcvknlv

--------------------------------------------------------------------------------

[NPSA](https://npsa-prabi.ibcp.fr/cgi-bin/seq_methonseq.pl?seqid=P78527&db=unipsp&process=5be3cc137c874301&seqtype=1) [gnl|unipsp|P78527](http://www.uniprot.org/uniprot/P78527) [Eukaryota] DNA-dependent protein kinase catalytic subunit [Metazoa][Homo sapiens] (length=4128 residues).

Site : 2120- 2125, Identity

ppqgeedsvp_RDLPSW_mkflhgklgn

--------------------------------------------------------------------------------

[NPSA](https://npsa-prabi.ibcp.fr/cgi-bin/seq_methonseq.pl?seqid=P78560&db=unipsp&process=5be3cc137c874302&seqtype=1) [gnl|unipsp|P78560](http://www.uniprot.org/uniprot/P78560) [Eukaryota] Death domain-containing protein CRADD [Metazoa][Homo sapiens] (length=199 residues).

Site : 126- 131, Identity

sdrqinqlaq_RLGPEW_epmvlslgls

--------------------------------------------------------------------------------

[NPSA](https://npsa-prabi.ibcp.fr/cgi-bin/seq_methonseq.pl?seqid=P80192&db=unipsp&process=5be3cc137c874303&seqtype=1) [gnl|unipsp|P80192](http://www.uniprot.org/uniprot/P80192) [Eukaryota] Mitogen-activated protein kinase kinase kinase 9 [Metazoa][Homo sapiens] (length=1104 residues).

Site : 1003- 1008, Identity

prprpsanrq_RLDPWW_fvspsharst

--------------------------------------------------------------------------------

[NPSA](https://npsa-prabi.ibcp.fr/cgi-bin/seq_methonseq.pl?seqid=P80370&db=unipsp&process=5be3cc137c874304&seqtype=1) [gnl|unipsp|P80370](http://www.uniprot.org/uniprot/P80370) [Eukaryota] Protein delta homolog 1 [Metazoa][Homo sapiens] (length=383 residues).

Site : 44- 49, Identity

ngfceddnvc_RCQPGW_qgplcdqcvt

--------------------------------------------------------------------------------

[NPSA](https://npsa-prabi.ibcp.fr/cgi-bin/seq_methonseq.pl?seqid=P81408&db=unipsp&process=5be3cc137c874305&seqtype=1) [gnl|unipsp|P81408](http://www.uniprot.org/uniprot/P81408) [Eukaryota] Protein FAM189B [Metazoa][Homo sapiens] (length=668 residues).

Site : 66- 71, Identity

ssvtttesik_RSCPSW_agfslafsgv

--------------------------------------------------------------------------------

[NPSA](https://npsa-prabi.ibcp.fr/cgi-bin/seq_methonseq.pl?seqid=P86452&db=unipsp&process=5be3cc137c874306&seqtype=1) [gnl|unipsp|P86452](http://www.uniprot.org/uniprot/P86452) [Eukaryota] Zinc finger BED domain-containing protein 6 [Metazoa][Homo sapiens] (length=979 residues).

Site : 179- 184, Identity

tstlqrhlqa_RHSPHW_trankfgvas

--------------------------------------------------------------------------------

[NPSA](https://npsa-prabi.ibcp.fr/cgi-bin/seq_methonseq.pl?seqid=P98161&db=unipsp&process=5be3cc137c874307&seqtype=1) [gnl|unipsp|P98161](http://www.uniprot.org/uniprot/P98161) [Eukaryota] Polycystin-1 [Metazoa][Homo sapiens] (length=4303 residues).

Site : 3548- 3553, Identity

rtglveglrk_RLLPAW_caslahglsl

--------------------------------------------------------------------------------

[NPSA](https://npsa-prabi.ibcp.fr/cgi-bin/seq_methonseq.pl?seqid=P98164&db=unipsp&process=5be3cc137c874308&seqtype=1) [gnl|unipsp|P98164](http://www.uniprot.org/uniprot/P98164) [Eukaryota] Low-density lipoprotein receptor-related protein 2 [Metazoa][Homo sapiens] (length=4655 residues).

Site : 3691- 3696, Identity

ftefscktny_RCIPKW_avcngvddcr

--------------------------------------------------------------------------------

[NPSA](https://npsa-prabi.ibcp.fr/cgi-bin/seq_methonseq.pl?seqid=P98198&db=unipsp&process=5be3cc137c874309&seqtype=1) [gnl|unipsp|P98198](http://www.uniprot.org/uniprot/P98198) [Eukaryota] Phospholipid-transporting ATPase ID [Metazoa][Homo sapiens] (length=1209 residues).

Site : 22- 27, Identity

araqappsws_RKKPSW_gteeerrara

--------------------------------------------------------------------------------

[NPSA](https://npsa-prabi.ibcp.fr/cgi-bin/seq_methonseq.pl?seqid=Q01831&db=unipsp&process=5be3cc137c874310&seqtype=1) [gnl|unipsp|Q01831](http://www.uniprot.org/uniprot/Q01831) [Eukaryota] DNA repair protein complementing XP-C cells [Metazoa][Homo sapiens] (length=940 residues).

Site : 584- 589, Identity

sdgwvrdvtq_RYDPVW_mtvtrkcrvd

--------------------------------------------------------------------------------

[NPSA](https://npsa-prabi.ibcp.fr/cgi-bin/seq_methonseq.pl?seqid=Q01955&db=unipsp&process=5be3cc137c874311&seqtype=1) [gnl|unipsp|Q01955](http://www.uniprot.org/uniprot/Q01955) [Eukaryota] Collagen alpha-3(IV) chain [Metazoa][Homo sapiens] (length=1670 residues).

Site : 391- 396, Identity

gspgssrpgl_RGAPGW_pglkgskger

--------------------------------------------------------------------------------

[NPSA](https://npsa-prabi.ibcp.fr/cgi-bin/seq_methonseq.pl?seqid=Q01995&db=unipsp&process=5be3cc137c874312&seqtype=1) [gnl|unipsp|Q01995](http://www.uniprot.org/uniprot/Q01995) [Eukaryota] Transgelin [Metazoa][Homo sapiens] (length=201 residues).

Site : 146- 151, Identity

lavtkndghy_RGDPNW_fmkkaqehkr

--------------------------------------------------------------------------------

[NPSA](https://npsa-prabi.ibcp.fr/cgi-bin/seq_methonseq.pl?seqid=Q02161&db=unipsp&process=5be3cc137c874313&seqtype=1) [gnl|unipsp|Q02161](http://www.uniprot.org/uniprot/Q02161) [Eukaryota] Blood group Rh(D) polypeptide [Metazoa][Homo sapiens] (length=417 residues).

Site : 11- 16, Identity

msskyprsvr_RCLPLW_altleaalil

--------------------------------------------------------------------------------

[NPSA](https://npsa-prabi.ibcp.fr/cgi-bin/seq_methonseq.pl?seqid=Q02218&db=unipsp&process=5be3cc137c874314&seqtype=1) [gnl|unipsp|Q02218](http://www.uniprot.org/uniprot/Q02218) [Eukaryota] 2-oxoglutarate dehydrogenase, mitochondrial [Metazoa][Homo sapiens] (length=1023 residues).

Site : 979- 984, Identity

vkprlrttis_RAKPVW_yagrdpaaap

--------------------------------------------------------------------------------

[NPSA](https://npsa-prabi.ibcp.fr/cgi-bin/seq_methonseq.pl?seqid=Q02252&db=unipsp&process=5be3cc137c874315&seqtype=1) [gnl|unipsp|Q02252](http://www.uniprot.org/uniprot/Q02252) [Eukaryota] Methylmalonate-semialdehyde dehydrogenase [acylating], mitochondrial [Metazoa][Homo sapiens] (length=535 residues).

Site : 88- 93, Identity

emdaaiasck_RAFPAW_adtsvlsrqq

--------------------------------------------------------------------------------

[NPSA](https://npsa-prabi.ibcp.fr/cgi-bin/seq_methonseq.pl?seqid=Q02880&db=unipsp&process=5be3cc137c874316&seqtype=1) [gnl|unipsp|Q02880](http://www.uniprot.org/uniprot/Q02880) [Eukaryota] DNA topoisomerase 2-beta [Metazoa][Homo sapiens] (length=1626 residues).

Site : 856- 861, Identity

llkflyddnq_RVEPEW_yipiipmvli

--------------------------------------------------------------------------------

[NPSA](https://npsa-prabi.ibcp.fr/cgi-bin/seq_methonseq.pl?seqid=Q04721&db=unipsp&process=5be3cc137c874317&seqtype=1) [gnl|unipsp|Q04721](http://www.uniprot.org/uniprot/Q04721) [Eukaryota] Neurogenic locus notch homolog protein 2 [Metazoa][Homo sapiens] (length=2471 residues).

Site : 285- 290, Identity

cvdgvntync_RCPPQW_tgqfctedvd

--------------------------------------------------------------------------------

[NPSA](https://npsa-prabi.ibcp.fr/cgi-bin/seq_methonseq.pl?seqid=Q0IIM8&db=unipsp&process=5be3cc137c874318&seqtype=1) [gnl|unipsp|Q0IIM8](http://www.uniprot.org/uniprot/Q0IIM8) [Eukaryota] TBC1 domain family member 8B [Metazoa][Homo sapiens] (length=1120 residues).

Site : 1070- 1075, Identity

shlekdpcsf_REEPQW_sfafeqilas

--------------------------------------------------------------------------------

[NPSA](https://npsa-prabi.ibcp.fr/cgi-bin/seq_methonseq.pl?seqid=Q0P6D2&db=unipsp&process=5be3cc137c874319&seqtype=1) [gnl|unipsp|Q0P6D2](http://www.uniprot.org/uniprot/Q0P6D2) [Eukaryota] Protein FAM69C [Metazoa][Homo sapiens] (length=419 residues).

Site : 175- 180, Identity

nsslgpwwpg_RRGPRW_rgqlaslwal

--------------------------------------------------------------------------------

[NPSA](https://npsa-prabi.ibcp.fr/cgi-bin/seq_methonseq.pl?seqid=Q0VD83&db=unipsp&process=5be3cc137c874320&seqtype=1) [gnl|unipsp|Q0VD83](http://www.uniprot.org/uniprot/Q0VD83) [Eukaryota] Apolipoprotein B receptor [Metazoa][Homo sapiens] (length=1088 residues).

Site : 1009- 1014, Identity

ssqrrsrpsf_RRTPAW_eqqeeppapn

--------------------------------------------------------------------------------

[NPSA](https://npsa-prabi.ibcp.fr/cgi-bin/seq_methonseq.pl?seqid=Q0VD86&db=unipsp&process=5be3cc137c874321&seqtype=1) [gnl|unipsp|Q0VD86](http://www.uniprot.org/uniprot/Q0VD86) [Eukaryota] Protein INCA1 [Metazoa][Homo sapiens] (length=236 residues).

Site : 51- 56, Identity

gdvfwknlnq_RPTPTW_leeqhippml

--------------------------------------------------------------------------------

[NPSA](https://npsa-prabi.ibcp.fr/cgi-bin/seq_methonseq.pl?seqid=Q0VDD7&db=unipsp&process=5be3cc137c874322&seqtype=1) [gnl|unipsp|Q0VDD7](http://www.uniprot.org/uniprot/Q0VDD7) [Eukaryota] Uncharacterized protein C19orf57 [Metazoa][Homo sapiens] (length=668 residues).

Site : 660- 665, Identity

ypskgpgnip_RGDPPW_rel›

--------------------------------------------------------------------------------

[NPSA](https://npsa-prabi.ibcp.fr/cgi-bin/seq_methonseq.pl?seqid=Q11130&db=unipsp&process=5be3cc137c874323&seqtype=1) [gnl|unipsp|Q11130](http://www.uniprot.org/uniprot/Q11130) [Eukaryota] Alpha-(1,3)-fucosyltransferase 7 [Metazoa][Homo sapiens] (length=342 residues).

Site : 155- 160, Identity

rdsdifvpyg_RLEPHW_gpspplpaks

--------------------------------------------------------------------------------

[NPSA](https://npsa-prabi.ibcp.fr/cgi-bin/seq_methonseq.pl?seqid=Q12767&db=unipsp&process=5be3cc137c874324&seqtype=1) [gnl|unipsp|Q12767](http://www.uniprot.org/uniprot/Q12767) [Eukaryota] Uncharacterized protein KIAA0195 [Metazoa][Homo sapiens] (length=1356 residues).

Site : 1254- 1259, Identity

tvfisithvh_RTKPLW_rkspltnlww

--------------------------------------------------------------------------------

[NPSA](https://npsa-prabi.ibcp.fr/cgi-bin/seq_methonseq.pl?seqid=Q12794&db=unipsp&process=5be3cc137c874325&seqtype=1) [gnl|unipsp|Q12794](http://www.uniprot.org/uniprot/Q12794) [Eukaryota] Hyaluronidase-1 [Metazoa][Homo sapiens] (length=435 residues).

Site : 419- 424, Identity

qaqmavefkc_RCYPGW_qapwcerksm

--------------------------------------------------------------------------------

[NPSA](https://npsa-prabi.ibcp.fr/cgi-bin/seq_methonseq.pl?seqid=Q12931&db=unipsp&process=5be3cc137c874326&seqtype=1) [gnl|unipsp|Q12931](http://www.uniprot.org/uniprot/Q12931) [Eukaryota] Heat shock protein 75 kDa, mitochondrial [Metazoa][Homo sapiens] (length=704 residues).

Site : 46- 51, Identity

prrttaqlgp_RRNPAW_slqagrlfst

--------------------------------------------------------------------------------

[NPSA](https://npsa-prabi.ibcp.fr/cgi-bin/seq_methonseq.pl?seqid=Q12950&db=unipsp&process=5be3cc137c874327&seqtype=1) [gnl|unipsp|Q12950](http://www.uniprot.org/uniprot/Q12950) [Eukaryota] Forkhead box protein D4 [Metazoa][Homo sapiens] (length=439 residues).

Site : 143- 148, Identity

fisdrfpyyr_RKFPAW_qnsirhnlsl

--------------------------------------------------------------------------------

[NPSA](https://npsa-prabi.ibcp.fr/cgi-bin/seq_methonseq.pl?seqid=Q13136&db=unipsp&process=5be3cc137c874328&seqtype=1) [gnl|unipsp|Q13136](http://www.uniprot.org/uniprot/Q13136) [Eukaryota] Liprin-alpha-1 [Metazoa][Homo sapiens] (length=1202 residues).

Site : 1135- 1140, Identity

rfdedddksf_RRAPSW_rkkfrpkdir

--------------------------------------------------------------------------------

[NPSA](https://npsa-prabi.ibcp.fr/cgi-bin/seq_methonseq.pl?seqid=Q13219&db=unipsp&process=5be3cc137c874329&seqtype=1) [gnl|unipsp|Q13219](http://www.uniprot.org/uniprot/Q13219) [Eukaryota] Pappalysin-1 [Metazoa][Homo sapiens] (length=1627 residues).

Site : 1523- 1528, Identity

siilpmnvtv_RDIPHW_lnptrvervv

--------------------------------------------------------------------------------

[NPSA](https://npsa-prabi.ibcp.fr/cgi-bin/seq_methonseq.pl?seqid=Q13310&db=unipsp&process=5be3cc137c874330&seqtype=1) [gnl|unipsp|Q13310](http://www.uniprot.org/uniprot/Q13310) [Eukaryota] Polyadenylate-binding protein 4 [Metazoa][Homo sapiens] (length=644 residues).

Site : 432- 437, Identity

yytpnqlaqm_RPNPRW_qqggrpqgfq

--------------------------------------------------------------------------------

[NPSA](https://npsa-prabi.ibcp.fr/cgi-bin/seq_methonseq.pl?seqid=Q13315&db=unipsp&process=5be3cc137c874331&seqtype=1) [gnl|unipsp|Q13315](http://www.uniprot.org/uniprot/Q13315) [Eukaryota] Serine-protein kinase ATM [Metazoa][Homo sapiens] (length=3056 residues).

Site : 3047- 3052, Identity

qqaidpknls_RLFPGW_kawv›

--------------------------------------------------------------------------------

[NPSA](https://npsa-prabi.ibcp.fr/cgi-bin/seq_methonseq.pl?seqid=Q13395&db=unipsp&process=5be3cc137c874332&seqtype=1) [gnl|unipsp|Q13395](http://www.uniprot.org/uniprot/Q13395) [Eukaryota] Probable methyltransferase TARBP1 [Metazoa][Homo sapiens] (length=1621 residues).

Site : 772- 777, Identity

inlhlkvgwk_RGNPIW_rvisllknas

--------------------------------------------------------------------------------

[NPSA](https://npsa-prabi.ibcp.fr/cgi-bin/seq_methonseq.pl?seqid=Q13588&db=unipsp&process=5be3cc137c874333&seqtype=1) [gnl|unipsp|Q13588](http://www.uniprot.org/uniprot/Q13588) [Eukaryota] GRB2-related adapter protein [Metazoa][Homo sapiens] (length=217 residues).

Site : 190- 195, Identity

rrgdiievle_RPDPHW_wrgrscgrvg

--------------------------------------------------------------------------------

[NPSA](https://npsa-prabi.ibcp.fr/cgi-bin/seq_methonseq.pl?seqid=Q13635&db=unipsp&process=5be3cc137c874334&seqtype=1) [gnl|unipsp|Q13635](http://www.uniprot.org/uniprot/Q13635) [Eukaryota] Protein patched homolog 1 [Metazoa][Homo sapiens] (length=1447 residues).

Site : 73- 78, Identity

eqiskgkatg_RKAPLW_lrakfqrllf

--------------------------------------------------------------------------------

[NPSA](https://npsa-prabi.ibcp.fr/cgi-bin/seq_methonseq.pl?seqid=Q13642&db=unipsp&process=5be3cc137c874335&seqtype=1) [gnl|unipsp|Q13642](http://www.uniprot.org/uniprot/Q13642) [Eukaryota] Four and a half LIM domains protein 1 [Metazoa][Homo sapiens] (length=323 residues).

Site : 274- 279, Identity

nlrgrhpgge_RTCPSW_vvvlyrknrs

--------------------------------------------------------------------------------

[NPSA](https://npsa-prabi.ibcp.fr/cgi-bin/seq_methonseq.pl?seqid=Q14093&db=unipsp&process=5be3cc137c874336&seqtype=1) [gnl|unipsp|Q14093](http://www.uniprot.org/uniprot/Q14093) [Eukaryota] Cylicin-2 [Metazoa][Homo sapiens] (length=348 residues).

Site : 71- 76, Identity

iideeqlrgd_RRQPLW_myrslmrise

--------------------------------------------------------------------------------

[NPSA](https://npsa-prabi.ibcp.fr/cgi-bin/seq_methonseq.pl?seqid=Q14159&db=unipsp&process=5be3cc137c874337&seqtype=1) [gnl|unipsp|Q14159](http://www.uniprot.org/uniprot/Q14159) [Eukaryota] DNA repair-scaffolding protein [Metazoa][Homo sapiens] (length=915 residues).

Site : 361- 366, Identity

ylrgrpqdtv_RIFPPW_qkliipsgsc

--------------------------------------------------------------------------------

[NPSA](https://npsa-prabi.ibcp.fr/cgi-bin/seq_methonseq.pl?seqid=Q14162&db=unipsp&process=5be3cc137c874338&seqtype=1) [gnl|unipsp|Q14162](http://www.uniprot.org/uniprot/Q14162) [Eukaryota] Scavenger receptor class F member 1 [Metazoa][Homo sapiens] (length=830 residues).

Site : 328- 333, Identity

acepdtghcq_RCDPGW_lgprcedpcp

--------------------------------------------------------------------------------

[NPSA](https://npsa-prabi.ibcp.fr/cgi-bin/seq_methonseq.pl?seqid=Q14168&db=unipsp&process=5be3cc137c874339&seqtype=1) [gnl|unipsp|Q14168](http://www.uniprot.org/uniprot/Q14168) [Eukaryota] MAGUK p55 subfamily member 2 [Metazoa][Homo sapiens] (length=576 residues).

Site : 564- 569, Identity

relqtamekl_RTEPQW_vpvswvy›

--------------------------------------------------------------------------------

[NPSA](https://npsa-prabi.ibcp.fr/cgi-bin/seq_methonseq.pl?seqid=Q14191&db=unipsp&process=5be3cc137c874340&seqtype=1) [gnl|unipsp|Q14191](http://www.uniprot.org/uniprot/Q14191) [Eukaryota] Werner syndrome ATP-dependent helicase [Metazoa][Homo sapiens] (length=1432 residues).

Site : 13- 18, Identity

ekklettaqq_RKCPEW_mnvqnkrcav

Site : 1405- 1410, Identity

ntetssaerk_RRLPVW_fakgsdtskk

--------------------------------------------------------------------------------

[NPSA](https://npsa-prabi.ibcp.fr/cgi-bin/seq_methonseq.pl?seqid=Q14554&db=unipsp&process=5be3cc137c874341&seqtype=1) [gnl|unipsp|Q14554](http://www.uniprot.org/uniprot/Q14554) [Eukaryota] Protein disulfide-isomerase A5 [Metazoa][Homo sapiens] (length=519 residues).

Site : 3- 8, Identity

‹ma_RAGPAW_lllaiwvvlp

--------------------------------------------------------------------------------

[NPSA](https://npsa-prabi.ibcp.fr/cgi-bin/seq_methonseq.pl?seqid=Q14653&db=unipsp&process=5be3cc137c874342&seqtype=1) [gnl|unipsp|Q14653](http://www.uniprot.org/uniprot/Q14653) [Eukaryota] Interferon regulatory factor 3 [Metazoa][Homo sapiens] (length=427 residues).

Site : 236- 241, Identity

lrlvgsevgd_RTLPGW_pvtlpdpgms

--------------------------------------------------------------------------------

[NPSA](https://npsa-prabi.ibcp.fr/cgi-bin/seq_methonseq.pl?seqid=Q14714&db=unipsp&process=5be3cc137c874343&seqtype=1) [gnl|unipsp|Q14714](http://www.uniprot.org/uniprot/Q14714) [Eukaryota] Sarcospan [Metazoa][Homo sapiens] (length=243 residues).

Site : 84- 89, Identity

masisssllv_RDTPFW_agiivclvay

--------------------------------------------------------------------------------

[NPSA](https://npsa-prabi.ibcp.fr/cgi-bin/seq_methonseq.pl?seqid=Q14767&db=unipsp&process=5be3cc137c874344&seqtype=1) [gnl|unipsp|Q14767](http://www.uniprot.org/uniprot/Q14767) [Eukaryota] Latent-transforming growth factor beta-binding protein 2 [Metazoa][Homo sapiens] (length=1821 residues).

Site : 87- 92, Identity

apvaglqpve_RAQPGW_gsprrpteae

--------------------------------------------------------------------------------

[NPSA](https://npsa-prabi.ibcp.fr/cgi-bin/seq_methonseq.pl?seqid=Q14999&db=unipsp&process=5be3cc137c874345&seqtype=1) [gnl|unipsp|Q14999](http://www.uniprot.org/uniprot/Q14999) [Eukaryota] Cullin-7 [Metazoa][Homo sapiens] (length=1698 residues).

Site : 457- 462, Identity

qgavasrvlg_RALPAW_rwrpmtelya

--------------------------------------------------------------------------------

[NPSA](https://npsa-prabi.ibcp.fr/cgi-bin/seq_methonseq.pl?seqid=Q14C86&db=unipsp&process=5be3cc137c874346&seqtype=1) [gnl|unipsp|Q14C86](http://www.uniprot.org/uniprot/Q14C86) [Eukaryota] GTPase-activating protein and VPS9 domain-containing protein 1 [Metazoa][Homo sapiens] (length=1478 residues).

Site : 973- 978, Identity

rkdsddeksd_RNRPWW_rkrfvsampk

--------------------------------------------------------------------------------

[NPSA](https://npsa-prabi.ibcp.fr/cgi-bin/seq_methonseq.pl?seqid=Q15004&db=unipsp&process=5be3cc137c874347&seqtype=1) [gnl|unipsp|Q15004](http://www.uniprot.org/uniprot/Q15004) [Eukaryota] PCNA-associated factor [Metazoa][Homo sapiens] (length=111 residues).

Site : 56- 61, Identity

kyaggnpvcv_RPTPKW_qkgigeffrl

--------------------------------------------------------------------------------

[NPSA](https://npsa-prabi.ibcp.fr/cgi-bin/seq_methonseq.pl?seqid=Q15084&db=unipsp&process=5be3cc137c874348&seqtype=1) [gnl|unipsp|Q15084](http://www.uniprot.org/uniprot/Q15084) [Eukaryota] Protein disulfide-isomerase A6 [Metazoa][Homo sapiens] (length=440 residues).

Site : 60- 65, Identity

fyapwcghcq_RLTPEW_kkaatalkdv

--------------------------------------------------------------------------------

[NPSA](https://npsa-prabi.ibcp.fr/cgi-bin/seq_methonseq.pl?seqid=Q15678&db=unipsp&process=5be3cc137c874349&seqtype=1) [gnl|unipsp|Q15678](http://www.uniprot.org/uniprot/Q15678) [Eukaryota] Tyrosine-protein phosphatase non-receptor type 14 [Metazoa][Homo sapiens] (length=1187 residues).

Site : 319- 324, Identity

teqsnspppi_RRQPTW_srsslprqqp

--------------------------------------------------------------------------------

[NPSA](https://npsa-prabi.ibcp.fr/cgi-bin/seq_methonseq.pl?seqid=Q15771&db=unipsp&process=5be3cc137c874350&seqtype=1) [gnl|unipsp|Q15771](http://www.uniprot.org/uniprot/Q15771) [Eukaryota] Ras-related protein Rab-30 [Metazoa][Homo sapiens] (length=203 residues).

Site : 98- 103, Identity

tyditceesf_RCLPEW_lreieqyasn

--------------------------------------------------------------------------------

[NPSA](https://npsa-prabi.ibcp.fr/cgi-bin/seq_methonseq.pl?seqid=Q16656&db=unipsp&process=5be3cc137c874351&seqtype=1) [gnl|unipsp|Q16656](http://www.uniprot.org/uniprot/Q16656) [Eukaryota] Nuclear respiratory factor 1 [Metazoa][Homo sapiens] (length=503 residues).

Site : 219- 224, Identity

ipemlkystg_RGKPGW_gkesckpiww

--------------------------------------------------------------------------------

[NPSA](https://npsa-prabi.ibcp.fr/cgi-bin/seq_methonseq.pl?seqid=Q1AE95&db=unipsp&process=5be3cc137c874352&seqtype=1) [gnl|unipsp|Q1AE95](http://www.uniprot.org/uniprot/Q1AE95) [Eukaryota] Transmembrane protein 183B [Metazoa][Homo sapiens] (length=376 residues).

Site : 249- 254, Identity

lfwcrkivgn_RQEPMW_efnfkfkkqs

--------------------------------------------------------------------------------

[NPSA](https://npsa-prabi.ibcp.fr/cgi-bin/seq_methonseq.pl?seqid=Q2NL82&db=unipsp&process=5be3cc137c874353&seqtype=1) [gnl|unipsp|Q2NL82](http://www.uniprot.org/uniprot/Q2NL82) [Eukaryota] Pre-rRNA-processing protein TSR1 homolog [Metazoa][Homo sapiens] (length=804 residues).

Site : 772- 777, Identity

qdtvlmnlyk_RVFPKW_tydpyvpepv

--------------------------------------------------------------------------------

[NPSA](https://npsa-prabi.ibcp.fr/cgi-bin/seq_methonseq.pl?seqid=Q3KRB8&db=unipsp&process=5be3cc137c874354&seqtype=1) [gnl|unipsp|Q3KRB8](http://www.uniprot.org/uniprot/Q3KRB8) [Eukaryota] Rho GTPase-activating protein 11B [Metazoa][Homo sapiens] (length=267 residues).

Site : 230- 235, Identity

kgvyqtlswk_RYQPCW_vlmvsvllhh

--------------------------------------------------------------------------------

[NPSA](https://npsa-prabi.ibcp.fr/cgi-bin/seq_methonseq.pl?seqid=Q3SXZ7&db=unipsp&process=5be3cc137c874355&seqtype=1) [gnl|unipsp|Q3SXZ7](http://www.uniprot.org/uniprot/Q3SXZ7) [Eukaryota] Probable tubulin polyglutamylase TTLL9 [Metazoa][Homo sapiens] (length=439 residues).

Site : 59- 64, Identity

tlmntlmdvl_RHRPGW_vevkdegewd

--------------------------------------------------------------------------------

[NPSA](https://npsa-prabi.ibcp.fr/cgi-bin/seq_methonseq.pl?seqid=Q3SYB3&db=unipsp&process=5be3cc137c874356&seqtype=1) [gnl|unipsp|Q3SYB3](http://www.uniprot.org/uniprot/Q3SYB3) [Eukaryota] Forkhead box protein D4-like 6 [Metazoa][Homo sapiens] (length=417 residues).

Site : 147- 152, Identity

fisgrfpyyr_RKFPAW_qnsirhnlsl

--------------------------------------------------------------------------------

[NPSA](https://npsa-prabi.ibcp.fr/cgi-bin/seq_methonseq.pl?seqid=Q4KMQ2&db=unipsp&process=5be3cc137c874357&seqtype=1) [gnl|unipsp|Q4KMQ2](http://www.uniprot.org/uniprot/Q4KMQ2) [Eukaryota] Anoctamin-6 [Metazoa][Homo sapiens] (length=910 residues).

Site : 353- 358, Identity

gkiimcpqcd_RLCPFW_klnitcessk

--------------------------------------------------------------------------------

[NPSA](https://npsa-prabi.ibcp.fr/cgi-bin/seq_methonseq.pl?seqid=Q4UJ75&db=unipsp&process=5be3cc137c874358&seqtype=1) [gnl|unipsp|Q4UJ75](http://www.uniprot.org/uniprot/Q4UJ75) [Eukaryota] Ankyrin repeat domain-containing protein 20A4 [Metazoa][Homo sapiens] (length=823 residues).

Site : 494- 499, Identity

qslemkskta_RNTPNW_dfhnheemkg

--------------------------------------------------------------------------------

[NPSA](https://npsa-prabi.ibcp.fr/cgi-bin/seq_methonseq.pl?seqid=Q504Y2&db=unipsp&process=5be3cc137c874359&seqtype=1) [gnl|unipsp|Q504Y2](http://www.uniprot.org/uniprot/Q504Y2) [Eukaryota] Extracellular tyrosine-protein kinase PKDCC [Metazoa][Homo sapiens] (length=493 residues).

Site : 99- 104, Identity

dlapggpglp_RPRPPW_arplsdgapg

--------------------------------------------------------------------------------

[NPSA](https://npsa-prabi.ibcp.fr/cgi-bin/seq_methonseq.pl?seqid=Q59GN2&db=unipsp&process=5be3cc137c874360&seqtype=1) [gnl|unipsp|Q59GN2](http://www.uniprot.org/uniprot/Q59GN2) [Eukaryota] Putative 60S ribosomal protein L39-like 5 [Metazoa][Homo sapiens] (length=51 residues).

Site : 21- 26, Identity

qflakkqkqn_RPIPQW_irmktgnkir

--------------------------------------------------------------------------------

[NPSA](https://npsa-prabi.ibcp.fr/cgi-bin/seq_methonseq.pl?seqid=Q5BKY9&db=unipsp&process=5be3cc137c874361&seqtype=1) [gnl|unipsp|Q5BKY9](http://www.uniprot.org/uniprot/Q5BKY9) [Eukaryota] Protein FAM133B [Metazoa][Homo sapiens] (length=247 residues).

Site : 36- 41, Identity

sgptiqdyln_RPRPTW_eevkeqlekk

--------------------------------------------------------------------------------

[NPSA](https://npsa-prabi.ibcp.fr/cgi-bin/seq_methonseq.pl?seqid=Q5JTZ9&db=unipsp&process=5be3cc137c874362&seqtype=1) [gnl|unipsp|Q5JTZ9](http://www.uniprot.org/uniprot/Q5JTZ9) [Eukaryota] Alanine--tRNA ligase, mitochondrial [Metazoa][Homo sapiens] (length=985 residues).

Site : 17- 22, Identity

aaarrlrrai_RRSPAW_rglshrplss

--------------------------------------------------------------------------------

[NPSA](https://npsa-prabi.ibcp.fr/cgi-bin/seq_methonseq.pl?seqid=Q5JXM2&db=unipsp&process=5be3cc137c874363&seqtype=1) [gnl|unipsp|Q5JXM2](http://www.uniprot.org/uniprot/Q5JXM2) [Eukaryota] Methyltransferase-like protein 24 [Metazoa][Homo sapiens] (length=366 residues).

Site : 105- 110, Identity

gccaprgrpr_RKGPRW_hidlqpwags

--------------------------------------------------------------------------------

[NPSA](https://npsa-prabi.ibcp.fr/cgi-bin/seq_methonseq.pl?seqid=Q5R3I4&db=unipsp&process=5be3cc137c874364&seqtype=1) [gnl|unipsp|Q5R3I4](http://www.uniprot.org/uniprot/Q5R3I4) [Eukaryota] Tetratricopeptide repeat protein 38 [Metazoa][Homo sapiens] (length=469 residues).

Site : 167- 172, Identity

yqeqmrdsva_RIYPFW_tpdiplssyv

--------------------------------------------------------------------------------

[NPSA](https://npsa-prabi.ibcp.fr/cgi-bin/seq_methonseq.pl?seqid=Q5SR56&db=unipsp&process=5be3cc137c874365&seqtype=1) [gnl|unipsp|Q5SR56](http://www.uniprot.org/uniprot/Q5SR56) [Eukaryota] Hippocampus abundant transcript-like protein 1 [Metazoa][Homo sapiens] (length=506 residues).

Site : 128- 133, Identity

fftcfpiplm_RISPWW_yfamisvsgv

--------------------------------------------------------------------------------

[NPSA](https://npsa-prabi.ibcp.fr/cgi-bin/seq_methonseq.pl?seqid=Q5SSG8&db=unipsp&process=5be3cc137c874366&seqtype=1) [gnl|unipsp|Q5SSG8](http://www.uniprot.org/uniprot/Q5SSG8) [Eukaryota] Mucin-21 [Metazoa][Homo sapiens] (length=566 residues).

Site : 541- 546, Identity

ggnhgaphrp_RWSPNW_fwrrpvssia

--------------------------------------------------------------------------------

[NPSA](https://npsa-prabi.ibcp.fr/cgi-bin/seq_methonseq.pl?seqid=Q5SYB0&db=unipsp&process=5be3cc137c874367&seqtype=1) [gnl|unipsp|Q5SYB0](http://www.uniprot.org/uniprot/Q5SYB0) [Eukaryota] FERM and PDZ domain-containing protein 1 [Metazoa][Homo sapiens] (length=1578 residues).

Site : 1242- 1247, Identity

nhvtgqdiap_RDSPEW_vcfnpepslp

--------------------------------------------------------------------------------

[NPSA](https://npsa-prabi.ibcp.fr/cgi-bin/seq_methonseq.pl?seqid=Q5T1H1&db=unipsp&process=5be3cc137c874368&seqtype=1) [gnl|unipsp|Q5T1H1](http://www.uniprot.org/uniprot/Q5T1H1) [Eukaryota] Protein eyes shut homolog [Metazoa][Homo sapiens] (length=3165 residues).

Site : 2921- 2926, Identity

ctvngttfsc_RCLPDW_agntcnqsvs

--------------------------------------------------------------------------------

[NPSA](https://npsa-prabi.ibcp.fr/cgi-bin/seq_methonseq.pl?seqid=Q5T440&db=unipsp&process=5be3cc137c874369&seqtype=1) [gnl|unipsp|Q5T440](http://www.uniprot.org/uniprot/Q5T440) [Eukaryota] Putative transferase CAF17, mitochondrial [Metazoa][Homo sapiens] (length=356 residues).

Site : 14- 19, Identity

aallrgatpg_RGGPVW_rwrlraaprc

--------------------------------------------------------------------------------

[NPSA](https://npsa-prabi.ibcp.fr/cgi-bin/seq_methonseq.pl?seqid=Q5T5M9&db=unipsp&process=5be3cc137c874370&seqtype=1) [gnl|unipsp|Q5T5M9](http://www.uniprot.org/uniprot/Q5T5M9) [Eukaryota] Cyclin-J [Metazoa][Homo sapiens] (length=372 residues).

Site : 207- 212, Identity

acvassriil_RLSPTW_ptrlhrltay

--------------------------------------------------------------------------------

[NPSA](https://npsa-prabi.ibcp.fr/cgi-bin/seq_methonseq.pl?seqid=Q5T5N4&db=unipsp&process=5be3cc137c874371&seqtype=1) [gnl|unipsp|Q5T5N4](http://www.uniprot.org/uniprot/Q5T5N4) [Eukaryota] Uncharacterized protein C6orf118 [Metazoa][Homo sapiens] (length=469 residues).

Site : 166- 171, Identity

keekkggppg_RGPPGW_rrreelrlpd

--------------------------------------------------------------------------------

[NPSA](https://npsa-prabi.ibcp.fr/cgi-bin/seq_methonseq.pl?seqid=Q5T8R8&db=unipsp&process=5be3cc137c874372&seqtype=1) [gnl|unipsp|Q5T8R8](http://www.uniprot.org/uniprot/Q5T8R8) [Eukaryota] Uncharacterized protein C9orf66 [Metazoa][Homo sapiens] (length=295 residues).

Site : 14- 19, Identity

svarptrlpr_RLSPFW_dpatcknleg

--------------------------------------------------------------------------------

[NPSA](https://npsa-prabi.ibcp.fr/cgi-bin/seq_methonseq.pl?seqid=Q5TGY1&db=unipsp&process=5be3cc137c874373&seqtype=1) [gnl|unipsp|Q5TGY1](http://www.uniprot.org/uniprot/Q5TGY1) [Eukaryota] Transmembrane and coiled-coil domain-containing protein 4 [Metazoa][Homo sapiens] (length=634 residues).

Site : 521- 526, Identity

mdailkavgi_RTKPGW_dekglllapg

--------------------------------------------------------------------------------

[NPSA](https://npsa-prabi.ibcp.fr/cgi-bin/seq_methonseq.pl?seqid=Q5TIA1&db=unipsp&process=5be3cc137c874374&seqtype=1) [gnl|unipsp|Q5TIA1](http://www.uniprot.org/uniprot/Q5TIA1) [Eukaryota] Meiosis inhibitor protein 1 [Metazoa][Homo sapiens] (length=1274 residues).

Site : 30- 35, Identity

aallferahy_RHDPRW_llpvtprlcl

--------------------------------------------------------------------------------

[NPSA](https://npsa-prabi.ibcp.fr/cgi-bin/seq_methonseq.pl?seqid=Q5TYW2&db=unipsp&process=5be3cc137c874375&seqtype=1) [gnl|unipsp|Q5TYW2](http://www.uniprot.org/uniprot/Q5TYW2) [Eukaryota] Ankyrin repeat domain-containing protein 20A1 [Metazoa][Homo sapiens] (length=823 residues).

Site : 494- 499, Identity

qslemkskta_RNTPNW_dfhnheemkg

--------------------------------------------------------------------------------

[NPSA](https://npsa-prabi.ibcp.fr/cgi-bin/seq_methonseq.pl?seqid=Q5TZJ5&db=unipsp&process=5be3cc137c874376&seqtype=1) [gnl|unipsp|Q5TZJ5](http://www.uniprot.org/uniprot/Q5TZJ5) [Eukaryota] Spermatogenesis-associated protein 31A1 [Metazoa][Homo sapiens] (length=1347 residues).

Site : 900- 905, Identity

pawkqfqrap_RGIPSW_ndhgplkppp

--------------------------------------------------------------------------------

[NPSA](https://npsa-prabi.ibcp.fr/cgi-bin/seq_methonseq.pl?seqid=Q5U651&db=unipsp&process=5be3cc137c874377&seqtype=1) [gnl|unipsp|Q5U651](http://www.uniprot.org/uniprot/Q5U651) [Eukaryota] Ras-interacting protein 1 [Metazoa][Homo sapiens] (length=963 residues).

Site : 243- 248, Identity

erpllvqelw_RARPGW_arrfelrgre

--------------------------------------------------------------------------------

[NPSA](https://npsa-prabi.ibcp.fr/cgi-bin/seq_methonseq.pl?seqid=Q5VTT2&db=unipsp&process=5be3cc137c874378&seqtype=1) [gnl|unipsp|Q5VTT2](http://www.uniprot.org/uniprot/Q5VTT2) [Eukaryota] Uncharacterized protein C9orf135 [Metazoa][Homo sapiens] (length=229 residues).

Site : 142- 147, Identity

patgfgavfp_RHPPDW_skmcalttys

--------------------------------------------------------------------------------

[NPSA](https://npsa-prabi.ibcp.fr/cgi-bin/seq_methonseq.pl?seqid=Q5VU36&db=unipsp&process=5be3cc137c874379&seqtype=1) [gnl|unipsp|Q5VU36](http://www.uniprot.org/uniprot/Q5VU36) [Eukaryota] Spermatogenesis-associated protein 31A5 [Metazoa][Homo sapiens] (length=1347 residues).

Site : 900- 905, Identity

pawkqfqrap_RGIPSW_ndheplkppp

--------------------------------------------------------------------------------

[NPSA](https://npsa-prabi.ibcp.fr/cgi-bin/seq_methonseq.pl?seqid=Q5VU97&db=unipsp&process=5be3cc137c874380&seqtype=1) [gnl|unipsp|Q5VU97](http://www.uniprot.org/uniprot/Q5VU97) [Eukaryota] VWFA and cache domain-containing protein 1 [Metazoa][Homo sapiens] (length=1274 residues).

Site : 16- 21, Identity

eeeetavara_RRPPLW_llclvacwll

--------------------------------------------------------------------------------

[NPSA](https://npsa-prabi.ibcp.fr/cgi-bin/seq_methonseq.pl?seqid=Q5VV16&db=unipsp&process=5be3cc137c874381&seqtype=1) [gnl|unipsp|Q5VV16](http://www.uniprot.org/uniprot/Q5VV16) [Eukaryota] Forkhead box protein D4-like 5 [Metazoa][Homo sapiens] (length=416 residues).

Site : 147- 152, Identity

fisgrfpyyr_RKFPAW_qnsirhnlsl

--------------------------------------------------------------------------------

[NPSA](https://npsa-prabi.ibcp.fr/cgi-bin/seq_methonseq.pl?seqid=Q5VV67&db=unipsp&process=5be3cc137c874382&seqtype=1) [gnl|unipsp|Q5VV67](http://www.uniprot.org/uniprot/Q5VV67) [Eukaryota] Peroxisome proliferator-activated receptor gamma coactivator-related protein 1 [Metazoa][Homo sapiens] (length=1664 residues).

Site : 229- 234, Identity

spklpswrpp_RSRPRW_gqspppqqrs

--------------------------------------------------------------------------------

[NPSA](https://npsa-prabi.ibcp.fr/cgi-bin/seq_methonseq.pl?seqid=Q5VVP1&db=unipsp&process=5be3cc137c874383&seqtype=1) [gnl|unipsp|Q5VVP1](http://www.uniprot.org/uniprot/Q5VVP1) [Eukaryota] Spermatogenesis-associated protein 31A6 [Metazoa][Homo sapiens] (length=1343 residues).

Site : 896- 901, Identity

pawkqfqrap_RGIPSW_ndhgplkppp

--------------------------------------------------------------------------------

[NPSA](https://npsa-prabi.ibcp.fr/cgi-bin/seq_methonseq.pl?seqid=Q5VY80&db=unipsp&process=5be3cc137c874384&seqtype=1) [gnl|unipsp|Q5VY80](http://www.uniprot.org/uniprot/Q5VY80) [Eukaryota] Retinoic acid early transcript 1L protein [Metazoa][Homo sapiens] (length=246 residues).

Site : 44- 49, Identity

cyditvipkf_RPGPRW_cavqgqvdek

--------------------------------------------------------------------------------

[NPSA](https://npsa-prabi.ibcp.fr/cgi-bin/seq_methonseq.pl?seqid=Q5VYM1&db=unipsp&process=5be3cc137c874385&seqtype=1) [gnl|unipsp|Q5VYM1](http://www.uniprot.org/uniprot/Q5VYM1) [Eukaryota] Uncharacterized protein C9orf131 [Metazoa][Homo sapiens] (length=1079 residues).

Site : 409- 414, Identity

qressledps_RYKPQW_ecrensgnlw

--------------------------------------------------------------------------------

[NPSA](https://npsa-prabi.ibcp.fr/cgi-bin/seq_methonseq.pl?seqid=Q5VYP0&db=unipsp&process=5be3cc137c874386&seqtype=1) [gnl|unipsp|Q5VYP0](http://www.uniprot.org/uniprot/Q5VYP0) [Eukaryota] Spermatogenesis-associated protein 31A3 [Metazoa][Homo sapiens] (length=1347 residues).

Site : 900- 905, Identity

pawkqfqrap_RGIPSW_ndheplkppp

--------------------------------------------------------------------------------

[NPSA](https://npsa-prabi.ibcp.fr/cgi-bin/seq_methonseq.pl?seqid=Q63HN8&db=unipsp&process=5be3cc137c874387&seqtype=1) [gnl|unipsp|Q63HN8](http://www.uniprot.org/uniprot/Q63HN8) [Eukaryota] E3 ubiquitin-protein ligase RNF213 [Metazoa][Homo sapiens] (length=5207 residues).

Site : 5151- 5156, Identity

lknpqtqtee_RFRPQW_slrdtlvsym

--------------------------------------------------------------------------------

[NPSA](https://npsa-prabi.ibcp.fr/cgi-bin/seq_methonseq.pl?seqid=Q658N2&db=unipsp&process=5be3cc137c874388&seqtype=1) [gnl|unipsp|Q658N2](http://www.uniprot.org/uniprot/Q658N2) [Eukaryota] WSC domain-containing protein 1 [Metazoa][Homo sapiens] (length=575 residues).

Site : 96- 101, Identity

dmlqspltrp_RPGPRW_lrsrnselrq

--------------------------------------------------------------------------------

[NPSA](https://npsa-prabi.ibcp.fr/cgi-bin/seq_methonseq.pl?seqid=Q658P3&db=unipsp&process=5be3cc137c874389&seqtype=1) [gnl|unipsp|Q658P3](http://www.uniprot.org/uniprot/Q658P3) [Eukaryota] Metalloreductase STEAP3 [Metazoa][Homo sapiens] (length=488 residues).

Site : 207- 212, Identity

saweveampl_RLLPAW_kvptllalgl

--------------------------------------------------------------------------------

[NPSA](https://npsa-prabi.ibcp.fr/cgi-bin/seq_methonseq.pl?seqid=Q687X5&db=unipsp&process=5be3cc137c874390&seqtype=1) [gnl|unipsp|Q687X5](http://www.uniprot.org/uniprot/Q687X5) [Eukaryota] Metalloreductase STEAP4 [Metazoa][Homo sapiens] (length=459 residues).

Site : 277- 282, Identity

ilqlyrgtky_RRFPDW_ldhwmlcrkq

--------------------------------------------------------------------------------

[NPSA](https://npsa-prabi.ibcp.fr/cgi-bin/seq_methonseq.pl?seqid=Q68DL7&db=unipsp&process=5be3cc137c874391&seqtype=1) [gnl|unipsp|Q68DL7](http://www.uniprot.org/uniprot/Q68DL7) [Eukaryota] Uncharacterized protein C18orf63 [Metazoa][Homo sapiens] (length=685 residues).

Site : 113- 118, Identity

qnclsysfma_RLAPAW_nrtghlliqg

--------------------------------------------------------------------------------

[NPSA](https://npsa-prabi.ibcp.fr/cgi-bin/seq_methonseq.pl?seqid=Q68DN1&db=unipsp&process=5be3cc137c874392&seqtype=1) [gnl|unipsp|Q68DN1](http://www.uniprot.org/uniprot/Q68DN1) [Eukaryota] Uncharacterized protein C2orf16 [Metazoa][Homo sapiens] (length=1984 residues).

Site : 1558- 1563, Identity

rtprgpsert_RHNPSW_rnhrspsers

--------------------------------------------------------------------------------

[NPSA](https://npsa-prabi.ibcp.fr/cgi-bin/seq_methonseq.pl?seqid=Q6H3X3&db=unipsp&process=5be3cc137c874393&seqtype=1) [gnl|unipsp|Q6H3X3](http://www.uniprot.org/uniprot/Q6H3X3) [Eukaryota] Retinoic acid early transcript 1G protein [Metazoa][Homo sapiens] (length=334 residues).

Site : 44- 49, Identity

cyditvipkf_RPGPRW_cavqgqvdek

--------------------------------------------------------------------------------

[NPSA](https://npsa-prabi.ibcp.fr/cgi-bin/seq_methonseq.pl?seqid=Q6IQ21&db=unipsp&process=5be3cc137c874394&seqtype=1) [gnl|unipsp|Q6IQ21](http://www.uniprot.org/uniprot/Q6IQ21) [Eukaryota] Zinc finger protein 770 [Metazoa][Homo sapiens] (length=691 residues).

Site : 663- 668, Identity

fecsvcgktf_RQAPHW_krhqlthfke

--------------------------------------------------------------------------------

[NPSA](https://npsa-prabi.ibcp.fr/cgi-bin/seq_methonseq.pl?seqid=Q6NSJ5&db=unipsp&process=5be3cc137c874395&seqtype=1) [gnl|unipsp|Q6NSJ5](http://www.uniprot.org/uniprot/Q6NSJ5) [Eukaryota] Volume-regulated anion channel subunit LRRC8E [Metazoa][Homo sapiens] (length=796 residues).

Site : 497- 502, Identity

kvmrvkceel_REVPLW_vfglrgleel

--------------------------------------------------------------------------------

[NPSA](https://npsa-prabi.ibcp.fr/cgi-bin/seq_methonseq.pl?seqid=Q6NUN7&db=unipsp&process=5be3cc137c874396&seqtype=1) [gnl|unipsp|Q6NUN7](http://www.uniprot.org/uniprot/Q6NUN7) [Eukaryota] Uncharacterized protein C11orf63 [Metazoa][Homo sapiens] (length=778 residues).

Site : 127- 132, Identity

qpiedkysdl_RYDPNW_kskkeegqll

--------------------------------------------------------------------------------

[NPSA](https://npsa-prabi.ibcp.fr/cgi-bin/seq_methonseq.pl?seqid=Q6NV74&db=unipsp&process=5be3cc137c874397&seqtype=1) [gnl|unipsp|Q6NV74](http://www.uniprot.org/uniprot/Q6NV74) [Eukaryota] Uncharacterized protein KIAA1211-like [Metazoa][Homo sapiens] (length=962 residues).

Site : 602- 607, Identity

erasgrlpla_RSGPVW_rseaalddlq

--------------------------------------------------------------------------------

[NPSA](https://npsa-prabi.ibcp.fr/cgi-bin/seq_methonseq.pl?seqid=Q6P1K2&db=unipsp&process=5be3cc137c874398&seqtype=1) [gnl|unipsp|Q6P1K2](http://www.uniprot.org/uniprot/Q6P1K2) [Eukaryota] Polyamine-modulated factor 1 [Metazoa][Homo sapiens] (length=205 residues).

Site : 118- 123, Identity

ldkiveegkv_RKEPAW_rpsgipekdl

--------------------------------------------------------------------------------

[NPSA](https://npsa-prabi.ibcp.fr/cgi-bin/seq_methonseq.pl?seqid=Q6P2Q9&db=unipsp&process=5be3cc137c874399&seqtype=1) [gnl|unipsp|Q6P2Q9](http://www.uniprot.org/uniprot/Q6P2Q9) [Eukaryota] Pre-mRNA-processing-splicing factor 8 [Metazoa][Homo sapiens] (length=2335 residues).

Site : 934- 939, Identity

qylwyeadkr_RLFPPW_ikpadteppp

--------------------------------------------------------------------------------

[NPSA](https://npsa-prabi.ibcp.fr/cgi-bin/seq_methonseq.pl?seqid=Q6P474&db=unipsp&process=5be3cc137c874400&seqtype=1) [gnl|unipsp|Q6P474](http://www.uniprot.org/uniprot/Q6P474) [Eukaryota] Putative pyridoxal-dependent decarboxylase domain-containing protein 2 [Metazoa][Homo sapiens] (length=469 residues).

Site : 336- 341, Identity

lisnkptdkl_RALPLW_lslqylgldg

--------------------------------------------------------------------------------

[NPSA](https://npsa-prabi.ibcp.fr/cgi-bin/seq_methonseq.pl?seqid=Q6P4Q7&db=unipsp&process=5be3cc137c874401&seqtype=1) [gnl|unipsp|Q6P4Q7](http://www.uniprot.org/uniprot/Q6P4Q7) [Eukaryota] Metal transporter CNNM4 [Metazoa][Homo sapiens] (length=775 residues).

Site : 178- 183, Identity

sllfmveepg_RFLPLW_lhillitvll

--------------------------------------------------------------------------------

[NPSA](https://npsa-prabi.ibcp.fr/cgi-bin/seq_methonseq.pl?seqid=Q6P996&db=unipsp&process=5be3cc137c874402&seqtype=1) [gnl|unipsp|Q6P996](http://www.uniprot.org/uniprot/Q6P996) [Eukaryota] Pyridoxal-dependent decarboxylase domain-containing protein 1 [Metazoa][Homo sapiens] (length=788 residues).

Site : 337- 342, Identity

ltsnkptdkl_RALPLW_lslqylgldg

--------------------------------------------------------------------------------

[NPSA](https://npsa-prabi.ibcp.fr/cgi-bin/seq_methonseq.pl?seqid=Q6PRD1&db=unipsp&process=5be3cc137c874403&seqtype=1) [gnl|unipsp|Q6PRD1](http://www.uniprot.org/uniprot/Q6PRD1) [Eukaryota] Probable G-protein coupled receptor 179 [Metazoa][Homo sapiens] (length=2367 residues).

Site : 240- 245, Identity

sppflecqeg_RLRPGW_litlsatfyg

--------------------------------------------------------------------------------

[NPSA](https://npsa-prabi.ibcp.fr/cgi-bin/seq_methonseq.pl?seqid=Q6UUV7&db=unipsp&process=5be3cc137c874404&seqtype=1) [gnl|unipsp|Q6UUV7](http://www.uniprot.org/uniprot/Q6UUV7) [Eukaryota] CREB-regulated transcription coactivator 3 [Metazoa][Homo sapiens] (length=619 residues).

Site : 138- 143, Identity

ssqpldeswp_RQQPPW_kdekhpgfrl

--------------------------------------------------------------------------------

[NPSA](https://npsa-prabi.ibcp.fr/cgi-bin/seq_methonseq.pl?seqid=Q6UVM3&db=unipsp&process=5be3cc137c874405&seqtype=1) [gnl|unipsp|Q6UVM3](http://www.uniprot.org/uniprot/Q6UVM3) [Eukaryota] Potassium channel subfamily T member 2 [Metazoa][Homo sapiens] (length=1135 residues).

Site : 100- 105, Identity

newshifwvn_RSLPLW_glqvsvalis

--------------------------------------------------------------------------------

[NPSA](https://npsa-prabi.ibcp.fr/cgi-bin/seq_methonseq.pl?seqid=Q6UWB1&db=unipsp&process=5be3cc137c874406&seqtype=1) [gnl|unipsp|Q6UWB1](http://www.uniprot.org/uniprot/Q6UWB1) [Eukaryota] Interleukin-27 receptor subunit alpha [Metazoa][Homo sapiens] (length=636 residues).

Site : 5- 10, Identity

‹mrgg_RGAPFW_lwplpklall

--------------------------------------------------------------------------------

[NPSA](https://npsa-prabi.ibcp.fr/cgi-bin/seq_methonseq.pl?seqid=Q6UWL6&db=unipsp&process=5be3cc137c874407&seqtype=1) [gnl|unipsp|Q6UWL6](http://www.uniprot.org/uniprot/Q6UWL6) [Eukaryota] Kin of IRRE-like protein 2 [Metazoa][Homo sapiens] (length=708 residues).

Site : 67- 72, Identity

tksglalggq_RDLPGW_srywisgnaa

--------------------------------------------------------------------------------

[NPSA](https://npsa-prabi.ibcp.fr/cgi-bin/seq_methonseq.pl?seqid=Q6UWN0&db=unipsp&process=5be3cc137c874408&seqtype=1) [gnl|unipsp|Q6UWN0](http://www.uniprot.org/uniprot/Q6UWN0) [Eukaryota] Ly6/PLAUR domain-containing protein 4 [Metazoa][Homo sapiens] (length=246 residues).

Site : 229- 234, Identity

ksqivgaass_RQDPAW_gvvlgllfaf

--------------------------------------------------------------------------------

[NPSA](https://npsa-prabi.ibcp.fr/cgi-bin/seq_methonseq.pl?seqid=Q6UX07&db=unipsp&process=5be3cc137c874409&seqtype=1) [gnl|unipsp|Q6UX07](http://www.uniprot.org/uniprot/Q6UX07) [Eukaryota] Dehydrogenase/reductase SDR family member 13 [Metazoa][Homo sapiens] (length=377 residues).

Site : 237- 242, Identity

pgpvnselfl_RHVPGW_lrpllrplaw

--------------------------------------------------------------------------------

[NPSA](https://npsa-prabi.ibcp.fr/cgi-bin/seq_methonseq.pl?seqid=Q6UXA7&db=unipsp&process=5be3cc137c874410&seqtype=1) [gnl|unipsp|Q6UXA7](http://www.uniprot.org/uniprot/Q6UXA7) [Eukaryota] Uncharacterized protein C6orf15 [Metazoa][Homo sapiens] (length=325 residues).

Site : 97- 102, Identity

fppaggsavq_RWPPSW_glpamdswpp

--------------------------------------------------------------------------------

[NPSA](https://npsa-prabi.ibcp.fr/cgi-bin/seq_methonseq.pl?seqid=Q6UXC1&db=unipsp&process=5be3cc137c874411&seqtype=1) [gnl|unipsp|Q6UXC1](http://www.uniprot.org/uniprot/Q6UXC1) [Eukaryota] Apical endosomal glycoprotein [Metazoa][Homo sapiens] (length=1216 residues).

Site : 803- 808, Identity

gtmalddvav_RPGPCW_apnycsfeds

--------------------------------------------------------------------------------

[NPSA](https://npsa-prabi.ibcp.fr/cgi-bin/seq_methonseq.pl?seqid=Q6UXX5&db=unipsp&process=5be3cc137c874412&seqtype=1) [gnl|unipsp|Q6UXX5](http://www.uniprot.org/uniprot/Q6UXX5) [Eukaryota] Inter-alpha-trypsin inhibitor heavy chain H6 [Metazoa][Homo sapiens] (length=1313 residues).

Site : 1284- 1289, Identity

lgkrllkdsp_RLLPRW_ascwlvkrsh

--------------------------------------------------------------------------------

[NPSA](https://npsa-prabi.ibcp.fr/cgi-bin/seq_methonseq.pl?seqid=Q6UY11&db=unipsp&process=5be3cc137c874413&seqtype=1) [gnl|unipsp|Q6UY11](http://www.uniprot.org/uniprot/Q6UY11) [Eukaryota] Protein delta homolog 2 [Metazoa][Homo sapiens] (length=383 residues).

Site : 47- 52, Identity

hgccapdgsc_RCDPGW_eglhcercvr

--------------------------------------------------------------------------------

[NPSA](https://npsa-prabi.ibcp.fr/cgi-bin/seq_methonseq.pl?seqid=Q6VB84&db=unipsp&process=5be3cc137c874414&seqtype=1) [gnl|unipsp|Q6VB84](http://www.uniprot.org/uniprot/Q6VB84) [Eukaryota] Forkhead box protein D4-like 3 [Metazoa][Homo sapiens] (length=417 residues).

Site : 147- 152, Identity

fisgrfpyyr_RKFPAW_qnsirhnlsl

--------------------------------------------------------------------------------

[NPSA](https://npsa-prabi.ibcp.fr/cgi-bin/seq_methonseq.pl?seqid=Q6VB85&db=unipsp&process=5be3cc137c874415&seqtype=1) [gnl|unipsp|Q6VB85](http://www.uniprot.org/uniprot/Q6VB85) [Eukaryota] Forkhead box protein D4-like 2 [Metazoa][Homo sapiens] (length=416 residues).

Site : 147- 152, Identity

fisgrfpyyr_RKFPAW_qnsirhnlsl

--------------------------------------------------------------------------------

[NPSA](https://npsa-prabi.ibcp.fr/cgi-bin/seq_methonseq.pl?seqid=Q6WRI0&db=unipsp&process=5be3cc137c874416&seqtype=1) [gnl|unipsp|Q6WRI0](http://www.uniprot.org/uniprot/Q6WRI0) [Eukaryota] Immunoglobulin superfamily member 10 [Metazoa][Homo sapiens] (length=2623 residues).

Site : 423- 428, Identity

diftnieadl_RADPSW_lmqdqislql

--------------------------------------------------------------------------------

[NPSA](https://npsa-prabi.ibcp.fr/cgi-bin/seq_methonseq.pl?seqid=Q6ZMN8&db=unipsp&process=5be3cc137c874417&seqtype=1) [gnl|unipsp|Q6ZMN8](http://www.uniprot.org/uniprot/Q6ZMN8) [Eukaryota] Cyclin-I2 [Metazoa][Homo sapiens] (length=369 residues).

Site : 318- 323, Identity

alviitlele_RLMPGW_capisdllkk

--------------------------------------------------------------------------------

[NPSA](https://npsa-prabi.ibcp.fr/cgi-bin/seq_methonseq.pl?seqid=Q6ZMY9&db=unipsp&process=5be3cc137c874418&seqtype=1) [gnl|unipsp|Q6ZMY9](http://www.uniprot.org/uniprot/Q6ZMY9) [Eukaryota] Zinc finger protein 517 [Metazoa][Homo sapiens] (length=492 residues).

Site : 485- 490, Identity

grepgedteg_RRAPCW_as›

--------------------------------------------------------------------------------

[NPSA](https://npsa-prabi.ibcp.fr/cgi-bin/seq_methonseq.pl?seqid=Q6ZNJ1&db=unipsp&process=5be3cc137c874419&seqtype=1) [gnl|unipsp|Q6ZNJ1](http://www.uniprot.org/uniprot/Q6ZNJ1) [Eukaryota] Neurobeachin-like protein 2 [Metazoa][Homo sapiens] (length=2754 residues).

Site : 985- 990, Identity

qgpaiigall_RKVPSW_amdmnvlmsa

--------------------------------------------------------------------------------

[NPSA](https://npsa-prabi.ibcp.fr/cgi-bin/seq_methonseq.pl?seqid=Q6ZQQ6&db=unipsp&process=5be3cc137c874420&seqtype=1) [gnl|unipsp|Q6ZQQ6](http://www.uniprot.org/uniprot/Q6ZQQ6) [Eukaryota] WD repeat-containing protein 87 [Metazoa][Homo sapiens] (length=2873 residues).

Site : 5- 10, Identity

‹mssp_RLIPLW_kdlklllndt

--------------------------------------------------------------------------------

[NPSA](https://npsa-prabi.ibcp.fr/cgi-bin/seq_methonseq.pl?seqid=Q6ZR54&db=unipsp&process=5be3cc137c874421&seqtype=1) [gnl|unipsp|Q6ZR54](http://www.uniprot.org/uniprot/Q6ZR54) [Eukaryota] Putative uncharacterized protein FLJ46641 [Metazoa][Homo sapiens] (length=194 residues).

Site : 22- 27, Identity

fadvsqqirv_RNRPSW_acrrggplgt

--------------------------------------------------------------------------------

[NPSA](https://npsa-prabi.ibcp.fr/cgi-bin/seq_methonseq.pl?seqid=Q6ZRI0&db=unipsp&process=5be3cc137c874422&seqtype=1) [gnl|unipsp|Q6ZRI0](http://www.uniprot.org/uniprot/Q6ZRI0) [Eukaryota] Otogelin [Metazoa][Homo sapiens] (length=2925 residues).

Site : 2101- 2106, Identity

lglavrvggd_RCCPLW_ecacrcsifp

--------------------------------------------------------------------------------

[NPSA](https://npsa-prabi.ibcp.fr/cgi-bin/seq_methonseq.pl?seqid=Q6ZSA8&db=unipsp&process=5be3cc137c874423&seqtype=1) [gnl|unipsp|Q6ZSA8](http://www.uniprot.org/uniprot/Q6ZSA8) [Eukaryota] Putative uncharacterized protein FLJ45684 [Metazoa][Homo sapiens] (length=131 residues).

Site : 84- 89, Identity

rapsgllpqr_RGGPSW_wppsgvargg

Site : 97- 102, Identity

pswwppsgva_RGGPSW_wppsgvvrgg

--------------------------------------------------------------------------------

[NPSA](https://npsa-prabi.ibcp.fr/cgi-bin/seq_methonseq.pl?seqid=Q6ZUT9&db=unipsp&process=5be3cc137c874424&seqtype=1) [gnl|unipsp|Q6ZUT9](http://www.uniprot.org/uniprot/Q6ZUT9) [Eukaryota] DENN domain-containing protein 5B [Metazoa][Homo sapiens] (length=1274 residues).

Site : 1216- 1221, Identity

qilvclgtrd_RLLPQW_ipllaecpai

--------------------------------------------------------------------------------

[NPSA](https://npsa-prabi.ibcp.fr/cgi-bin/seq_methonseq.pl?seqid=Q75N90&db=unipsp&process=5be3cc137c874425&seqtype=1) [gnl|unipsp|Q75N90](http://www.uniprot.org/uniprot/Q75N90) [Eukaryota] Fibrillin-3 [Metazoa][Homo sapiens] (length=2809 residues).

Site : 1307- 1312, Identity

clnipgsfsc_RCLPGW_vgdgfechdl

--------------------------------------------------------------------------------

[NPSA](https://npsa-prabi.ibcp.fr/cgi-bin/seq_methonseq.pl?seqid=Q76N32&db=unipsp&process=5be3cc137c874426&seqtype=1) [gnl|unipsp|Q76N32](http://www.uniprot.org/uniprot/Q76N32) [Eukaryota] Centrosomal protein of 68 kDa [Metazoa][Homo sapiens] (length=757 residues).

Site : 288- 293, Identity

lpdslppspd_RHSPLW_npnkeyedll

--------------------------------------------------------------------------------

[NPSA](https://npsa-prabi.ibcp.fr/cgi-bin/seq_methonseq.pl?seqid=Q76NI1&db=unipsp&process=5be3cc137c874427&seqtype=1) [gnl|unipsp|Q76NI1](http://www.uniprot.org/uniprot/Q76NI1) [Eukaryota] Protein very KIND [Metazoa][Homo sapiens] (length=1749 residues).

Site : 1597- 1602, Identity

ilsglehlav_RQSPAW_rilpakiaev

--------------------------------------------------------------------------------

[NPSA](https://npsa-prabi.ibcp.fr/cgi-bin/seq_methonseq.pl?seqid=Q7L211&db=unipsp&process=5be3cc137c874428&seqtype=1) [gnl|unipsp|Q7L211](http://www.uniprot.org/uniprot/Q7L211) [Eukaryota] Alpha/beta hydrolase domain-containing protein 13 [Metazoa][Homo sapiens] (length=337 residues).

Site : 236- 241, Identity

astlfsffpm_RYLPLW_cyknkflsyr

--------------------------------------------------------------------------------

[NPSA](https://npsa-prabi.ibcp.fr/cgi-bin/seq_methonseq.pl?seqid=Q7L2E3&db=unipsp&process=5be3cc137c874429&seqtype=1) [gnl|unipsp|Q7L2E3](http://www.uniprot.org/uniprot/Q7L2E3) [Eukaryota] Putative ATP-dependent RNA helicase DHX30 [Metazoa][Homo sapiens] (length=1194 residues).

Site : 423- 428, Identity

lsqsllelwr_RRGPVW_qeapqlpvdp

--------------------------------------------------------------------------------

[NPSA](https://npsa-prabi.ibcp.fr/cgi-bin/seq_methonseq.pl?seqid=Q7L2K0&db=unipsp&process=5be3cc137c874430&seqtype=1) [gnl|unipsp|Q7L2K0](http://www.uniprot.org/uniprot/Q7L2K0) [Eukaryota] Uncharacterized protein C16orf59 [Metazoa][Homo sapiens] (length=433 residues).

Site : 346- 351, Identity

ppgaspscgg_RAEPAW_spqllvysst

--------------------------------------------------------------------------------

[NPSA](https://npsa-prabi.ibcp.fr/cgi-bin/seq_methonseq.pl?seqid=Q7L8J4&db=unipsp&process=5be3cc137c874431&seqtype=1) [gnl|unipsp|Q7L8J4](http://www.uniprot.org/uniprot/Q7L8J4) [Eukaryota] SH3 domain-binding protein 5-like [Metazoa][Homo sapiens] (length=393 residues).

Site : 164- 169, Identity

aeqgvmadkn_RLDPTW_qemlnhatck

--------------------------------------------------------------------------------

[NPSA](https://npsa-prabi.ibcp.fr/cgi-bin/seq_methonseq.pl?seqid=Q7RTU9&db=unipsp&process=5be3cc137c874432&seqtype=1) [gnl|unipsp|Q7RTU9](http://www.uniprot.org/uniprot/Q7RTU9) [Eukaryota] Stereocilin [Metazoa][Homo sapiens] (length=1775 residues).

Site : 101- 106, Identity

lrlhdflvtl_RGSPDW_epmlgllgdm

--------------------------------------------------------------------------------

[NPSA](https://npsa-prabi.ibcp.fr/cgi-bin/seq_methonseq.pl?seqid=Q7Z3H4&db=unipsp&process=5be3cc137c874433&seqtype=1) [gnl|unipsp|Q7Z3H4](http://www.uniprot.org/uniprot/Q7Z3H4) [Eukaryota] Sterile alpha motif domain-containing protein 7 [Metazoa][Homo sapiens] (length=446 residues).

Site : 67- 72, Identity

ntnmanvlss_RIYPGW_gilppesika

--------------------------------------------------------------------------------

[NPSA](https://npsa-prabi.ibcp.fr/cgi-bin/seq_methonseq.pl?seqid=Q7Z5Y6&db=unipsp&process=5be3cc137c874434&seqtype=1) [gnl|unipsp|Q7Z5Y6](http://www.uniprot.org/uniprot/Q7Z5Y6) [Eukaryota] Bone morphogenetic protein 8A [Metazoa][Homo sapiens] (length=402 residues).

Site : 4- 9, Identity

‹maa_RPGPLW_llgltlcalg

--------------------------------------------------------------------------------

[NPSA](https://npsa-prabi.ibcp.fr/cgi-bin/seq_methonseq.pl?seqid=Q7Z7A4&db=unipsp&process=5be3cc137c874435&seqtype=1) [gnl|unipsp|Q7Z7A4](http://www.uniprot.org/uniprot/Q7Z7A4) [Eukaryota] PX domain-containing protein kinase-like protein [Metazoa][Homo sapiens] (length=578 residues).

Site : 141- 146, Identity

ialqqvsmff_RSEPKW_evveplkdig

--------------------------------------------------------------------------------

[NPSA](https://npsa-prabi.ibcp.fr/cgi-bin/seq_methonseq.pl?seqid=Q7Z7L8&db=unipsp&process=5be3cc137c874436&seqtype=1) [gnl|unipsp|Q7Z7L8](http://www.uniprot.org/uniprot/Q7Z7L8) [Eukaryota] Uncharacterized protein C11orf96 [Metazoa][Homo sapiens] (length=435 residues).

Site : 110- 115, Identity

kgagegdlrp_RGQPGW_crlgdprrds

--------------------------------------------------------------------------------

[NPSA](https://npsa-prabi.ibcp.fr/cgi-bin/seq_methonseq.pl?seqid=Q7Z7M8&db=unipsp&process=5be3cc137c874437&seqtype=1) [gnl|unipsp|Q7Z7M8](http://www.uniprot.org/uniprot/Q7Z7M8) [Eukaryota] UDP-GlcNAc:betaGal beta-1,3-N-acetylglucosaminyltransferase 8 [Metazoa][Homo sapiens] (length=397 residues).

Site : 125- 130, Identity

lrrfllsaac_RSFPQW_lpggggsqvs

--------------------------------------------------------------------------------

[NPSA](https://npsa-prabi.ibcp.fr/cgi-bin/seq_methonseq.pl?seqid=Q7Z7N9&db=unipsp&process=5be3cc137c874438&seqtype=1) [gnl|unipsp|Q7Z7N9](http://www.uniprot.org/uniprot/Q7Z7N9) [Eukaryota] Transmembrane protein 179B [Metazoa][Homo sapiens] (length=219 residues).

Site : 199- 204, Identity

seatpyrple_RGDPEW_ssetdalvgs

--------------------------------------------------------------------------------

[NPSA](https://npsa-prabi.ibcp.fr/cgi-bin/seq_methonseq.pl?seqid=Q86T26&db=unipsp&process=5be3cc137c874439&seqtype=1) [gnl|unipsp|Q86T26](http://www.uniprot.org/uniprot/Q86T26) [Eukaryota] Transmembrane protease serine 11B [Metazoa][Homo sapiens] (length=416 residues).

Site : 10- 15, Identity

‹myrhgissq_RSWPLW_ttififlgva

--------------------------------------------------------------------------------

[NPSA](https://npsa-prabi.ibcp.fr/cgi-bin/seq_methonseq.pl?seqid=Q86U02&db=unipsp&process=5be3cc137c874440&seqtype=1) [gnl|unipsp|Q86U02](http://www.uniprot.org/uniprot/Q86U02) [Eukaryota] Putative uncharacterized protein encoded by LINC00596 [Metazoa][Homo sapiens] (length=117 residues).

Site : 108- 113, Identity

lanfyifsgd_RVSPCW_pdws›

--------------------------------------------------------------------------------

[NPSA](https://npsa-prabi.ibcp.fr/cgi-bin/seq_methonseq.pl?seqid=Q86UK5&db=unipsp&process=5be3cc137c874441&seqtype=1) [gnl|unipsp|Q86UK5](http://www.uniprot.org/uniprot/Q86UK5) [Eukaryota] Limbin [Metazoa][Homo sapiens] (length=1308 residues).

Site : 7- 12, Identity

‹mdpsgs_RGRPTW_vlaggllava

--------------------------------------------------------------------------------

[NPSA](https://npsa-prabi.ibcp.fr/cgi-bin/seq_methonseq.pl?seqid=Q86UN3&db=unipsp&process=5be3cc137c874442&seqtype=1) [gnl|unipsp|Q86UN3](http://www.uniprot.org/uniprot/Q86UN3) [Eukaryota] Reticulon-4 receptor-like 2 [Metazoa][Homo sapiens] (length=420 residues).

Site : 268- 273, Identity

lnanpwacdc_RARPLW_awfqrarvss

--------------------------------------------------------------------------------

[NPSA](https://npsa-prabi.ibcp.fr/cgi-bin/seq_methonseq.pl?seqid=Q86UY5&db=unipsp&process=5be3cc137c874443&seqtype=1) [gnl|unipsp|Q86UY5](http://www.uniprot.org/uniprot/Q86UY5) [Eukaryota] Protein FAM83A [Metazoa][Homo sapiens] (length=434 residues).

Site : 419- 424, Identity

lqleqlglvp_RLTPTW_rpflqasphf

--------------------------------------------------------------------------------

[NPSA](https://npsa-prabi.ibcp.fr/cgi-bin/seq_methonseq.pl?seqid=Q86V40&db=unipsp&process=5be3cc137c874444&seqtype=1) [gnl|unipsp|Q86V40](http://www.uniprot.org/uniprot/Q86V40) [Eukaryota] Metalloprotease TIKI1 [Metazoa][Homo sapiens] (length=505 residues).

Site : 162- 167, Identity

lfnaiagnwe_RKRPVW_vmlmvnslte

--------------------------------------------------------------------------------

[NPSA](https://npsa-prabi.ibcp.fr/cgi-bin/seq_methonseq.pl?seqid=Q86VR8&db=unipsp&process=5be3cc137c874445&seqtype=1) [gnl|unipsp|Q86VR8](http://www.uniprot.org/uniprot/Q86VR8) [Eukaryota] Four-jointed box protein 1 [Metazoa][Homo sapiens] (length=437 residues).

Site : 98- 103, Identity

agadgpprqs_RSEPRW_hvsarqprpe

--------------------------------------------------------------------------------

[NPSA](https://npsa-prabi.ibcp.fr/cgi-bin/seq_methonseq.pl?seqid=Q86VU5&db=unipsp&process=5be3cc137c874446&seqtype=1) [gnl|unipsp|Q86VU5](http://www.uniprot.org/uniprot/Q86VU5) [Eukaryota] Catechol O-methyltransferase domain-containing protein 1 [Metazoa][Homo sapiens] (length=262 residues).

Site : 33- 38, Identity

aafatglflg_RRCPPW_rgrreqcllp

--------------------------------------------------------------------------------

[NPSA](https://npsa-prabi.ibcp.fr/cgi-bin/seq_methonseq.pl?seqid=Q86VY9&db=unipsp&process=5be3cc137c874447&seqtype=1) [gnl|unipsp|Q86VY9](http://www.uniprot.org/uniprot/Q86VY9) [Eukaryota] Transmembrane protein 200A [Metazoa][Homo sapiens] (length=491 residues).

Site : 414- 419, Identity

pstltvqaeq_RKHPSW_prldrnnskg

--------------------------------------------------------------------------------

[NPSA](https://npsa-prabi.ibcp.fr/cgi-bin/seq_methonseq.pl?seqid=Q86WK7&db=unipsp&process=5be3cc137c874448&seqtype=1) [gnl|unipsp|Q86WK7](http://www.uniprot.org/uniprot/Q86WK7) [Eukaryota] Amphoterin-induced protein 3 [Metazoa][Homo sapiens] (length=504 residues).

Site : 75- 80, Identity

dldlshnalq_RLRPGW_laplfqlral

--------------------------------------------------------------------------------

[NPSA](https://npsa-prabi.ibcp.fr/cgi-bin/seq_methonseq.pl?seqid=Q86YB8&db=unipsp&process=5be3cc137c874449&seqtype=1) [gnl|unipsp|Q86YB8](http://www.uniprot.org/uniprot/Q86YB8) [Eukaryota] ERO1-like protein beta [Metazoa][Homo sapiens] (length=467 residues).

Site : 79- 84, Identity

yfryykvnlk_RPCPFW_aedghcsikd

--------------------------------------------------------------------------------

[NPSA](https://npsa-prabi.ibcp.fr/cgi-bin/seq_methonseq.pl?seqid=Q8IVF4&db=unipsp&process=5be3cc137c874450&seqtype=1) [gnl|unipsp|Q8IVF4](http://www.uniprot.org/uniprot/Q8IVF4) [Eukaryota] Dynein heavy chain 10, axonemal [Metazoa][Homo sapiens] (length=4471 residues).

Site : 995- 1000, Identity

nlmkylqkwk_RYRPLW_kldkaivmek

--------------------------------------------------------------------------------

[NPSA](https://npsa-prabi.ibcp.fr/cgi-bin/seq_methonseq.pl?seqid=Q8IVT2&db=unipsp&process=5be3cc137c874451&seqtype=1) [gnl|unipsp|Q8IVT2](http://www.uniprot.org/uniprot/Q8IVT2) [Eukaryota] Mitotic interactor and substrate of PLK1 [Metazoa][Homo sapiens] (length=679 residues).

Site : 262- 267, Identity

kgvvreenkv_RAVPTW_asvqvvddpg

--------------------------------------------------------------------------------

[NPSA](https://npsa-prabi.ibcp.fr/cgi-bin/seq_methonseq.pl?seqid=Q8IW19&db=unipsp&process=5be3cc137c874452&seqtype=1) [gnl|unipsp|Q8IW19](http://www.uniprot.org/uniprot/Q8IW19) [Eukaryota] Aprataxin and PNK-like factor [Metazoa][Homo sapiens] (length=511 residues).

Site : 184- 189, Identity

kqqpilaerk_RILPTW_mlaehlsdqn

--------------------------------------------------------------------------------

[NPSA](https://npsa-prabi.ibcp.fr/cgi-bin/seq_methonseq.pl?seqid=Q8IWB4&db=unipsp&process=5be3cc137c874453&seqtype=1) [gnl|unipsp|Q8IWB4](http://www.uniprot.org/uniprot/Q8IWB4) [Eukaryota] Spermatogenesis-associated protein 31A7 [Metazoa][Homo sapiens] (length=1347 residues).

Site : 900- 905, Identity

pawkqfqrap_RGIPSW_ndheplkppp

--------------------------------------------------------------------------------

[NPSA](https://npsa-prabi.ibcp.fr/cgi-bin/seq_methonseq.pl?seqid=Q8IWI9&db=unipsp&process=5be3cc137c874454&seqtype=1) [gnl|unipsp|Q8IWI9](http://www.uniprot.org/uniprot/Q8IWI9) [Eukaryota] MAX gene-associated protein [Metazoa][Homo sapiens] (length=3026 residues).

Site : 1155- 1160, Identity

icetepeqpv_RHYPLW_vkvegevdpe

--------------------------------------------------------------------------------

[NPSA](https://npsa-prabi.ibcp.fr/cgi-bin/seq_methonseq.pl?seqid=Q8IWX8&db=unipsp&process=5be3cc137c874455&seqtype=1) [gnl|unipsp|Q8IWX8](http://www.uniprot.org/uniprot/Q8IWX8) [Eukaryota] Calcium homeostasis endoplasmic reticulum protein [Metazoa][Homo sapiens] (length=916 residues).

Site : 467- 472, Identity

nshegmwgeq_RGDPGW_ngqrdapwnn

--------------------------------------------------------------------------------

[NPSA](https://npsa-prabi.ibcp.fr/cgi-bin/seq_methonseq.pl?seqid=Q8IXJ9&db=unipsp&process=5be3cc137c874456&seqtype=1) [gnl|unipsp|Q8IXJ9](http://www.uniprot.org/uniprot/Q8IXJ9) [Eukaryota] Putative Polycomb group protein ASXL1 [Metazoa][Homo sapiens] (length=1541 residues).

Site : 578- 583, Identity

kvppiriqls_RIKPPW_vvkgqptyqi

--------------------------------------------------------------------------------

[NPSA](https://npsa-prabi.ibcp.fr/cgi-bin/seq_methonseq.pl?seqid=Q8IXX5&db=unipsp&process=5be3cc137c874457&seqtype=1) [gnl|unipsp|Q8IXX5](http://www.uniprot.org/uniprot/Q8IXX5) [Eukaryota] Transmembrane protein 183A [Metazoa][Homo sapiens] (length=376 residues).

Site : 249- 254, Identity

lfwcrkivgn_RQEPMW_efnfkfkkqs

--------------------------------------------------------------------------------

[NPSA](https://npsa-prabi.ibcp.fr/cgi-bin/seq_methonseq.pl?seqid=Q8IYB8&db=unipsp&process=5be3cc137c874458&seqtype=1) [gnl|unipsp|Q8IYB8](http://www.uniprot.org/uniprot/Q8IYB8) [Eukaryota] ATP-dependent RNA helicase SUPV3L1, mitochondrial [Metazoa][Homo sapiens] (length=786 residues).

Site : 186- 191, Identity

kddlrkisdl_RIPPNW_ypdaramqrk

--------------------------------------------------------------------------------

[NPSA](https://npsa-prabi.ibcp.fr/cgi-bin/seq_methonseq.pl?seqid=Q8IYN6&db=unipsp&process=5be3cc137c874459&seqtype=1) [gnl|unipsp|Q8IYN6](http://www.uniprot.org/uniprot/Q8IYN6) [Eukaryota] UBA-like domain-containing protein 2 [Metazoa][Homo sapiens] (length=164 residues).

Site : 138- 143, Identity

sspttfhhlh_RPQPTW_ppgaqqggaq

--------------------------------------------------------------------------------

[NPSA](https://npsa-prabi.ibcp.fr/cgi-bin/seq_methonseq.pl?seqid=Q8IZ13&db=unipsp&process=5be3cc137c874460&seqtype=1) [gnl|unipsp|Q8IZ13](http://www.uniprot.org/uniprot/Q8IZ13) [Eukaryota] Protein ZBED8 [Metazoa][Homo sapiens] (length=594 residues).

Site : 410- 415, Identity

sareklsafv_RKFPFW_qkriekrnft

--------------------------------------------------------------------------------

[NPSA](https://npsa-prabi.ibcp.fr/cgi-bin/seq_methonseq.pl?seqid=Q8IZL2&db=unipsp&process=5be3cc137c874461&seqtype=1) [gnl|unipsp|Q8IZL2](http://www.uniprot.org/uniprot/Q8IZL2) [Eukaryota] Mastermind-like protein 2 [Metazoa][Homo sapiens] (length=1156 residues).

Site : 422- 427, Identity

aqpqtgsgas_RALPSW_qevshaqqlk

--------------------------------------------------------------------------------

[NPSA](https://npsa-prabi.ibcp.fr/cgi-bin/seq_methonseq.pl?seqid=Q8IZT6&db=unipsp&process=5be3cc137c874462&seqtype=1) [gnl|unipsp|Q8IZT6](http://www.uniprot.org/uniprot/Q8IZT6) [Eukaryota] Abnormal spindle-like microcephaly-associated protein [Metazoa][Homo sapiens] (length=3477 residues).

Site : 3444- 3449, Identity

etpvrtrivs_RLKPDW_vlrrdnmeei

--------------------------------------------------------------------------------

[NPSA](https://npsa-prabi.ibcp.fr/cgi-bin/seq_methonseq.pl?seqid=Q8N0W7&db=unipsp&process=5be3cc137c874463&seqtype=1) [gnl|unipsp|Q8N0W7](http://www.uniprot.org/uniprot/Q8N0W7) [Eukaryota] Fragile X mental retardation 1 neighbor protein [Metazoa][Homo sapiens] (length=255 residues).

Site : 52- 57, Identity

hpgyeaamad_RPQPGW_reslkmrvsk

--------------------------------------------------------------------------------

[NPSA](https://npsa-prabi.ibcp.fr/cgi-bin/seq_methonseq.pl?seqid=Q8N0Y7&db=unipsp&process=5be3cc137c874464&seqtype=1) [gnl|unipsp|Q8N0Y7](http://www.uniprot.org/uniprot/Q8N0Y7) [Eukaryota] Probable phosphoglycerate mutase 4 [Metazoa][Homo sapiens] (length=254 residues).

Site : 162- 167, Identity

syespkdtia_RALPFW_neeivpqike

--------------------------------------------------------------------------------

[NPSA](https://npsa-prabi.ibcp.fr/cgi-bin/seq_methonseq.pl?seqid=Q8N100&db=unipsp&process=5be3cc137c874465&seqtype=1) [gnl|unipsp|Q8N100](http://www.uniprot.org/uniprot/Q8N100) [Eukaryota] Protein atonal homolog 7 [Metazoa][Homo sapiens] (length=152 residues).

Site : 65- 70, Identity

glntafdrlr_RVVPQW_gqdkklskye

--------------------------------------------------------------------------------

[NPSA](https://npsa-prabi.ibcp.fr/cgi-bin/seq_methonseq.pl?seqid=Q8N118&db=unipsp&process=5be3cc137c874466&seqtype=1) [gnl|unipsp|Q8N118](http://www.uniprot.org/uniprot/Q8N118) [Eukaryota] Cytochrome P450 4X1 [Metazoa][Homo sapiens] (length=509 residues).

Site : 78- 83, Identity

kleeiiekyp_RAFPFW_igpfqaffci

--------------------------------------------------------------------------------

[NPSA](https://npsa-prabi.ibcp.fr/cgi-bin/seq_methonseq.pl?seqid=Q8N130&db=unipsp&process=5be3cc137c874467&seqtype=1) [gnl|unipsp|Q8N130](http://www.uniprot.org/uniprot/Q8N130) [Eukaryota] Sodium-dependent phosphate transport protein 2C [Metazoa][Homo sapiens] (length=599 residues).

Site : 536- 541, Identity

llvilvtvlq_RRRPAW_lpvrlrswaw

--------------------------------------------------------------------------------

[NPSA](https://npsa-prabi.ibcp.fr/cgi-bin/seq_methonseq.pl?seqid=Q8N1E2&db=unipsp&process=5be3cc137c874468&seqtype=1) [gnl|unipsp|Q8N1E2](http://www.uniprot.org/uniprot/Q8N1E2) [Eukaryota] Lysozyme g-like protein 1 [Metazoa][Homo sapiens] (length=194 residues).

Site : 143- 148, Identity

vlttrikeiq_RRFPTW_tpdqylrggl

--------------------------------------------------------------------------------

[NPSA](https://npsa-prabi.ibcp.fr/cgi-bin/seq_methonseq.pl?seqid=Q8N1M1&db=unipsp&process=5be3cc137c874469&seqtype=1) [gnl|unipsp|Q8N1M1](http://www.uniprot.org/uniprot/Q8N1M1) [Eukaryota] Bestrophin-3 [Metazoa][Homo sapiens] (length=668 residues).

Site : 445- 450, Identity

lldvpsrnpp_RASPTW_kkscfpegsp

--------------------------------------------------------------------------------

[NPSA](https://npsa-prabi.ibcp.fr/cgi-bin/seq_methonseq.pl?seqid=Q8N2Y8&db=unipsp&process=5be3cc137c874470&seqtype=1) [gnl|unipsp|Q8N2Y8](http://www.uniprot.org/uniprot/Q8N2Y8) [Eukaryota] Iporin [Metazoa][Homo sapiens] (length=1516 residues).

Site : 1339- 1344, Identity

paseealgre_RGWPFW_mgsppdsvla

--------------------------------------------------------------------------------

[NPSA](https://npsa-prabi.ibcp.fr/cgi-bin/seq_methonseq.pl?seqid=Q8N3F8&db=unipsp&process=5be3cc137c874471&seqtype=1) [gnl|unipsp|Q8N3F8](http://www.uniprot.org/uniprot/Q8N3F8) [Eukaryota] MICAL-like protein 1 [Metazoa][Homo sapiens] (length=863 residues).

Site : 366- 371, Identity

pkpsegtpap_RKDPPW_itlvqaepkk

--------------------------------------------------------------------------------

[NPSA](https://npsa-prabi.ibcp.fr/cgi-bin/seq_methonseq.pl?seqid=Q8N4C8&db=unipsp&process=5be3cc137c874472&seqtype=1) [gnl|unipsp|Q8N4C8](http://www.uniprot.org/uniprot/Q8N4C8) [Eukaryota] Misshapen-like kinase 1 [Metazoa][Homo sapiens] (length=1332 residues).

Site : 739- 744, Identity

pnassnpdlr_RSDPGW_ersdsvlpas

--------------------------------------------------------------------------------

[NPSA](https://npsa-prabi.ibcp.fr/cgi-bin/seq_methonseq.pl?seqid=Q8N4L4&db=unipsp&process=5be3cc137c874473&seqtype=1) [gnl|unipsp|Q8N4L4](http://www.uniprot.org/uniprot/Q8N4L4) [Eukaryota] Spermatid maturation protein 1 [Metazoa][Homo sapiens] (length=309 residues).

Site : 6- 11, Identity

‹mamve_RPRPEW_asyhncnsns

--------------------------------------------------------------------------------

[NPSA](https://npsa-prabi.ibcp.fr/cgi-bin/seq_methonseq.pl?seqid=Q8N5S9&db=unipsp&process=5be3cc137c874474&seqtype=1) [gnl|unipsp|Q8N5S9](http://www.uniprot.org/uniprot/Q8N5S9) [Eukaryota] Calcium/calmodulin-dependent protein kinase kinase 1 [Metazoa][Homo sapiens] (length=505 residues).

Site : 439- 444, Identity

vteeevknsv_RLIPSW_ttvilvksml

--------------------------------------------------------------------------------

[NPSA](https://npsa-prabi.ibcp.fr/cgi-bin/seq_methonseq.pl?seqid=Q8N7E2&db=unipsp&process=5be3cc137c874475&seqtype=1) [gnl|unipsp|Q8N7E2](http://www.uniprot.org/uniprot/Q8N7E2) [Eukaryota] E3 ubiquitin-protein ligase ZNF645 [Metazoa][Homo sapiens] (length=425 residues).

Site : 388- 393, Identity

qftqtdamdh_RRWPAW_krlspcpptr

--------------------------------------------------------------------------------

[NPSA](https://npsa-prabi.ibcp.fr/cgi-bin/seq_methonseq.pl?seqid=Q8N910&db=unipsp&process=5be3cc137c874476&seqtype=1) [gnl|unipsp|Q8N910](http://www.uniprot.org/uniprot/Q8N910) [Eukaryota] Putative uncharacterized protein C15orf56 [Metazoa][Homo sapiens] (length=161 residues).

Site : 153- 158, Identity

acgstaqwpp_RGDPTW_ris›

--------------------------------------------------------------------------------

[NPSA](https://npsa-prabi.ibcp.fr/cgi-bin/seq_methonseq.pl?seqid=Q8N9E0&db=unipsp&process=5be3cc137c874477&seqtype=1) [gnl|unipsp|Q8N9E0](http://www.uniprot.org/uniprot/Q8N9E0) [Eukaryota] Protein FAM133A [Metazoa][Homo sapiens] (length=248 residues).

Site : 36- 41, Identity

vgptiqdyln_RPRPTW_eevkkqlenk

--------------------------------------------------------------------------------

[NPSA](https://npsa-prabi.ibcp.fr/cgi-bin/seq_methonseq.pl?seqid=Q8N9H9&db=unipsp&process=5be3cc137c874478&seqtype=1) [gnl|unipsp|Q8N9H9](http://www.uniprot.org/uniprot/Q8N9H9) [Eukaryota] Uncharacterized protein C1orf127 [Metazoa][Homo sapiens] (length=656 residues).

Site : 26- 31, Identity

hcgpmfiqvs_RPLPLW_rdnrqtpwll

--------------------------------------------------------------------------------

[NPSA](https://npsa-prabi.ibcp.fr/cgi-bin/seq_methonseq.pl?seqid=Q8NA96&db=unipsp&process=5be3cc137c874479&seqtype=1) [gnl|unipsp|Q8NA96](http://www.uniprot.org/uniprot/Q8NA96) [Eukaryota] Putative uncharacterized protein FLJ35723 [Metazoa][Homo sapiens] (length=180 residues).

Site : 87- 92, Identity

hkpcgrslpr_RRNPGW_vswsdsmqad

--------------------------------------------------------------------------------

[NPSA](https://npsa-prabi.ibcp.fr/cgi-bin/seq_methonseq.pl?seqid=Q8NBS9&db=unipsp&process=5be3cc137c874480&seqtype=1) [gnl|unipsp|Q8NBS9](http://www.uniprot.org/uniprot/Q8NBS9) [Eukaryota] Thioredoxin domain-containing protein 5 [Metazoa][Homo sapiens] (length=432 residues).

Site : 94- 99, Identity

ffapwcghcq_RLQPTW_ndlgdkynsm

--------------------------------------------------------------------------------

[NPSA](https://npsa-prabi.ibcp.fr/cgi-bin/seq_methonseq.pl?seqid=Q8NCN5&db=unipsp&process=5be3cc137c874481&seqtype=1) [gnl|unipsp|Q8NCN5](http://www.uniprot.org/uniprot/Q8NCN5) [Eukaryota] Pyruvate dehydrogenase phosphatase regulatory subunit, mitochondrial [Metazoa][Homo sapiens] (length=879 residues).

Site : 14- 19, Identity

yrllsivgrq_RASPGW_qnwssarnst

--------------------------------------------------------------------------------

[NPSA](https://npsa-prabi.ibcp.fr/cgi-bin/seq_methonseq.pl?seqid=Q8NCW5&db=unipsp&process=5be3cc137c874482&seqtype=1) [gnl|unipsp|Q8NCW5](http://www.uniprot.org/uniprot/Q8NCW5) [Eukaryota] NAD(P)H-hydrate epimerase [Metazoa][Homo sapiens] (length=288 residues).

Site : 30- 35, Identity

priksqtiac_RSGPTW_wgpqrlnsgg

--------------------------------------------------------------------------------

[NPSA](https://npsa-prabi.ibcp.fr/cgi-bin/seq_methonseq.pl?seqid=Q8NDH2&db=unipsp&process=5be3cc137c874483&seqtype=1) [gnl|unipsp|Q8NDH2](http://www.uniprot.org/uniprot/Q8NDH2) [Eukaryota] Coiled-coil domain-containing protein 168 [Metazoa][Homo sapiens] (length=2452 residues).

Site : 170- 175, Identity

sitehsplsk_RKEPQW_gmkeragqkq

--------------------------------------------------------------------------------

[NPSA](https://npsa-prabi.ibcp.fr/cgi-bin/seq_methonseq.pl?seqid=Q8NDX1&db=unipsp&process=5be3cc137c874484&seqtype=1) [gnl|unipsp|Q8NDX1](http://www.uniprot.org/uniprot/Q8NDX1) [Eukaryota] PH and SEC7 domain-containing protein 4 [Metazoa][Homo sapiens] (length=1056 residues).

Site : 401- 406, Identity

waslspegwq_RGGPFW_pqvtlnsqdr

--------------------------------------------------------------------------------

[NPSA](https://npsa-prabi.ibcp.fr/cgi-bin/seq_methonseq.pl?seqid=Q8NEF3&db=unipsp&process=5be3cc137c874485&seqtype=1) [gnl|unipsp|Q8NEF3](http://www.uniprot.org/uniprot/Q8NEF3) [Eukaryota] Coiled-coil domain-containing protein 112 [Metazoa][Homo sapiens] (length=446 residues).

Site : 433- 438, Identity

gsgpllhiph_RAIPTW_rqgiqrrv›

--------------------------------------------------------------------------------

[NPSA](https://npsa-prabi.ibcp.fr/cgi-bin/seq_methonseq.pl?seqid=Q8NF37&db=unipsp&process=5be3cc137c874486&seqtype=1) [gnl|unipsp|Q8NF37](http://www.uniprot.org/uniprot/Q8NF37) [Eukaryota] Lysophosphatidylcholine acyltransferase 1 [Metazoa][Homo sapiens] (length=534 residues).

Site : 158- 163, Identity

mssivmkaes_RDIPIW_gtliqyirpv

--------------------------------------------------------------------------------

[NPSA](https://npsa-prabi.ibcp.fr/cgi-bin/seq_methonseq.pl?seqid=Q8NF91&db=unipsp&process=5be3cc137c874487&seqtype=1) [gnl|unipsp|Q8NF91](http://www.uniprot.org/uniprot/Q8NF91) [Eukaryota] Nesprin-1 [Metazoa][Homo sapiens] (length=8797 residues).

Site : 648- 653, Identity

qsenakkdff_RNLPHW_iqqhtamnda

--------------------------------------------------------------------------------

[NPSA](https://npsa-prabi.ibcp.fr/cgi-bin/seq_methonseq.pl?seqid=Q8NFR9&db=unipsp&process=5be3cc137c874488&seqtype=1) [gnl|unipsp|Q8NFR9](http://www.uniprot.org/uniprot/Q8NFR9) [Eukaryota] Interleukin-17 receptor E [Metazoa][Homo sapiens] (length=667 residues).

Site : 29- 34, Identity

dlsdsagigf_RHLPHW_ntrcplasht

--------------------------------------------------------------------------------

[NPSA](https://npsa-prabi.ibcp.fr/cgi-bin/seq_methonseq.pl?seqid=Q8NFT2&db=unipsp&process=5be3cc137c874489&seqtype=1) [gnl|unipsp|Q8NFT2](http://www.uniprot.org/uniprot/Q8NFT2) [Eukaryota] Metalloreductase STEAP2 [Metazoa][Homo sapiens] (length=490 residues).

Site : 289- 294, Identity

ayqlyygtky_RRFPPW_letwlqcrkq

--------------------------------------------------------------------------------

[NPSA](https://npsa-prabi.ibcp.fr/cgi-bin/seq_methonseq.pl?seqid=Q8NHV5&db=unipsp&process=5be3cc137c874490&seqtype=1) [gnl|unipsp|Q8NHV5](http://www.uniprot.org/uniprot/Q8NHV5) [Eukaryota] Uncharacterized protein C16orf52 [Metazoa][Homo sapiens] (length=167 residues).

Site : 60- 65, Identity

hgrdrtcipp_RLPPEW_vttlffiimg

--------------------------------------------------------------------------------

[NPSA](https://npsa-prabi.ibcp.fr/cgi-bin/seq_methonseq.pl?seqid=Q8NI28&db=unipsp&process=5be3cc137c874491&seqtype=1) [gnl|unipsp|Q8NI28](http://www.uniprot.org/uniprot/Q8NI28) [Eukaryota] Putative uncharacterized protein encoded by LINC01006 [Metazoa][Homo sapiens] (length=216 residues).

Site : 202- 207, Identity

glavcvwmqa_RVSPAW_paglfllpr›

--------------------------------------------------------------------------------

[NPSA](https://npsa-prabi.ibcp.fr/cgi-bin/seq_methonseq.pl?seqid=Q8TAE8&db=unipsp&process=5be3cc137c874492&seqtype=1) [gnl|unipsp|Q8TAE8](http://www.uniprot.org/uniprot/Q8TAE8) [Eukaryota] Growth arrest and DNA damage-inducible proteins-interacting protein 1 [Metazoa][Homo sapiens] (length=222 residues).

Site : 34- 39, Identity

gyrarppprr_RPGPRW_pdpedlltpr

--------------------------------------------------------------------------------

[NPSA](https://npsa-prabi.ibcp.fr/cgi-bin/seq_methonseq.pl?seqid=Q8TB40&db=unipsp&process=5be3cc137c874493&seqtype=1) [gnl|unipsp|Q8TB40](http://www.uniprot.org/uniprot/Q8TB40) [Eukaryota] Abhydrolase domain-containing protein 4 [Metazoa][Homo sapiens] (length=342 residues).

Site : 185- 190, Identity

plrptnpsei_RAPPAW_vkavasvlgr

--------------------------------------------------------------------------------

[NPSA](https://npsa-prabi.ibcp.fr/cgi-bin/seq_methonseq.pl?seqid=Q8TB68&db=unipsp&process=5be3cc137c874494&seqtype=1) [gnl|unipsp|Q8TB68](http://www.uniprot.org/uniprot/Q8TB68) [Eukaryota] Proline-rich protein 7 [Metazoa][Homo sapiens] (length=274 residues).

Site : 238- 243, Identity

alclqadrgr_RVFPSW_tdselssrep

--------------------------------------------------------------------------------

[NPSA](https://npsa-prabi.ibcp.fr/cgi-bin/seq_methonseq.pl?seqid=Q8TBR7&db=unipsp&process=5be3cc137c874495&seqtype=1) [gnl|unipsp|Q8TBR7](http://www.uniprot.org/uniprot/Q8TBR7) [Eukaryota] Protein FAM57A [Metazoa][Homo sapiens] (length=257 residues).

Site : 25- 30, Identity

lfalctwalr_RSQPGW_srtdcvmist

--------------------------------------------------------------------------------

[NPSA](https://npsa-prabi.ibcp.fr/cgi-bin/seq_methonseq.pl?seqid=Q8TC57&db=unipsp&process=5be3cc137c874496&seqtype=1) [gnl|unipsp|Q8TC57](http://www.uniprot.org/uniprot/Q8TC57) [Eukaryota] Meiosis 1 arrest protein [Metazoa][Homo sapiens] (length=530 residues).

Site : 462- 467, Identity

lssiyakpqg_RLHPHW_esraprkhpc

--------------------------------------------------------------------------------

[NPSA](https://npsa-prabi.ibcp.fr/cgi-bin/seq_methonseq.pl?seqid=Q8TCC3&db=unipsp&process=5be3cc137c874497&seqtype=1) [gnl|unipsp|Q8TCC3](http://www.uniprot.org/uniprot/Q8TCC3) [Eukaryota] 39S ribosomal protein L30, mitochondrial [Metazoa][Homo sapiens] (length=161 residues).

Site : 76- 81, Identity

lhivtrikst_RRRPYW_ekdiikmlgl

--------------------------------------------------------------------------------

[NPSA](https://npsa-prabi.ibcp.fr/cgi-bin/seq_methonseq.pl?seqid=Q8TD31&db=unipsp&process=5be3cc137c874498&seqtype=1) [gnl|unipsp|Q8TD31](http://www.uniprot.org/uniprot/Q8TD31) [Eukaryota] Coiled-coil alpha-helical rod protein 1 [Metazoa][Homo sapiens] (length=782 residues).

Site : 26- 31, Identity

fqarplstlp_RMAPTW_lsdiplvqpp

--------------------------------------------------------------------------------

[NPSA](https://npsa-prabi.ibcp.fr/cgi-bin/seq_methonseq.pl?seqid=Q8TD43&db=unipsp&process=5be3cc137c874499&seqtype=1) [gnl|unipsp|Q8TD43](http://www.uniprot.org/uniprot/Q8TD43) [Eukaryota] Transient receptor potential cation channel subfamily M member 4 [Metazoa][Homo sapiens] (length=1214 residues).

Site : 644- 649, Identity

sevraarlll_RRCPLW_gdatclqlam

--------------------------------------------------------------------------------

[NPSA](https://npsa-prabi.ibcp.fr/cgi-bin/seq_methonseq.pl?seqid=Q8TDJ6&db=unipsp&process=5be3cc137c874500&seqtype=1) [gnl|unipsp|Q8TDJ6](http://www.uniprot.org/uniprot/Q8TDJ6) [Eukaryota] DmX-like protein 2 [Metazoa][Homo sapiens] (length=3036 residues).

Site : 1622- 1627, Identity

elinmipaiq_RGDPQW_selramgigw

--------------------------------------------------------------------------------

[NPSA](https://npsa-prabi.ibcp.fr/cgi-bin/seq_methonseq.pl?seqid=Q8TDW0&db=unipsp&process=5be3cc137c874501&seqtype=1) [gnl|unipsp|Q8TDW0](http://www.uniprot.org/uniprot/Q8TDW0) [Eukaryota] Volume-regulated anion channel subunit LRRC8C [Metazoa][Homo sapiens] (length=803 residues).

Site : 504- 509, Identity

kvlsvkfddm_RELPPW_myglrnleel

--------------------------------------------------------------------------------

[NPSA](https://npsa-prabi.ibcp.fr/cgi-bin/seq_methonseq.pl?seqid=Q8TF01&db=unipsp&process=5be3cc137c874502&seqtype=1) [gnl|unipsp|Q8TF01](http://www.uniprot.org/uniprot/Q8TF01) [Eukaryota] Arginine/serine-rich protein PNISR [Metazoa][Homo sapiens] (length=805 residues).

Site : 230- 235, Identity

eppqidavkr_RTLPAW_ireglekmer

--------------------------------------------------------------------------------

[NPSA](https://npsa-prabi.ibcp.fr/cgi-bin/seq_methonseq.pl?seqid=Q8WTS1&db=unipsp&process=5be3cc137c874503&seqtype=1) [gnl|unipsp|Q8WTS1](http://www.uniprot.org/uniprot/Q8WTS1) [Eukaryota] 1-acylglycerol-3-phosphate O-acyltransferase ABHD5 [Metazoa][Homo sapiens] (length=349 residues).

Site : 192- 197, Identity

perpdladqd_RPIPVW_iralgaaltp

--------------------------------------------------------------------------------

[NPSA](https://npsa-prabi.ibcp.fr/cgi-bin/seq_methonseq.pl?seqid=Q8WVQ1&db=unipsp&process=5be3cc137c874504&seqtype=1) [gnl|unipsp|Q8WVQ1](http://www.uniprot.org/uniprot/Q8WVQ1) [Eukaryota] Soluble calcium-activated nucleotidase 1 [Metazoa][Homo sapiens] (length=401 residues).

Site : 38- 43, Identity

lasmtkaadp_RFRPRW_kviltffvga

--------------------------------------------------------------------------------

[NPSA](https://npsa-prabi.ibcp.fr/cgi-bin/seq_methonseq.pl?seqid=Q8WW32&db=unipsp&process=5be3cc137c874505&seqtype=1) [gnl|unipsp|Q8WW32](http://www.uniprot.org/uniprot/Q8WW32) [Eukaryota] High mobility group protein B4 [Metazoa][Homo sapiens] (length=186 residues).

Site : 113- 118, Identity

fcqdhyaqlk_RENPNW_svvqvakatg

--------------------------------------------------------------------------------

[NPSA](https://npsa-prabi.ibcp.fr/cgi-bin/seq_methonseq.pl?seqid=Q8WWN8&db=unipsp&process=5be3cc137c874506&seqtype=1) [gnl|unipsp|Q8WWN8](http://www.uniprot.org/uniprot/Q8WWN8) [Eukaryota] Arf-GAP with Rho-GAP domain, ANK repeat and PH domain-containing protein 3 [Metazoa][Homo sapiens] (length=1544 residues).

Site : 995- 1000, Identity

relddpvtsa_RLLPRW_reaaelpqkn

--------------------------------------------------------------------------------

[NPSA](https://npsa-prabi.ibcp.fr/cgi-bin/seq_methonseq.pl?seqid=Q8WWV6&db=unipsp&process=5be3cc137c874507&seqtype=1) [gnl|unipsp|Q8WWV6](http://www.uniprot.org/uniprot/Q8WWV6) [Eukaryota] High affinity immunoglobulin alpha and immunoglobulin mu Fc receptor [Metazoa][Homo sapiens] (length=532 residues).

Site : 21- 26, Identity

qgssfalpqk_RPHPRW_lwegslpsrt

--------------------------------------------------------------------------------

[NPSA](https://npsa-prabi.ibcp.fr/cgi-bin/seq_methonseq.pl?seqid=Q8WXB4&db=unipsp&process=5be3cc137c874508&seqtype=1) [gnl|unipsp|Q8WXB4](http://www.uniprot.org/uniprot/Q8WXB4) [Eukaryota] Zinc finger protein 606 [Metazoa][Homo sapiens] (length=792 residues).

Site : 130- 135, Identity

pwsveqacpq_RTCPEW_vrnleskali

--------------------------------------------------------------------------------

[NPSA](https://npsa-prabi.ibcp.fr/cgi-bin/seq_methonseq.pl?seqid=Q8WXF3&db=unipsp&process=5be3cc137c874509&seqtype=1) [gnl|unipsp|Q8WXF3](http://www.uniprot.org/uniprot/Q8WXF3) [Eukaryota] Relaxin-3 [Metazoa][Homo sapiens] (length=142 residues).

Site : 102- 107, Identity

altkspqafy_RGRPSW_qgtpgvlrgs

--------------------------------------------------------------------------------

[NPSA](https://npsa-prabi.ibcp.fr/cgi-bin/seq_methonseq.pl?seqid=Q8WXH5&db=unipsp&process=5be3cc137c874510&seqtype=1) [gnl|unipsp|Q8WXH5](http://www.uniprot.org/uniprot/Q8WXH5) [Eukaryota] Suppressor of cytokine signaling 4 [Metazoa][Homo sapiens] (length=440 residues).

Site : 244- 249, Identity

iltlctssrk_RNKPKW_dlddeilqle

--------------------------------------------------------------------------------

[NPSA](https://npsa-prabi.ibcp.fr/cgi-bin/seq_methonseq.pl?seqid=Q8WXT5&db=unipsp&process=5be3cc137c874511&seqtype=1) [gnl|unipsp|Q8WXT5](http://www.uniprot.org/uniprot/Q8WXT5) [Eukaryota] Forkhead box protein D4-like 4 [Metazoa][Homo sapiens] (length=416 residues).

Site : 147- 152, Identity

fisgrfpyyr_RKFPAW_qnsirhnlsl

--------------------------------------------------------------------------------

[NPSA](https://npsa-prabi.ibcp.fr/cgi-bin/seq_methonseq.pl?seqid=Q8WZ75&db=unipsp&process=5be3cc137c874512&seqtype=1) [gnl|unipsp|Q8WZ75](http://www.uniprot.org/uniprot/Q8WZ75) [Eukaryota] Roundabout homolog 4 [Metazoa][Homo sapiens] (length=1007 residues).

Site : 974- 979, Identity

mevshtqrlg_RGMPPW_ppdsqissqr

--------------------------------------------------------------------------------

[NPSA](https://npsa-prabi.ibcp.fr/cgi-bin/seq_methonseq.pl?seqid=Q92574&db=unipsp&process=5be3cc137c874513&seqtype=1) [gnl|unipsp|Q92574](http://www.uniprot.org/uniprot/Q92574) [Eukaryota] Hamartin [Metazoa][Homo sapiens] (length=1164 residues).

Site : 98- 103, Identity

silsllghvi_RLQPSW_khklsqapll

--------------------------------------------------------------------------------

[NPSA](https://npsa-prabi.ibcp.fr/cgi-bin/seq_methonseq.pl?seqid=Q92781&db=unipsp&process=5be3cc137c874514&seqtype=1) [gnl|unipsp|Q92781](http://www.uniprot.org/uniprot/Q92781) [Eukaryota] 11-cis retinol dehydrogenase [Metazoa][Homo sapiens] (length=318 residues).

Site : 280- 285, Identity

haltarhprt_RYSPGW_dakllwlpas

--------------------------------------------------------------------------------

[NPSA](https://npsa-prabi.ibcp.fr/cgi-bin/seq_methonseq.pl?seqid=Q92887&db=unipsp&process=5be3cc137c874515&seqtype=1) [gnl|unipsp|Q92887](http://www.uniprot.org/uniprot/Q92887) [Eukaryota] Canalicular multispecific organic anion transporter 1 [Metazoa][Homo sapiens] (length=1545 residues).

Site : 1289- 1294, Identity

eneapwvtdk_RPPPDW_pskgkiqfnn

--------------------------------------------------------------------------------

[NPSA](https://npsa-prabi.ibcp.fr/cgi-bin/seq_methonseq.pl?seqid=Q969E1&db=unipsp&process=5be3cc137c874516&seqtype=1) [gnl|unipsp|Q969E1](http://www.uniprot.org/uniprot/Q969E1) [Eukaryota] Liver-expressed antimicrobial peptide 2 [Metazoa][Homo sapiens] (length=77 residues).

Site : 37- 42, Identity

evssakrrpr_RMTPFW_rgvslrpiga

--------------------------------------------------------------------------------

[NPSA](https://npsa-prabi.ibcp.fr/cgi-bin/seq_methonseq.pl?seqid=Q969J5&db=unipsp&process=5be3cc137c874517&seqtype=1) [gnl|unipsp|Q969J5](http://www.uniprot.org/uniprot/Q969J5) [Eukaryota] Interleukin-22 receptor subunit alpha-2 [Metazoa][Homo sapiens] (length=263 residues).

Site : 151- 156, Identity

gsysewsmtp_RFTPWW_etkidppvmn

--------------------------------------------------------------------------------

[NPSA](https://npsa-prabi.ibcp.fr/cgi-bin/seq_methonseq.pl?seqid=Q969Z3&db=unipsp&process=5be3cc137c874518&seqtype=1) [gnl|unipsp|Q969Z3](http://www.uniprot.org/uniprot/Q969Z3) [Eukaryota] Mitochondrial amidoxime reducing component 2 [Metazoa][Homo sapiens] (length=335 residues).

Site : 17- 22, Identity

salarlglpa_RPWPRW_lgvaalglaa

--------------------------------------------------------------------------------

[NPSA](https://npsa-prabi.ibcp.fr/cgi-bin/seq_methonseq.pl?seqid=Q96A35&db=unipsp&process=5be3cc137c874519&seqtype=1) [gnl|unipsp|Q96A35](http://www.uniprot.org/uniprot/Q96A35) [Eukaryota] 39S ribosomal protein L24, mitochondrial [Metazoa][Homo sapiens] (length=216 residues).

Site : 33- 38, Identity

msppgsvadk_RKNPPW_irrrpvvvep

--------------------------------------------------------------------------------

[NPSA](https://npsa-prabi.ibcp.fr/cgi-bin/seq_methonseq.pl?seqid=Q96BV0&db=unipsp&process=5be3cc137c874520&seqtype=1) [gnl|unipsp|Q96BV0](http://www.uniprot.org/uniprot/Q96BV0) [Eukaryota] Zinc finger protein 775 [Metazoa][Homo sapiens] (length=537 residues).

Site : 245- 250, Identity

racrlqpgpp_RGRPEW_awlglcqgww

--------------------------------------------------------------------------------

[NPSA](https://npsa-prabi.ibcp.fr/cgi-bin/seq_methonseq.pl?seqid=Q96C45&db=unipsp&process=5be3cc137c874521&seqtype=1) [gnl|unipsp|Q96C45](http://www.uniprot.org/uniprot/Q96C45) [Eukaryota] Serine/threonine-protein kinase ULK4 [Metazoa][Homo sapiens] (length=1275 residues).

Site : 519- 524, Identity

plfqlliqhl_RIAPNW_dirakvahvi

--------------------------------------------------------------------------------

[NPSA](https://npsa-prabi.ibcp.fr/cgi-bin/seq_methonseq.pl?seqid=Q96CC6&db=unipsp&process=5be3cc137c874522&seqtype=1) [gnl|unipsp|Q96CC6](http://www.uniprot.org/uniprot/Q96CC6) [Eukaryota] Inactive rhomboid protein 1 [Metazoa][Homo sapiens] (length=855 residues).

Site : 14- 19, Identity

arrdstsslq_RKKPPW_lkldipsavp

--------------------------------------------------------------------------------

[NPSA](https://npsa-prabi.ibcp.fr/cgi-bin/seq_methonseq.pl?seqid=Q96DP5&db=unipsp&process=5be3cc137c874523&seqtype=1) [gnl|unipsp|Q96DP5](http://www.uniprot.org/uniprot/Q96DP5) [Eukaryota] Methionyl-tRNA formyltransferase, mitochondrial [Metazoa][Homo sapiens] (length=389 residues).

Site : 21- 26, Identity

pplahgarrg_RPSPQW_ralarlgwed

Site : 43- 48, Identity

gwedcrdsrv_REKPPW_rvlffgtdqf

--------------------------------------------------------------------------------

[NPSA](https://npsa-prabi.ibcp.fr/cgi-bin/seq_methonseq.pl?seqid=Q96EH5&db=unipsp&process=5be3cc137c874524&seqtype=1) [gnl|unipsp|Q96EH5](http://www.uniprot.org/uniprot/Q96EH5) [Eukaryota] 60S ribosomal protein L39-like [Metazoa][Homo sapiens] (length=51 residues).

Site : 21- 26, Identity

rflakkqkqn_RPIPQW_iqmkpgskir

--------------------------------------------------------------------------------

[NPSA](https://npsa-prabi.ibcp.fr/cgi-bin/seq_methonseq.pl?seqid=Q96EN9&db=unipsp&process=5be3cc137c874525&seqtype=1) [gnl|unipsp|Q96EN9](http://www.uniprot.org/uniprot/Q96EN9) [Eukaryota] Uncharacterized protein C19orf60 [Metazoa][Homo sapiens] (length=201 residues).

Site : 26- 31, Identity

aaeeatearg_REEPAW_pwkdapirtl

--------------------------------------------------------------------------------

[NPSA](https://npsa-prabi.ibcp.fr/cgi-bin/seq_methonseq.pl?seqid=Q96GJ1&db=unipsp&process=5be3cc137c874526&seqtype=1) [gnl|unipsp|Q96GJ1](http://www.uniprot.org/uniprot/Q96GJ1) [Eukaryota] tRNA (uracil(54)-C(5))-methyltransferase homolog [Metazoa][Homo sapiens] (length=504 residues).

Site : 34- 39, Identity

skpgllpwya_RNPPGW_sqlflgtvck

--------------------------------------------------------------------------------

[NPSA](https://npsa-prabi.ibcp.fr/cgi-bin/seq_methonseq.pl?seqid=Q96GP6&db=unipsp&process=5be3cc137c874527&seqtype=1) [gnl|unipsp|Q96GP6](http://www.uniprot.org/uniprot/Q96GP6) [Eukaryota] Scavenger receptor class F member 2 [Metazoa][Homo sapiens] (length=870 residues).

Site : 171- 176, Identity

gtchprsgac_RCEPGW_wgaqcasacy

--------------------------------------------------------------------------------

[NPSA](https://npsa-prabi.ibcp.fr/cgi-bin/seq_methonseq.pl?seqid=Q96GQ5&db=unipsp&process=5be3cc137c874528&seqtype=1) [gnl|unipsp|Q96GQ5](http://www.uniprot.org/uniprot/Q96GQ5) [Eukaryota] RUS1 family protein C16orf58 [Metazoa][Homo sapiens] (length=468 residues).

Site : 313- 318, Identity

gevldptaan_RMEPLW_tgfwpapsls

--------------------------------------------------------------------------------

[NPSA](https://npsa-prabi.ibcp.fr/cgi-bin/seq_methonseq.pl?seqid=Q96GX1&db=unipsp&process=5be3cc137c874529&seqtype=1) [gnl|unipsp|Q96GX1](http://www.uniprot.org/uniprot/Q96GX1) [Eukaryota] Tectonic-2 [Metazoa][Homo sapiens] (length=697 residues).

Site : 226- 231, Identity

dqlcsagttt_RGVPDW_fpflcvqspl

--------------------------------------------------------------------------------

[NPSA](https://npsa-prabi.ibcp.fr/cgi-bin/seq_methonseq.pl?seqid=Q96HE7&db=unipsp&process=5be3cc137c874530&seqtype=1) [gnl|unipsp|Q96HE7](http://www.uniprot.org/uniprot/Q96HE7) [Eukaryota] ERO1-like protein alpha [Metazoa][Homo sapiens] (length=468 residues).

Site : 83- 88, Identity

yfryykvnlk_RPCPFW_ndisqcgrrd

--------------------------------------------------------------------------------

[NPSA](https://npsa-prabi.ibcp.fr/cgi-bin/seq_methonseq.pl?seqid=Q96ID5&db=unipsp&process=5be3cc137c874531&seqtype=1) [gnl|unipsp|Q96ID5](http://www.uniprot.org/uniprot/Q96ID5) [Eukaryota] Immunoglobulin superfamily member 21 [Metazoa][Homo sapiens] (length=467 residues).

Site : 272- 277, Identity

tenipetvvs_REFPRW_vhsaeptyfl

--------------------------------------------------------------------------------

[NPSA](https://npsa-prabi.ibcp.fr/cgi-bin/seq_methonseq.pl?seqid=Q96KG7&db=unipsp&process=5be3cc137c874532&seqtype=1) [gnl|unipsp|Q96KG7](http://www.uniprot.org/uniprot/Q96KG7) [Eukaryota] Multiple epidermal growth factor-like domains protein 10 [Metazoa][Homo sapiens] (length=1140 residues).

Site : 558- 563, Identity

dgchpttghc_RCLPGW_sgvhcdsvca

--------------------------------------------------------------------------------

[NPSA](https://npsa-prabi.ibcp.fr/cgi-bin/seq_methonseq.pl?seqid=Q96L96&db=unipsp&process=5be3cc137c874533&seqtype=1) [gnl|unipsp|Q96L96](http://www.uniprot.org/uniprot/Q96L96) [Eukaryota] Alpha-protein kinase 3 [Metazoa][Homo sapiens] (length=1907 residues).

Site : 40- 45, Identity

glvlwlpglp_RSSPSW_pavdlaplap

--------------------------------------------------------------------------------

[NPSA](https://npsa-prabi.ibcp.fr/cgi-bin/seq_methonseq.pl?seqid=Q96M19&db=unipsp&process=5be3cc137c874534&seqtype=1) [gnl|unipsp|Q96M19](http://www.uniprot.org/uniprot/Q96M19) [Eukaryota] Putative transmembrane protein encoded by LINC00477 [Metazoa][Homo sapiens] (length=166 residues).

Site : 97- 102, Identity

mdleglewlg_RASPSW_wiffssspsh

--------------------------------------------------------------------------------

[NPSA](https://npsa-prabi.ibcp.fr/cgi-bin/seq_methonseq.pl?seqid=Q96M86&db=unipsp&process=5be3cc137c874535&seqtype=1) [gnl|unipsp|Q96M86](http://www.uniprot.org/uniprot/Q96M86) [Eukaryota] Dynein heavy chain domain-containing protein 1 [Metazoa][Homo sapiens] (length=4753 residues).

Site : 4419- 4424, Identity

qsrallsalq_RSSPVW_vpesrrgaql

--------------------------------------------------------------------------------

[NPSA](https://npsa-prabi.ibcp.fr/cgi-bin/seq_methonseq.pl?seqid=Q96N87&db=unipsp&process=5be3cc137c874536&seqtype=1) [gnl|unipsp|Q96N87](http://www.uniprot.org/uniprot/Q96N87) [Eukaryota] Sodium-dependent neutral amino acid transporter B(0)AT3 [Metazoa][Homo sapiens] (length=628 residues).

Site : 511- 516, Identity

cddiawmtgr_RPSPYW_rltwrvvspl

--------------------------------------------------------------------------------

[NPSA](https://npsa-prabi.ibcp.fr/cgi-bin/seq_methonseq.pl?seqid=Q96P20&db=unipsp&process=5be3cc137c874537&seqtype=1) [gnl|unipsp|Q96P20](http://www.uniprot.org/uniprot/Q96P20) [Eukaryota] NACHT, LRR and PYD domains-containing protein 3 [Metazoa][Homo sapiens] (length=1036 residues).

Site : 89- 94, Identity

nrrdlyekak_RDEPKW_gsdnarvsnp

--------------------------------------------------------------------------------

[NPSA](https://npsa-prabi.ibcp.fr/cgi-bin/seq_methonseq.pl?seqid=Q96PG2&db=unipsp&process=5be3cc137c874538&seqtype=1) [gnl|unipsp|Q96PG2](http://www.uniprot.org/uniprot/Q96PG2) [Eukaryota] Membrane-spanning 4-domains subfamily A member 10 [Metazoa][Homo sapiens] (length=267 residues).

Site : 14- 19, Identity

eatvipsrca_RGLPSW_qvlspvqpwq

--------------------------------------------------------------------------------

[NPSA](https://npsa-prabi.ibcp.fr/cgi-bin/seq_methonseq.pl?seqid=Q96PU8&db=unipsp&process=5be3cc137c874539&seqtype=1) [gnl|unipsp|Q96PU8](http://www.uniprot.org/uniprot/Q96PU8) [Eukaryota] Protein quaking [Metazoa][Homo sapiens] (length=341 residues).

Site : 139- 144, Identity

mrdkkkeeqn_RGKPNW_ehlnedlhvl

--------------------------------------------------------------------------------

[NPSA](https://npsa-prabi.ibcp.fr/cgi-bin/seq_methonseq.pl?seqid=Q96PV7&db=unipsp&process=5be3cc137c874540&seqtype=1) [gnl|unipsp|Q96PV7](http://www.uniprot.org/uniprot/Q96PV7) [Eukaryota] Protein FAM193B [Metazoa][Homo sapiens] (length=902 residues).

Site : 777- 782, Identity

aeagegsrgs_RPGPGW_agspktekek

--------------------------------------------------------------------------------

[NPSA](https://npsa-prabi.ibcp.fr/cgi-bin/seq_methonseq.pl?seqid=Q96RR1&db=unipsp&process=5be3cc137c874541&seqtype=1) [gnl|unipsp|Q96RR1](http://www.uniprot.org/uniprot/Q96RR1) [Eukaryota] Twinkle protein, mitochondrial [Metazoa][Homo sapiens] (length=684 residues).

Site : 160- 165, Identity

dseevrriwn_RAIPLW_elpdqeevql

--------------------------------------------------------------------------------

[NPSA](https://npsa-prabi.ibcp.fr/cgi-bin/seq_methonseq.pl?seqid=Q96RT8&db=unipsp&process=5be3cc137c874542&seqtype=1) [gnl|unipsp|Q96RT8](http://www.uniprot.org/uniprot/Q96RT8) [Eukaryota] Gamma-tubulin complex component 5 [Metazoa][Homo sapiens] (length=1024 residues).

Site : 3- 8, Identity

‹ma_RHGPPW_srldaqqerd

--------------------------------------------------------------------------------

[NPSA](https://npsa-prabi.ibcp.fr/cgi-bin/seq_methonseq.pl?seqid=Q96S06&db=unipsp&process=5be3cc137c874543&seqtype=1) [gnl|unipsp|Q96S06](http://www.uniprot.org/uniprot/Q96S06) [Eukaryota] Lipase maturation factor 1 [Metazoa][Homo sapiens] (length=567 residues).

Site : 506- 511, Identity

sllahnpfag_RPPPRW_vrgehyrykf

--------------------------------------------------------------------------------

[NPSA](https://npsa-prabi.ibcp.fr/cgi-bin/seq_methonseq.pl?seqid=Q96S79&db=unipsp&process=5be3cc137c874544&seqtype=1) [gnl|unipsp|Q96S79](http://www.uniprot.org/uniprot/Q96S79) [Eukaryota] Ras-like protein family member 10B [Metazoa][Homo sapiens] (length=203 residues).

Site : 136- 141, Identity

vgnkrdlqrg_RVIPRW_nvshlvrktw

--------------------------------------------------------------------------------

[NPSA](https://npsa-prabi.ibcp.fr/cgi-bin/seq_methonseq.pl?seqid=Q96ST8&db=unipsp&process=5be3cc137c874545&seqtype=1) [gnl|unipsp|Q96ST8](http://www.uniprot.org/uniprot/Q96ST8) [Eukaryota] Centrosomal protein of 89 kDa [Metazoa][Homo sapiens] (length=783 residues).

Site : 100- 105, Identity

iepyattsql_RPRPNW_qsemgrrssl

--------------------------------------------------------------------------------

[NPSA](https://npsa-prabi.ibcp.fr/cgi-bin/seq_methonseq.pl?seqid=Q96T37&db=unipsp&process=5be3cc137c874546&seqtype=1) [gnl|unipsp|Q96T37](http://www.uniprot.org/uniprot/Q96T37) [Eukaryota] Putative RNA-binding protein 15 [Metazoa][Homo sapiens] (length=977 residues).

Site : 11- 16, Identity

mrtagrdpvp_RRSPRW_rravplcets

--------------------------------------------------------------------------------

[NPSA](https://npsa-prabi.ibcp.fr/cgi-bin/seq_methonseq.pl?seqid=Q96T58&db=unipsp&process=5be3cc137c874547&seqtype=1) [gnl|unipsp|Q96T58](http://www.uniprot.org/uniprot/Q96T58) [Eukaryota] Msx2-interacting protein [Metazoa][Homo sapiens] (length=3664 residues).

Site : 1488- 1493, Identity

kdkekvdsap_RPIPSW_ymkkkkirtd

--------------------------------------------------------------------------------

[NPSA](https://npsa-prabi.ibcp.fr/cgi-bin/seq_methonseq.pl?seqid=Q99062&db=unipsp&process=5be3cc137c874548&seqtype=1) [gnl|unipsp|Q99062](http://www.uniprot.org/uniprot/Q99062) [Eukaryota] Granulocyte colony-stimulating factor receptor [Metazoa][Homo sapiens] (length=836 residues).

Site : 654- 659, Identity

gtawlccspn_RKNPLW_psvpdpahss

--------------------------------------------------------------------------------

[NPSA](https://npsa-prabi.ibcp.fr/cgi-bin/seq_methonseq.pl?seqid=Q99572&db=unipsp&process=5be3cc137c874549&seqtype=1) [gnl|unipsp|Q99572](http://www.uniprot.org/uniprot/Q99572) [Eukaryota] P2X purinoceptor 7 [Metazoa][Homo sapiens] (length=595 residues).

Site : 471- 476, Identity

llrkeatprs_RDSPVW_cqcgsclpsq

--------------------------------------------------------------------------------

[NPSA](https://npsa-prabi.ibcp.fr/cgi-bin/seq_methonseq.pl?seqid=Q99665&db=unipsp&process=5be3cc137c874550&seqtype=1) [gnl|unipsp|Q99665](http://www.uniprot.org/uniprot/Q99665) [Eukaryota] Interleukin-12 receptor subunit beta-2 [Metazoa][Homo sapiens] (length=862 residues).

Site : 223- 228, Identity

pstftfldiv_RPLPPW_dirikfqkas

--------------------------------------------------------------------------------

[NPSA](https://npsa-prabi.ibcp.fr/cgi-bin/seq_methonseq.pl?seqid=Q99727&db=unipsp&process=5be3cc137c874551&seqtype=1) [gnl|unipsp|Q99727](http://www.uniprot.org/uniprot/Q99727) [Eukaryota] Metalloproteinase inhibitor 4 [Metazoa][Homo sapiens] (length=224 residues).

Site : 6- 11, Identity

‹mpgsp_RPAPSW_vlllrllall

--------------------------------------------------------------------------------

[NPSA](https://npsa-prabi.ibcp.fr/cgi-bin/seq_methonseq.pl?seqid=Q9BQ51&db=unipsp&process=5be3cc137c874552&seqtype=1) [gnl|unipsp|Q9BQ51](http://www.uniprot.org/uniprot/Q9BQ51) [Eukaryota] Programmed cell death 1 ligand 2 [Metazoa][Homo sapiens] (length=273 residues).

Site : 216- 221, Identity

sidlqsqmep_RTHPTW_llhifipfci

--------------------------------------------------------------------------------

[NPSA](https://npsa-prabi.ibcp.fr/cgi-bin/seq_methonseq.pl?seqid=Q9BQC3&db=unipsp&process=5be3cc137c874553&seqtype=1) [gnl|unipsp|Q9BQC3](http://www.uniprot.org/uniprot/Q9BQC3) [Eukaryota] Diphthamide biosynthesis protein 2 [Metazoa][Homo sapiens] (length=489 residues).

Site : 418- 423, Identity

pdvslitgdl_RPPPAW_kssndhgsla

--------------------------------------------------------------------------------

[NPSA](https://npsa-prabi.ibcp.fr/cgi-bin/seq_methonseq.pl?seqid=Q9BQY6&db=unipsp&process=5be3cc137c874554&seqtype=1) [gnl|unipsp|Q9BQY6](http://www.uniprot.org/uniprot/Q9BQY6) [Eukaryota] WAP four-disulfide core domain protein 6 [Metazoa][Homo sapiens] (length=131 residues).

Site : 82- 87, Identity

frkiyavchr_RLAPAW_ppyhtggtik

--------------------------------------------------------------------------------

[NPSA](https://npsa-prabi.ibcp.fr/cgi-bin/seq_methonseq.pl?seqid=Q9BRJ7&db=unipsp&process=5be3cc137c874555&seqtype=1) [gnl|unipsp|Q9BRJ7](http://www.uniprot.org/uniprot/Q9BRJ7) [Eukaryota] Protein syndesmos [Metazoa][Homo sapiens] (length=211 residues).

Site : 19- 24, Identity

lkqisrveam_RLGPGW_shschamlya

--------------------------------------------------------------------------------

[NPSA](https://npsa-prabi.ibcp.fr/cgi-bin/seq_methonseq.pl?seqid=Q9BSJ6&db=unipsp&process=5be3cc137c874556&seqtype=1) [gnl|unipsp|Q9BSJ6](http://www.uniprot.org/uniprot/Q9BSJ6) [Eukaryota] Protein FAM64A [Metazoa][Homo sapiens] (length=248 residues).

Site : 64- 69, Identity

lplravnlnl_RAGPSW_krletpepgq

--------------------------------------------------------------------------------

[NPSA](https://npsa-prabi.ibcp.fr/cgi-bin/seq_methonseq.pl?seqid=Q9BSJ8&db=unipsp&process=5be3cc137c874557&seqtype=1) [gnl|unipsp|Q9BSJ8](http://www.uniprot.org/uniprot/Q9BSJ8) [Eukaryota] Extended synaptotagmin-1 [Metazoa][Homo sapiens] (length=1104 residues).

Site : 125- 130, Identity

ltaktlymsh_RELPAW_vsfpdvekae

--------------------------------------------------------------------------------

[NPSA](https://npsa-prabi.ibcp.fr/cgi-bin/seq_methonseq.pl?seqid=Q9BTE6&db=unipsp&process=5be3cc137c874558&seqtype=1) [gnl|unipsp|Q9BTE6](http://www.uniprot.org/uniprot/Q9BTE6) [Eukaryota] Alanyl-tRNA editing protein Aarsd1 [Metazoa][Homo sapiens] (length=412 residues).

Site : 305- 310, Identity

dlavhiahsl_RNSPDW_ggvvilhrke

--------------------------------------------------------------------------------

[NPSA](https://npsa-prabi.ibcp.fr/cgi-bin/seq_methonseq.pl?seqid=Q9BTY2&db=unipsp&process=5be3cc137c874559&seqtype=1) [gnl|unipsp|Q9BTY2](http://www.uniprot.org/uniprot/Q9BTY2) [Eukaryota] Plasma alpha-L-fucosidase [Metazoa][Homo sapiens] (length=467 residues).

Site : 33- 38, Identity

pppcpahsat_RFDPTW_esldarqlpa

Site : 44- 49, Identity

fdptweslda_RQLPAW_fdqakfgifi

--------------------------------------------------------------------------------

[NPSA](https://npsa-prabi.ibcp.fr/cgi-bin/seq_methonseq.pl?seqid=Q9BV97&db=unipsp&process=5be3cc137c874560&seqtype=1) [gnl|unipsp|Q9BV97](http://www.uniprot.org/uniprot/Q9BV97) [Eukaryota] KRAB domain-containing protein ZNF747 [Metazoa][Homo sapiens] (length=191 residues).

Site : 131- 136, Identity

lpqeseaasr_RSSPGW_rrrpncgirl

--------------------------------------------------------------------------------

[NPSA](https://npsa-prabi.ibcp.fr/cgi-bin/seq_methonseq.pl?seqid=Q9BVK8&db=unipsp&process=5be3cc137c874561&seqtype=1) [gnl|unipsp|Q9BVK8](http://www.uniprot.org/uniprot/Q9BVK8) [Eukaryota] Transmembrane protein 147 [Metazoa][Homo sapiens] (length=224 residues).

Site : 115- 120, Identity

gwataelims_RCIPLW_vgargiefdw

--------------------------------------------------------------------------------

[NPSA](https://npsa-prabi.ibcp.fr/cgi-bin/seq_methonseq.pl?seqid=Q9BWE0&db=unipsp&process=5be3cc137c874562&seqtype=1) [gnl|unipsp|Q9BWE0](http://www.uniprot.org/uniprot/Q9BWE0) [Eukaryota] Replication initiator 1 [Metazoa][Homo sapiens] (length=567 residues).

Site : 64- 69, Identity

grahrcahcr_RHFPGW_valwlhtrrc

--------------------------------------------------------------------------------

[NPSA](https://npsa-prabi.ibcp.fr/cgi-bin/seq_methonseq.pl?seqid=Q9BWK5&db=unipsp&process=5be3cc137c874563&seqtype=1) [gnl|unipsp|Q9BWK5](http://www.uniprot.org/uniprot/Q9BWK5) [Eukaryota] Modulator of retrovirus infection homolog [Metazoa][Homo sapiens] (length=157 residues).

Site : 11- 16, Identity

metlqsetkt_RVLPSW_ltaqvatknv

--------------------------------------------------------------------------------

[NPSA](https://npsa-prabi.ibcp.fr/cgi-bin/seq_methonseq.pl?seqid=Q9BWN1&db=unipsp&process=5be3cc137c874564&seqtype=1) [gnl|unipsp|Q9BWN1](http://www.uniprot.org/uniprot/Q9BWN1) [Eukaryota] Proline-rich protein 14 [Metazoa][Homo sapiens] (length=585 residues).

Site : 579- 584, Identity

allleeetvd_REQPHW_t›

--------------------------------------------------------------------------------

[NPSA](https://npsa-prabi.ibcp.fr/cgi-bin/seq_methonseq.pl?seqid=Q9BWW9&db=unipsp&process=5be3cc137c874565&seqtype=1) [gnl|unipsp|Q9BWW9](http://www.uniprot.org/uniprot/Q9BWW9) [Eukaryota] Apolipoprotein L5 [Metazoa][Homo sapiens] (length=433 residues).

Site : 266- 271, Identity

mamvknfvak_RHIPFW_targvqrafe

--------------------------------------------------------------------------------

[NPSA](https://npsa-prabi.ibcp.fr/cgi-bin/seq_methonseq.pl?seqid=Q9BYC8&db=unipsp&process=5be3cc137c874566&seqtype=1) [gnl|unipsp|Q9BYC8](http://www.uniprot.org/uniprot/Q9BYC8) [Eukaryota] 39S ribosomal protein L32, mitochondrial [Metazoa][Homo sapiens] (length=188 residues).

Site : 179- 184, Identity

dqgkriierd_RKRPSW_ftqn›

--------------------------------------------------------------------------------

[NPSA](https://npsa-prabi.ibcp.fr/cgi-bin/seq_methonseq.pl?seqid=Q9BYK8&db=unipsp&process=5be3cc137c874567&seqtype=1) [gnl|unipsp|Q9BYK8](http://www.uniprot.org/uniprot/Q9BYK8) [Eukaryota] Helicase with zinc finger domain 2 [Metazoa][Homo sapiens] (length=2649 residues).

Site : 2615- 2620, Identity

lcligdhlll_RCCPLW_rslldfceaq

--------------------------------------------------------------------------------

[NPSA](https://npsa-prabi.ibcp.fr/cgi-bin/seq_methonseq.pl?seqid=Q9BYZ6&db=unipsp&process=5be3cc137c874568&seqtype=1) [gnl|unipsp|Q9BYZ6](http://www.uniprot.org/uniprot/Q9BYZ6) [Eukaryota] Rho-related BTB domain-containing protein 2 [Metazoa][Homo sapiens] (length=727 residues).

Site : 665- 670, Identity

penqeyfekh_RWPPVW_ylkeedhyqr

--------------------------------------------------------------------------------

[NPSA](https://npsa-prabi.ibcp.fr/cgi-bin/seq_methonseq.pl?seqid=Q9BYZ8&db=unipsp&process=5be3cc137c874569&seqtype=1) [gnl|unipsp|Q9BYZ8](http://www.uniprot.org/uniprot/Q9BYZ8) [Eukaryota] Regenerating islet-derived protein 4 [Metazoa][Homo sapiens] (length=158 residues).

Site : 88- 93, Identity

tiaeyisgyq_RSQPIW_iglhdpqkrq

--------------------------------------------------------------------------------

[NPSA](https://npsa-prabi.ibcp.fr/cgi-bin/seq_methonseq.pl?seqid=Q9BZM5&db=unipsp&process=5be3cc137c874570&seqtype=1) [gnl|unipsp|Q9BZM5](http://www.uniprot.org/uniprot/Q9BZM5) [Eukaryota] NKG2D ligand 2 [Metazoa][Homo sapiens] (length=246 residues).

Site : 44- 49, Identity

cyditvipkf_RPGPRW_cavqgqvdek

--------------------------------------------------------------------------------

[NPSA](https://npsa-prabi.ibcp.fr/cgi-bin/seq_methonseq.pl?seqid=Q9BZM6&db=unipsp&process=5be3cc137c874571&seqtype=1) [gnl|unipsp|Q9BZM6](http://www.uniprot.org/uniprot/Q9BZM6) [Eukaryota] NKG2D ligand 1 [Metazoa][Homo sapiens] (length=244 residues).

Site : 44- 49, Identity

cydfiitpks_RPEPQW_cevqglvder

--------------------------------------------------------------------------------

[NPSA](https://npsa-prabi.ibcp.fr/cgi-bin/seq_methonseq.pl?seqid=Q9BZR6&db=unipsp&process=5be3cc137c874572&seqtype=1) [gnl|unipsp|Q9BZR6](http://www.uniprot.org/uniprot/Q9BZR6) [Eukaryota] Reticulon-4 receptor [Metazoa][Homo sapiens] (length=473 residues).

Site : 267- 272, Identity

lndnpwvcdc_RARPLW_awlqkfrgss

--------------------------------------------------------------------------------

[NPSA](https://npsa-prabi.ibcp.fr/cgi-bin/seq_methonseq.pl?seqid=Q9GZN6&db=unipsp&process=5be3cc137c874573&seqtype=1) [gnl|unipsp|Q9GZN6](http://www.uniprot.org/uniprot/Q9GZN6) [Eukaryota] Orphan sodium- and chloride-dependent neurotransmitter transporter NTT5 [Metazoa][Homo sapiens] (length=736 residues).

Site : 653- 658, Identity

wdsstskevl_RPYPPW_alllmitlfa

--------------------------------------------------------------------------------

[NPSA](https://npsa-prabi.ibcp.fr/cgi-bin/seq_methonseq.pl?seqid=Q9GZZ6&db=unipsp&process=5be3cc137c874574&seqtype=1) [gnl|unipsp|Q9GZZ6](http://www.uniprot.org/uniprot/Q9GZZ6) [Eukaryota] Neuronal acetylcholine receptor subunit alpha-10 [Metazoa][Homo sapiens] (length=450 residues).

Site : 330- 335, Identity

mnlhycgpsv_RPVPAW_aralllghla

--------------------------------------------------------------------------------

[NPSA](https://npsa-prabi.ibcp.fr/cgi-bin/seq_methonseq.pl?seqid=Q9H0K4&db=unipsp&process=5be3cc137c874575&seqtype=1) [gnl|unipsp|Q9H0K4](http://www.uniprot.org/uniprot/Q9H0K4) [Eukaryota] Radial spoke head protein 6 homolog A [Metazoa][Homo sapiens] (length=717 residues).

Site : 52- 57, Identity

erqqippdaq_RNAPGW_sqrgslsqqe

--------------------------------------------------------------------------------

[NPSA](https://npsa-prabi.ibcp.fr/cgi-bin/seq_methonseq.pl?seqid=Q9H147&db=unipsp&process=5be3cc137c874576&seqtype=1) [gnl|unipsp|Q9H147](http://www.uniprot.org/uniprot/Q9H147) [Eukaryota] Deoxynucleotidyltransferase terminal-interacting protein 1 [Metazoa][Homo sapiens] (length=329 residues).

Site : 197- 202, Identity

wkpkscepir_REGPKW_dparlnestt

--------------------------------------------------------------------------------

[NPSA](https://npsa-prabi.ibcp.fr/cgi-bin/seq_methonseq.pl?seqid=Q9H172&db=unipsp&process=5be3cc137c874577&seqtype=1) [gnl|unipsp|Q9H172](http://www.uniprot.org/uniprot/Q9H172) [Eukaryota] ATP-binding cassette sub-family G member 4 [Metazoa][Homo sapiens] (length=646 residues).

Site : 71- 76, Identity

iefvelsysv_REGPCW_rkrgyktllk

--------------------------------------------------------------------------------

[NPSA](https://npsa-prabi.ibcp.fr/cgi-bin/seq_methonseq.pl?seqid=Q9H222&db=unipsp&process=5be3cc137c874578&seqtype=1) [gnl|unipsp|Q9H222](http://www.uniprot.org/uniprot/Q9H222) [Eukaryota] ATP-binding cassette sub-family G member 5 [Metazoa][Homo sapiens] (length=651 residues).

Site : 50- 55, Identity

ilhasysvsh_RVRPWW_ditscrqqwt

--------------------------------------------------------------------------------

[NPSA](https://npsa-prabi.ibcp.fr/cgi-bin/seq_methonseq.pl?seqid=Q9H361&db=unipsp&process=5be3cc137c874579&seqtype=1) [gnl|unipsp|Q9H361](http://www.uniprot.org/uniprot/Q9H361) [Eukaryota] Polyadenylate-binding protein 3 [Metazoa][Homo sapiens] (length=631 residues).

Site : 426- 431, Identity

yyppsqiarl_RPSPRW_taqgarphpf

--------------------------------------------------------------------------------

[NPSA](https://npsa-prabi.ibcp.fr/cgi-bin/seq_methonseq.pl?seqid=Q9H3S1&db=unipsp&process=5be3cc137c874580&seqtype=1) [gnl|unipsp|Q9H3S1](http://www.uniprot.org/uniprot/Q9H3S1) [Eukaryota] Semaphorin-4A [Metazoa][Homo sapiens] (length=761 residues).

Site : 540- 545, Identity

nlnswkqdme_RGNPEW_acasgpmsrs

--------------------------------------------------------------------------------

[NPSA](https://npsa-prabi.ibcp.fr/cgi-bin/seq_methonseq.pl?seqid=Q9H3S3&db=unipsp&process=5be3cc137c874581&seqtype=1) [gnl|unipsp|Q9H3S3](http://www.uniprot.org/uniprot/Q9H3S3) [Eukaryota] Transmembrane protease serine 5 [Metazoa][Homo sapiens] (length=457 residues).

Site : 126- 131, Identity

sedflleaqv_RDQPRW_llvchegwsp

--------------------------------------------------------------------------------

[NPSA](https://npsa-prabi.ibcp.fr/cgi-bin/seq_methonseq.pl?seqid=Q9H4L4&db=unipsp&process=5be3cc137c874582&seqtype=1) [gnl|unipsp|Q9H4L4](http://www.uniprot.org/uniprot/Q9H4L4) [Eukaryota] Sentrin-specific protease 3 [Metazoa][Homo sapiens] (length=574 residues).

Site : 97- 102, Identity

edeeeevaaw_RLPPRW_sqlgtsqrpr

--------------------------------------------------------------------------------

[NPSA](https://npsa-prabi.ibcp.fr/cgi-bin/seq_methonseq.pl?seqid=Q9H4Y5&db=unipsp&process=5be3cc137c874583&seqtype=1) [gnl|unipsp|Q9H4Y5](http://www.uniprot.org/uniprot/Q9H4Y5) [Eukaryota] Glutathione S-transferase omega-2 [Metazoa][Homo sapiens] (length=243 residues).

Site : 57- 62, Identity

irhevvninl_RNKPEW_yytkhpfghi

--------------------------------------------------------------------------------

[NPSA](https://npsa-prabi.ibcp.fr/cgi-bin/seq_methonseq.pl?seqid=Q9H6V9&db=unipsp&process=5be3cc137c874584&seqtype=1) [gnl|unipsp|Q9H6V9](http://www.uniprot.org/uniprot/Q9H6V9) [Eukaryota] Lipid droplet-associated hydrolase [Metazoa][Homo sapiens] (length=325 residues).

Site : 73- 78, Identity

fakalysltn_RRFPVW_tishaghala

--------------------------------------------------------------------------------

[NPSA](https://npsa-prabi.ibcp.fr/cgi-bin/seq_methonseq.pl?seqid=Q9H8E8&db=unipsp&process=5be3cc137c874585&seqtype=1) [gnl|unipsp|Q9H8E8](http://www.uniprot.org/uniprot/Q9H8E8) [Eukaryota] Cysteine-rich protein 2-binding protein [Metazoa][Homo sapiens] (length=782 residues).

Site : 625- 630, Identity

llsqirshlh_RSDPHW_tpepdapldy

--------------------------------------------------------------------------------

[NPSA](https://npsa-prabi.ibcp.fr/cgi-bin/seq_methonseq.pl?seqid=Q9H8S5&db=unipsp&process=5be3cc137c874586&seqtype=1) [gnl|unipsp|Q9H8S5](http://www.uniprot.org/uniprot/Q9H8S5) [Eukaryota] Cyclin N-terminal domain-containing protein 2 [Metazoa][Homo sapiens] (length=307 residues).

Site : 290- 295, Identity

ggsvwghrsf_RDLPSW_sflrsrrmrd

--------------------------------------------------------------------------------

[NPSA](https://npsa-prabi.ibcp.fr/cgi-bin/seq_methonseq.pl?seqid=Q9HB07&db=unipsp&process=5be3cc137c874587&seqtype=1) [gnl|unipsp|Q9HB07](http://www.uniprot.org/uniprot/Q9HB07) [Eukaryota] UPF0160 protein MYG1, mitochondrial [Metazoa][Homo sapiens] (length=376 residues).

Site : 201- 206, Identity

ltttlsarva_RLNPTW_nhpdqdteag

--------------------------------------------------------------------------------

[NPSA](https://npsa-prabi.ibcp.fr/cgi-bin/seq_methonseq.pl?seqid=Q9HC10&db=unipsp&process=5be3cc137c874588&seqtype=1) [gnl|unipsp|Q9HC10](http://www.uniprot.org/uniprot/Q9HC10) [Eukaryota] Otoferlin [Metazoa][Homo sapiens] (length=1997 residues).

Site : 1236- 1241, Identity

lrrfiyrppd_RSAPSW_nttvrllrrc

--------------------------------------------------------------------------------

[NPSA](https://npsa-prabi.ibcp.fr/cgi-bin/seq_methonseq.pl?seqid=Q9HC84&db=unipsp&process=5be3cc137c874589&seqtype=1) [gnl|unipsp|Q9HC84](http://www.uniprot.org/uniprot/Q9HC84) [Eukaryota] Mucin-5B [Metazoa][Homo sapiens] (length=5762 residues).

Site : 2628- 2633, Identity

atpssspgta_RTLPVW_isttttpttr

Site : 3885- 3890, Identity

vtpssspgta_RTPPVW_isttttptts

--------------------------------------------------------------------------------

[NPSA](https://npsa-prabi.ibcp.fr/cgi-bin/seq_methonseq.pl?seqid=Q9HCG7&db=unipsp&process=5be3cc137c874590&seqtype=1) [gnl|unipsp|Q9HCG7](http://www.uniprot.org/uniprot/Q9HCG7) [Eukaryota] Non-lysosomal glucosylceramidase [Metazoa][Homo sapiens] (length=927 residues).

Site : 470- 475, Identity

sawqspvldd_RSLPAW_yksalfnely

--------------------------------------------------------------------------------

[NPSA](https://npsa-prabi.ibcp.fr/cgi-bin/seq_methonseq.pl?seqid=Q9HCJ0&db=unipsp&process=5be3cc137c874591&seqtype=1) [gnl|unipsp|Q9HCJ0](http://www.uniprot.org/uniprot/Q9HCJ0) [Eukaryota] Trinucleotide repeat-containing gene 6C protein [Metazoa][Homo sapiens] (length=1690 residues).

Site : 587- 592, Identity

pksqhwgdgq_RSNPAW_sagggdwads

--------------------------------------------------------------------------------

[NPSA](https://npsa-prabi.ibcp.fr/cgi-bin/seq_methonseq.pl?seqid=Q9HCJ5&db=unipsp&process=5be3cc137c874592&seqtype=1) [gnl|unipsp|Q9HCJ5](http://www.uniprot.org/uniprot/Q9HCJ5) [Eukaryota] Zinc finger SWIM domain-containing protein 6 [Metazoa][Homo sapiens] (length=1215 residues).

Site : 557- 562, Identity

ddtenslfds_RGWPLW_hehvptacar

--------------------------------------------------------------------------------

[NPSA](https://npsa-prabi.ibcp.fr/cgi-bin/seq_methonseq.pl?seqid=Q9HCM2&db=unipsp&process=5be3cc137c874593&seqtype=1) [gnl|unipsp|Q9HCM2](http://www.uniprot.org/uniprot/Q9HCM2) [Eukaryota] Plexin-A4 [Metazoa][Homo sapiens] (length=1894 residues).

Site : 1044- 1049, Identity

fqyvedptiv_RIEPEW_sivsgntpia

--------------------------------------------------------------------------------

[NPSA](https://npsa-prabi.ibcp.fr/cgi-bin/seq_methonseq.pl?seqid=Q9HCU5&db=unipsp&process=5be3cc137c874594&seqtype=1) [gnl|unipsp|Q9HCU5](http://www.uniprot.org/uniprot/Q9HCU5) [Eukaryota] Prolactin regulatory element-binding protein [Metazoa][Homo sapiens] (length=417 residues).

Site : 387- 392, Identity

rcqlhllpsr_RSVPVW_lllllcvgli

--------------------------------------------------------------------------------

[NPSA](https://npsa-prabi.ibcp.fr/cgi-bin/seq_methonseq.pl?seqid=Q9NP87&db=unipsp&process=5be3cc137c874595&seqtype=1) [gnl|unipsp|Q9NP87](http://www.uniprot.org/uniprot/Q9NP87) [Eukaryota] DNA-directed DNA/RNA polymerase mu [Metazoa][Homo sapiens] (length=494 residues).

Site : 407- 412, Identity

ppgaavggst_RPCPSW_kavrvdlvva

--------------------------------------------------------------------------------

[NPSA](https://npsa-prabi.ibcp.fr/cgi-bin/seq_methonseq.pl?seqid=Q9NQ11&db=unipsp&process=5be3cc137c874596&seqtype=1) [gnl|unipsp|Q9NQ11](http://www.uniprot.org/uniprot/Q9NQ11) [Eukaryota] Probable cation-transporting ATPase 13A2 [Metazoa][Homo sapiens] (length=1180 residues).

Site : 66- 71, Identity

mmagiplllf_RWKPLW_gvrlrlrpcn

--------------------------------------------------------------------------------

[NPSA](https://npsa-prabi.ibcp.fr/cgi-bin/seq_methonseq.pl?seqid=Q9NQ29&db=unipsp&process=5be3cc137c874597&seqtype=1) [gnl|unipsp|Q9NQ29](http://www.uniprot.org/uniprot/Q9NQ29) [Eukaryota] Putative RNA-binding protein Luc7-like 1 [Metazoa][Homo sapiens] (length=371 residues).

Site : 344- 349, Identity

eeswesgrse_RGPPDW_rlessngkma

--------------------------------------------------------------------------------

[NPSA](https://npsa-prabi.ibcp.fr/cgi-bin/seq_methonseq.pl?seqid=Q9NRD8&db=unipsp&process=5be3cc137c874598&seqtype=1) [gnl|unipsp|Q9NRD8](http://www.uniprot.org/uniprot/Q9NRD8) [Eukaryota] Dual oxidase 2 [Metazoa][Homo sapiens] (length=1548 residues).

*****› PATTERN 1

Site : 271- 276, Identity

yhnlwaqrla_RQHPDW_edeelfqhar

--------------------------------------------------------------------------------

[NPSA](https://npsa-prabi.ibcp.fr/cgi-bin/seq_methonseq.pl?seqid=Q9NRD9&db=unipsp&process=5be3cc137c874599&seqtype=1) [gnl|unipsp|Q9NRD9](http://www.uniprot.org/uniprot/Q9NRD9) [Eukaryota] Dual oxidase 1 [Metazoa][Homo sapiens] (length=1551 residues).

Site : 265- 270, Identity

yhnlwaqrla_RQHPDW_edeelfqhar

--------------------------------------------------------------------------------

[NPSA](https://npsa-prabi.ibcp.fr/cgi-bin/seq_methonseq.pl?seqid=Q9NRK6&db=unipsp&process=5be3cc137c874600&seqtype=1) [gnl|unipsp|Q9NRK6](http://www.uniprot.org/uniprot/Q9NRK6) [Eukaryota] ATP-binding cassette sub-family B member 10, mitochondrial [Metazoa][Homo sapiens] (length=738 residues).

Site : 2- 7, Identity

‹m_RGPPAW_plrlleppsp

--------------------------------------------------------------------------------

[NPSA](https://npsa-prabi.ibcp.fr/cgi-bin/seq_methonseq.pl?seqid=Q9NS84&db=unipsp&process=5be3cc137c874601&seqtype=1) [gnl|unipsp|Q9NS84](http://www.uniprot.org/uniprot/Q9NS84) [Eukaryota] Carbohydrate sulfotransferase 7 [Metazoa][Homo sapiens] (length=486 residues).

Site : 360- 365, Identity

eawlrdllfa_RGAPAW_lrrrylrlry

--------------------------------------------------------------------------------

[NPSA](https://npsa-prabi.ibcp.fr/cgi-bin/seq_methonseq.pl?seqid=Q9NU39&db=unipsp&process=5be3cc137c874602&seqtype=1) [gnl|unipsp|Q9NU39](http://www.uniprot.org/uniprot/Q9NU39) [Eukaryota] Forkhead box protein D4-like 1 [Metazoa][Homo sapiens] (length=408 residues).

Site : 146- 151, Identity

fisgrfpyyr_RKFPAW_qnsirhnlsl

--------------------------------------------------------------------------------

[NPSA](https://npsa-prabi.ibcp.fr/cgi-bin/seq_methonseq.pl?seqid=Q9NWH9&db=unipsp&process=5be3cc137c874603&seqtype=1) [gnl|unipsp|Q9NWH9](http://www.uniprot.org/uniprot/Q9NWH9) [Eukaryota] SAFB-like transcription modulator [Metazoa][Homo sapiens] (length=1034 residues).

Site : 736- 741, Identity

lkrprdvdhr_RDDPYW_senkklsldt

--------------------------------------------------------------------------------

[NPSA](https://npsa-prabi.ibcp.fr/cgi-bin/seq_methonseq.pl?seqid=Q9NXD2&db=unipsp&process=5be3cc137c874604&seqtype=1) [gnl|unipsp|Q9NXD2](http://www.uniprot.org/uniprot/Q9NXD2) [Eukaryota] Myotubularin-related protein 10 [Metazoa][Homo sapiens] (length=777 residues).

Site : 279- 284, Identity

lkifshsfvg_RRMPLW_cwshsngsal

--------------------------------------------------------------------------------

[NPSA](https://npsa-prabi.ibcp.fr/cgi-bin/seq_methonseq.pl?seqid=Q9NXH8&db=unipsp&process=5be3cc137c874605&seqtype=1) [gnl|unipsp|Q9NXH8](http://www.uniprot.org/uniprot/Q9NXH8) [Eukaryota] Torsin-4A [Metazoa][Homo sapiens] (length=423 residues).

Site : 350- 355, Identity

lrasllavls_REHPLW_qaaaivpfll

--------------------------------------------------------------------------------

[NPSA](https://npsa-prabi.ibcp.fr/cgi-bin/seq_methonseq.pl?seqid=Q9NYF3&db=unipsp&process=5be3cc137c874606&seqtype=1) [gnl|unipsp|Q9NYF3](http://www.uniprot.org/uniprot/Q9NYF3) [Eukaryota] Protein FAM53C [Metazoa][Homo sapiens] (length=392 residues).

Site : 129- 134, Identity

rslsvpvdls_RWQPVW_rpapsklwtp

--------------------------------------------------------------------------------

[NPSA](https://npsa-prabi.ibcp.fr/cgi-bin/seq_methonseq.pl?seqid=Q9NYP8&db=unipsp&process=5be3cc137c874607&seqtype=1) [gnl|unipsp|Q9NYP8](http://www.uniprot.org/uniprot/Q9NYP8) [Eukaryota] Uncharacterized protein C21orf62 [Metazoa][Homo sapiens] (length=219 residues).

Site : 151- 156, Identity

lihsggdsds_REKPMW_lhkgwqpcmy

--------------------------------------------------------------------------------

[NPSA](https://npsa-prabi.ibcp.fr/cgi-bin/seq_methonseq.pl?seqid=Q9NYU1&db=unipsp&process=5be3cc137c874608&seqtype=1) [gnl|unipsp|Q9NYU1](http://www.uniprot.org/uniprot/Q9NYU1) [Eukaryota] UDP-glucose:glycoprotein glucosyltransferase 2 [Metazoa][Homo sapiens] (length=1516 residues).

Site : 1483- 1488, Identity

tkesklkaaa_RIVPEW_veydaeirql

--------------------------------------------------------------------------------

[NPSA](https://npsa-prabi.ibcp.fr/cgi-bin/seq_methonseq.pl?seqid=Q9NYU2&db=unipsp&process=5be3cc137c874609&seqtype=1) [gnl|unipsp|Q9NYU2](http://www.uniprot.org/uniprot/Q9NYU2) [Eukaryota] UDP-glucose:glycoprotein glucosyltransferase 1 [Metazoa][Homo sapiens] (length=1555 residues).

Site : 1508- 1513, Identity

tkepkleaav_RIVPEW_qdydqeikql

--------------------------------------------------------------------------------

[NPSA](https://npsa-prabi.ibcp.fr/cgi-bin/seq_methonseq.pl?seqid=Q9NZW5&db=unipsp&process=5be3cc137c874610&seqtype=1) [gnl|unipsp|Q9NZW5](http://www.uniprot.org/uniprot/Q9NZW5) [Eukaryota] MAGUK p55 subfamily member 6 [Metazoa][Homo sapiens] (length=540 residues).

Site : 254- 259, Identity

skgeilqivn_REDPNW_wqashvkegg

Site : 528- 533, Identity

eklqtaiekl_RMEPQW_vpiswvy›

--------------------------------------------------------------------------------

[NPSA](https://npsa-prabi.ibcp.fr/cgi-bin/seq_methonseq.pl?seqid=Q9NZY2&db=unipsp&process=5be3cc137c874611&seqtype=1) [gnl|unipsp|Q9NZY2](http://www.uniprot.org/uniprot/Q9NZY2) [Eukaryota] Putative uncharacterized protein KIAA0125 [Metazoa][Homo sapiens] (length=134 residues).

Site : 12- 17, Identity

gtlqgaalrs_RERPSW_pqethghrer

--------------------------------------------------------------------------------

[NPSA](https://npsa-prabi.ibcp.fr/cgi-bin/seq_methonseq.pl?seqid=Q9P1Z0&db=unipsp&process=5be3cc137c874612&seqtype=1) [gnl|unipsp|Q9P1Z0](http://www.uniprot.org/uniprot/Q9P1Z0) [Eukaryota] Zinc finger and BTB domain-containing protein 4 [Metazoa][Homo sapiens] (length=1013 residues).

1

Site : 693- 698, Identity

svggsglprg_RRPPRW_rqklerrswe

--------------------------------------------------------------------------------

[NPSA](https://npsa-prabi.ibcp.fr/cgi-bin/seq_methonseq.pl?seqid=Q9P2Q2&db=unipsp&process=5be3cc137c874613&seqtype=1) [gnl|unipsp|Q9P2Q2](http://www.uniprot.org/uniprot/Q9P2Q2) [Eukaryota] FERM domain-containing protein 4A [Metazoa][Homo sapiens] (length=1039 residues).

Site : 660- 665, Identity

gsnslqnspi_RGLPHW_nsqssmpstp

--------------------------------------------------------------------------------

[NPSA](https://npsa-prabi.ibcp.fr/cgi-bin/seq_methonseq.pl?seqid=Q9P2T0&db=unipsp&process=5be3cc137c874614&seqtype=1) [gnl|unipsp|Q9P2T0](http://www.uniprot.org/uniprot/Q9P2T0) [Eukaryota] Testicular haploid expressed gene protein [Metazoa][Homo sapiens] (length=379 residues).

Site : 212- 217, Identity

rfyleyynnn_RTTPVW_piprssleyr

--------------------------------------------------------------------------------

[NPSA](https://npsa-prabi.ibcp.fr/cgi-bin/seq_methonseq.pl?seqid=Q9UBL3&db=unipsp&process=5be3cc137c874615&seqtype=1) [gnl|unipsp|Q9UBL3](http://www.uniprot.org/uniprot/Q9UBL3) [Eukaryota] Set1/Ash2 histone methyltransferase complex subunit ASH2 [Metazoa][Homo sapiens] (length=628 residues).

Site : 621- 626, Identity

lyhvetevdg_RRSPPW_ep›

--------------------------------------------------------------------------------

[NPSA](https://npsa-prabi.ibcp.fr/cgi-bin/seq_methonseq.pl?seqid=Q9UBR2&db=unipsp&process=5be3cc137c874616&seqtype=1) [gnl|unipsp|Q9UBR2](http://www.uniprot.org/uniprot/Q9UBR2) [Eukaryota] Cathepsin Z [Metazoa][Homo sapiens] (length=303 residues).

Site : 3- 8, Identity

‹ma_RRGPGW_rpllllvlla

--------------------------------------------------------------------------------

[NPSA](https://npsa-prabi.ibcp.fr/cgi-bin/seq_methonseq.pl?seqid=Q9UGI9&db=unipsp&process=5be3cc137c874617&seqtype=1) [gnl|unipsp|Q9UGI9](http://www.uniprot.org/uniprot/Q9UGI9) [Eukaryota] 5'-AMP-activated protein kinase subunit gamma-3 [Metazoa][Homo sapiens] (length=489 residues).

Site : 10- 15, Identity

‹mepglehal_RRTPSW_sslggsehqe

Site : 225- 230, Identity

affalvangv_RAAPLW_dskkqsfvgm

--------------------------------------------------------------------------------

[NPSA](https://npsa-prabi.ibcp.fr/cgi-bin/seq_methonseq.pl?seqid=Q9UGJ0&db=unipsp&process=5be3cc137c874618&seqtype=1) [gnl|unipsp|Q9UGJ0](http://www.uniprot.org/uniprot/Q9UGJ0) [Eukaryota] 5'-AMP-activated protein kinase subunit gamma-2 [Metazoa][Homo sapiens] (length=569 residues).

Site : 302- 307, Identity

affalvangv_RAAPLW_eskkqsfvgm

--------------------------------------------------------------------------------

[NPSA](https://npsa-prabi.ibcp.fr/cgi-bin/seq_methonseq.pl?seqid=Q9UGL1&db=unipsp&process=5be3cc137c874619&seqtype=1) [gnl|unipsp|Q9UGL1](http://www.uniprot.org/uniprot/Q9UGL1) [Eukaryota] Lysine-specific demethylase 5B [Metazoa][Homo sapiens] (length=1544 residues).

Site : 65- 70, Identity

iaeqtgickv_RPPPDW_qppfacdvdk

--------------------------------------------------------------------------------

[NPSA](https://npsa-prabi.ibcp.fr/cgi-bin/seq_methonseq.pl?seqid=Q9UGM1&db=unipsp&process=5be3cc137c874620&seqtype=1) [gnl|unipsp|Q9UGM1](http://www.uniprot.org/uniprot/Q9UGM1) [Eukaryota] Neuronal acetylcholine receptor subunit alpha-9 [Metazoa][Homo sapiens] (length=479 residues).

Site : 331- 336, Identity

mnihfcgaea_RPVPHW_arvvilkyms

--------------------------------------------------------------------------------

[NPSA](https://npsa-prabi.ibcp.fr/cgi-bin/seq_methonseq.pl?seqid=Q9UHR6&db=unipsp&process=5be3cc137c874621&seqtype=1) [gnl|unipsp|Q9UHR6](http://www.uniprot.org/uniprot/Q9UHR6) [Eukaryota] Zinc finger HIT domain-containing protein 2 [Metazoa][Homo sapiens] (length=403 residues).

Site : 121- 126, Identity

erllsrgeag_RLLPPW_rpwwwnrgag

--------------------------------------------------------------------------------

[NPSA](https://npsa-prabi.ibcp.fr/cgi-bin/seq_methonseq.pl?seqid=Q9UHX3&db=unipsp&process=5be3cc137c874622&seqtype=1) [gnl|unipsp|Q9UHX3](http://www.uniprot.org/uniprot/Q9UHX3) [Eukaryota] Adhesion G protein-coupled receptor E2 [Metazoa][Homo sapiens] (length=823 residues).

Site : 190- 195, Identity

clnnvgsyqc_RCRPGW_qpipgspngp

Site : 239- 244, Identity

cfntvgsysc_RCRPGW_kprhgipnnq

--------------------------------------------------------------------------------

[NPSA](https://npsa-prabi.ibcp.fr/cgi-bin/seq_methonseq.pl?seqid=Q9UI15&db=unipsp&process=5be3cc137c874623&seqtype=1) [gnl|unipsp|Q9UI15](http://www.uniprot.org/uniprot/Q9UI15) [Eukaryota] Transgelin-3 [Metazoa][Homo sapiens] (length=199 residues).

Site : 145- 150, Identity

vavtkddgcy_RGEPSW_fhrkaqqnrr

--------------------------------------------------------------------------------

[NPSA](https://npsa-prabi.ibcp.fr/cgi-bin/seq_methonseq.pl?seqid=Q9UIW2&db=unipsp&process=5be3cc137c874624&seqtype=1) [gnl|unipsp|Q9UIW2](http://www.uniprot.org/uniprot/Q9UIW2) [Eukaryota] Plexin-A1 [Metazoa][Homo sapiens] (length=1896 residues).

Site : 1052- 1057, Identity

ynytedptil_RIDPEW_sinsggtllt

--------------------------------------------------------------------------------

[NPSA](https://npsa-prabi.ibcp.fr/cgi-bin/seq_methonseq.pl?seqid=Q9UKB1&db=unipsp&process=5be3cc137c874625&seqtype=1) [gnl|unipsp|Q9UKB1](http://www.uniprot.org/uniprot/Q9UKB1) [Eukaryota] F-box/WD repeat-containing protein 11 [Metazoa][Homo sapiens] (length=542 residues).

Site : 173- 178, Identity

lwkkliermv_RTDPLW_kglserrgwd

--------------------------------------------------------------------------------

[NPSA](https://npsa-prabi.ibcp.fr/cgi-bin/seq_methonseq.pl?seqid=Q9UKN7&db=unipsp&process=5be3cc137c874626&seqtype=1) [gnl|unipsp|Q9UKN7](http://www.uniprot.org/uniprot/Q9UKN7) [Eukaryota] Unconventional myosin-XV [Metazoa][Homo sapiens] (length=3530 residues).

Site : 699- 704, Identity

rpaspygslr_RHPPPW_aapahvppap

--------------------------------------------------------------------------------

[NPSA](https://npsa-prabi.ibcp.fr/cgi-bin/seq_methonseq.pl?seqid=Q9UKT5&db=unipsp&process=5be3cc137c874627&seqtype=1) [gnl|unipsp|Q9UKT5](http://www.uniprot.org/uniprot/Q9UKT5) [Eukaryota] F-box only protein 4 [Metazoa][Homo sapiens] (length=387 residues).

Site : 105- 110, Identity

dpilwryfll_RDLPSW_ssvdwkslpd

--------------------------------------------------------------------------------

[NPSA](https://npsa-prabi.ibcp.fr/cgi-bin/seq_methonseq.pl?seqid=Q9UL54&db=unipsp&process=5be3cc137c874628&seqtype=1) [gnl|unipsp|Q9UL54](http://www.uniprot.org/uniprot/Q9UL54) [Eukaryota] Serine/threonine-protein kinase TAO2 [Metazoa][Homo sapiens] (length=1235 residues).

Site : 1229- 1234, Identity

agrrsrtrqs_RALPPW_r›

--------------------------------------------------------------------------------

[NPSA](https://npsa-prabi.ibcp.fr/cgi-bin/seq_methonseq.pl?seqid=Q9ULB4&db=unipsp&process=5be3cc137c874629&seqtype=1) [gnl|unipsp|Q9ULB4](http://www.uniprot.org/uniprot/Q9ULB4) [Eukaryota] Cadherin-9 [Metazoa][Homo sapiens] (length=789 residues).

Site : 702- 707, Identity

vmpetifqir_RTVPLW_enidvqdfih

--------------------------------------------------------------------------------

[NPSA](https://npsa-prabi.ibcp.fr/cgi-bin/seq_methonseq.pl?seqid=Q9ULD0&db=unipsp&process=5be3cc137c874630&seqtype=1) [gnl|unipsp|Q9ULD0](http://www.uniprot.org/uniprot/Q9ULD0) [Eukaryota] 2-oxoglutarate dehydrogenase-like, mitochondrial [Metazoa][Homo sapiens] (length=1010 residues).

Site : 965- 970, Identity

isprfmtilr_RARPIW_yvgrdpaaap

--------------------------------------------------------------------------------

[NPSA](https://npsa-prabi.ibcp.fr/cgi-bin/seq_methonseq.pl?seqid=Q9ULD5&db=unipsp&process=5be3cc137c874631&seqtype=1) [gnl|unipsp|Q9ULD5](http://www.uniprot.org/uniprot/Q9ULD5) [Eukaryota] Zinc finger protein 777 [Metazoa][Homo sapiens] (length=760 residues).

Site : 748- 753, Identity

tnhcrvhsre_RPPPRW_sssfcsl›

--------------------------------------------------------------------------------

[NPSA](https://npsa-prabi.ibcp.fr/cgi-bin/seq_methonseq.pl?seqid=Q9ULI3&db=unipsp&process=5be3cc137c874632&seqtype=1) [gnl|unipsp|Q9ULI3](http://www.uniprot.org/uniprot/Q9ULI3) [Eukaryota] Protein HEG homolog 1 [Metazoa][Homo sapiens] (length=1381 residues).

Site : 1012- 1017, Identity

adntsrgyhc_RCPPSW_qgddcsvdvn

--------------------------------------------------------------------------------

[NPSA](https://npsa-prabi.ibcp.fr/cgi-bin/seq_methonseq.pl?seqid=Q9ULL0&db=unipsp&process=5be3cc137c874633&seqtype=1) [gnl|unipsp|Q9ULL0](http://www.uniprot.org/uniprot/Q9ULL0) [Eukaryota] Uncharacterized protein KIAA1210 [Metazoa][Homo sapiens] (length=1709 residues).

Site : 424- 429, Identity

kqkknlqvii_RGLPVW_fshfqgileg

--------------------------------------------------------------------------------

[NPSA](https://npsa-prabi.ibcp.fr/cgi-bin/seq_methonseq.pl?seqid=Q9UNS1&db=unipsp&process=5be3cc137c874634&seqtype=1) [gnl|unipsp|Q9UNS1](http://www.uniprot.org/uniprot/Q9UNS1) [Eukaryota] Protein timeless homolog [Metazoa][Homo sapiens] (length=1208 residues).

Site : 817- 822, Identity

ygslddrsss_RRAPTW_speeeahlre

--------------------------------------------------------------------------------

[NPSA](https://npsa-prabi.ibcp.fr/cgi-bin/seq_methonseq.pl?seqid=Q9UPR6&db=unipsp&process=5be3cc137c874635&seqtype=1) [gnl|unipsp|Q9UPR6](http://www.uniprot.org/uniprot/Q9UPR6) [Eukaryota] Zinc finger RNA-binding protein 2 [Metazoa][Homo sapiens] (length=939 residues).

Site : 800- 805, Identity

ivirvlrdlc_RRVPTW_galpawamel

--------------------------------------------------------------------------------

[NPSA](https://npsa-prabi.ibcp.fr/cgi-bin/seq_methonseq.pl?seqid=Q9UPX6&db=unipsp&process=5be3cc137c874636&seqtype=1) [gnl|unipsp|Q9UPX6](http://www.uniprot.org/uniprot/Q9UPX6) [Eukaryota] UPF0258 protein KIAA1024 [Metazoa][Homo sapiens] (length=916 residues).

Site : 825- 830, Identity

mrltelaevk_RGQPSW_tieeyarnag

--------------------------------------------------------------------------------

[NPSA](https://npsa-prabi.ibcp.fr/cgi-bin/seq_methonseq.pl?seqid=Q9UPX8&db=unipsp&process=5be3cc137c874637&seqtype=1) [gnl|unipsp|Q9UPX8](http://www.uniprot.org/uniprot/Q9UPX8) [Eukaryota] SH3 and multiple ankyrin repeat domains protein 2 [Metazoa][Homo sapiens] (length=1470 residues).

Site : 111- 116, Identity

rlggagedgk_RPQPLW_hvgspfalga

--------------------------------------------------------------------------------

[NPSA](https://npsa-prabi.ibcp.fr/cgi-bin/seq_methonseq.pl?seqid=Q9Y2G5&db=unipsp&process=5be3cc137c874638&seqtype=1) [gnl|unipsp|Q9Y2G5](http://www.uniprot.org/uniprot/Q9Y2G5) [Eukaryota] GDP-fucose protein O-fucosyltransferase 2 [Metazoa][Homo sapiens] (length=429 residues).

Site : 351- 356, Identity

elkkllpemv_RFEPTW_eelelykdgg

--------------------------------------------------------------------------------

[NPSA](https://npsa-prabi.ibcp.fr/cgi-bin/seq_methonseq.pl?seqid=Q9Y2H5&db=unipsp&process=5be3cc137c874639&seqtype=1) [gnl|unipsp|Q9Y2H5](http://www.uniprot.org/uniprot/Q9Y2H5) [Eukaryota] Pleckstrin homology domain-containing family A member 6 [Metazoa][Homo sapiens] (length=1048 residues).

Site : 382- 387, Identity

esicsmpayd_RISPPW_aledkrhafr

--------------------------------------------------------------------------------

[NPSA](https://npsa-prabi.ibcp.fr/cgi-bin/seq_methonseq.pl?seqid=Q9Y2I1&db=unipsp&process=5be3cc137c874640&seqtype=1) [gnl|unipsp|Q9Y2I1](http://www.uniprot.org/uniprot/Q9Y2I1) [Eukaryota] Nischarin [Metazoa][Homo sapiens] (length=1504 residues).

Site : 616- 621, Identity

lstlirqaie_RQLPAW_ieaanqreeg

--------------------------------------------------------------------------------

[NPSA](https://npsa-prabi.ibcp.fr/cgi-bin/seq_methonseq.pl?seqid=Q9Y2I6&db=unipsp&process=5be3cc137c874641&seqtype=1) [gnl|unipsp|Q9Y2I6](http://www.uniprot.org/uniprot/Q9Y2I6) [Eukaryota] Ninein-like protein [Metazoa][Homo sapiens] (length=1382 residues).

Site : 583- 588, Identity

eglwarlpkn_RHSPSW_spdgrrrqlp

--------------------------------------------------------------------------------

[NPSA](https://npsa-prabi.ibcp.fr/cgi-bin/seq_methonseq.pl?seqid=Q9Y375&db=unipsp&process=5be3cc137c874642&seqtype=1) [gnl|unipsp|Q9Y375](http://www.uniprot.org/uniprot/Q9Y375) [Eukaryota] Complex I intermediate-associated protein 30, mitochondrial [Metazoa][Homo sapiens] (length=327 residues).

Site : 244- 249, Identity

nqmysyfmft_RGGPYW_qevkipfskf

--------------------------------------------------------------------------------

[NPSA](https://npsa-prabi.ibcp.fr/cgi-bin/seq_methonseq.pl?seqid=Q9Y490&db=unipsp&process=5be3cc137c874643&seqtype=1) [gnl|unipsp|Q9Y490](http://www.uniprot.org/uniprot/Q9Y490) [Eukaryota] Talin-1 [Metazoa][Homo sapiens] (length=2541 residues).

Site : 1625- 1630, Identity

qtaralavnp_RDPPSW_svlaghsrtv

--------------------------------------------------------------------------------

[NPSA](https://npsa-prabi.ibcp.fr/cgi-bin/seq_methonseq.pl?seqid=Q9Y4A5&db=unipsp&process=5be3cc137c874644&seqtype=1) [gnl|unipsp|Q9Y4A5](http://www.uniprot.org/uniprot/Q9Y4A5) [Eukaryota] Transformation/transcription domain-associated protein [Metazoa][Homo sapiens] (length=3859 residues).

Site : 3850- 3855, Identity

aaansldnlc_RMDPAW_hpwl›

--------------------------------------------------------------------------------

[NPSA](https://npsa-prabi.ibcp.fr/cgi-bin/seq_methonseq.pl?seqid=Q9Y4M8&db=unipsp&process=5be3cc137c874645&seqtype=1) [gnl|unipsp|Q9Y4M8](http://www.uniprot.org/uniprot/Q9Y4M8) [Eukaryota] Putative uncharacterized protein encoded by LINC00588 [Metazoa][Homo sapiens] (length=146 residues).

Site : 63- 68, Identity

rprslgagqg_REDPSW_eggalgdlka

--------------------------------------------------------------------------------

[NPSA](https://npsa-prabi.ibcp.fr/cgi-bin/seq_methonseq.pl?seqid=Q9Y4R8&db=unipsp&process=5be3cc137c874646&seqtype=1) [gnl|unipsp|Q9Y4R8](http://www.uniprot.org/uniprot/Q9Y4R8) [Eukaryota] Telomere length regulation protein TEL2 homolog [Metazoa][Homo sapiens] (length=837 residues).

Site : 69- 74, Identity

fspvlrclas_RLSPAW_lellphgrle

--------------------------------------------------------------------------------

[NPSA](https://npsa-prabi.ibcp.fr/cgi-bin/seq_methonseq.pl?seqid=Q9Y5X5&db=unipsp&process=5be3cc137c874647&seqtype=1) [gnl|unipsp|Q9Y5X5](http://www.uniprot.org/uniprot/Q9Y5X5) [Eukaryota] Neuropeptide FF receptor 2 [Metazoa][Homo sapiens] (length=522 residues).

Site : 39- 44, Identity

rerralsvqq_RGGPAW_sgslewsrqs

--------------------------------------------------------------------------------

[NPSA](https://npsa-prabi.ibcp.fr/cgi-bin/seq_methonseq.pl?seqid=Q9Y6K0&db=unipsp&process=5be3cc137c874648&seqtype=1) [gnl|unipsp|Q9Y6K0](http://www.uniprot.org/uniprot/Q9Y6K0) [Eukaryota] Choline/ethanolaminephosphotransferase 1 [Metazoa][Homo sapiens] (length=416 residues).

Site : 77- 82, Identity

lmqgywewlv_RRVPSW_iapnlitiig

--------------------------------------------------------------------------------

[NPSA](https://npsa-prabi.ibcp.fr/cgi-bin/seq_methonseq.pl?seqid=Q9Y6M7&db=unipsp&process=5be3cc137c874649&seqtype=1) [gnl|unipsp|Q9Y6M7](http://www.uniprot.org/uniprot/Q9Y6M7) [Eukaryota] Sodium bicarbonate cotransporter 3 [Metazoa][Homo sapiens] (length=1214 residues).

Site : 1075- 1080, Identity

akhqpdliyl_RYVPLW_kvhiftviql
